# Pleiotropic Effects of Trait-Associated Genetic Variation on DNA Methylation: Utility for Refining GWAS Loci

**DOI:** 10.1016/j.ajhg.2017.04.013

**Published:** 2017-05-18

**Authors:** Eilis Hannon, Mike Weedon, Nicholas Bray, Michael O’Donovan, Jonathan Mill

**Affiliations:** 1 University of Exeter Medical School, Exeter EX2 5DW, UK; 2 MRC Centre for Neuropsychiatric Genetics and Genomics, Cardiff University School of Medicine, Cardiff CF24 4HQ, UK

**Keywords:** genetics, DNA methylation, epigenetics, genome-wide association study, GWAS, complex trait, disease, pleiotropy, blood, brain

## Abstract

Most genetic variants identified in genome-wide association studies (GWASs) of complex traits are thought to act by affecting gene regulation rather than directly altering the protein product. As a consequence, the actual genes involved in disease are not necessarily the most proximal to the associated variants. By integrating data from GWAS analyses with those from genetic studies of regulatory variation, it is possible to identify variants pleiotropically associated with both a complex trait and measures of gene regulation. In this study, we used summary-data-based Mendelian randomization (SMR), a method developed to identify variants pleiotropically associated with both complex traits and gene expression, to identify variants associated with complex traits and DNA methylation. We used large DNA methylation quantitative trait locus (mQTL) datasets generated from two different tissues (blood and fetal brain) to prioritize genes for >40 complex traits with robust GWAS data and found considerable overlap with the results of SMR analyses performed with expression QTL (eQTL) data. We identified multiple examples of variable DNA methylation associated with GWAS variants for a range of complex traits, demonstrating the utility of this approach for refining genetic association signals.

## Main Text

There has been major progress in the identification of genetic variants influencing a diverse range of complex human phenotypes, including anthropometric measures (e.g., height and weight),^1^, ^2^ cardiovascular disease,^3^, ^4^ inflammatory disorders,^5^ neurological diseases,^6^, ^7^ and psychiatric illness.^8^, ^9^, ^10^ The challenge is now to improve our understanding of the biological effects of these genetic risk factors, especially because the actual genes involved in mediating phenotypic variation are not necessarily the most proximal to the lead SNPs identified in genome-wide association studies (GWASs). Supported by the observation that GWAS variants are preferentially located in enhancers and regions of open chromatin,^11^, ^12^ the majority of common genetic risk factors are predicted to influence gene regulation rather than directly affect the coding sequences of transcribed proteins.^13^

Expression quantitative trait loci (eQTLs) have been successfully used for investigating the functional consequences of GWAS variants.^14^, ^15^ The co-localization of GWAS and eQTL variants, however, is not sufficient to show that the overlapping association signals are causally related, given that the association signals might be tagging different causal variants in the same linkage disequilibrium (LD) block. Recently, an approach called summary-data-based Mendelian randomization (SMR) was proposed as a strategy for identifying overlapping genetic signals associated with both phenotypic and transcriptional variation and subsequently distinguishing pleiotropic effects (i.e., where the same variant influences both outcomes, although not necessarily dependently) from those that are artifacts of LD.^16^ Genetic effects on gene expression can be mediated by epigenetic processes such as changes in DNA methylation, a cytosine modification that has an essential role in mammalian development.^17^ We have previously demonstrated the utility of DNA methylation QTLs (mQTLs) for interpreting GWAS findings by identifying specific examples where genetic polymorphisms associated with schizophrenia (MIM: 181500) co-localize with variants associated with DNA methylation.^18^, ^19^ In this study, we applied the SMR approach to test 35,263 DNA methylation sites against 43 complex phenotypes with robust GWAS data (Table S1) by using mQTLs identified in our recent analysis of methylomic variation in whole blood and imputed SNP genotypes (n = 639; mQTL p < 1 × 10^−10^; a full description of this dataset, referred to as phase 1, can be found here^19^) in conjunction with publicly available summary data from a series of well-powered GWAS analyses.

The first stage of the SMR analysis identifies the most significantly associated SNP for a DNA methylation site (that is also present in the GWAS dataset) as an instrumental variable for testing for an association with a phenotype by the two-step least-squares (2SLS) approach, which uses the same SNP to compare the mQTL coefficients with those from a GWAS of the phenotype (Figure S1A). This approach identified 1,932 associations (p < 1.42 × 10^−6^ corrected for 35,263 DNA methylation sites) between 31 complex traits and 1,354 individual DNA methylation sites (Table S2 and Figure S2). Because these associations can be driven by two highly correlated but different causal variants for the GWAS trait and DNA methylation, the second stage of the SMR approach repeats the analysis with alternative SNPs associated with DNA methylation as the instrument and performs a HEIDI (heterogeneity in dependent instruments) test for heterogeneity in the resulting association statistics. If a single causal variant is associated with both the phenotype and DNA methylation, the association statistics will be identical regardless of the selected instrument (Figure S1B), and the HEIDI p value will be non-significant. In contrast, if two separate causal variants are each correlated with the instrument, there will be variation in the results from different instruments (Figure S1C), as indicated by a significant HEIDI p value. It should be noted that this approach is unable to distinguish these two scenarios if the two causal variants are in perfect LD and power is inversely proportional to the strength of the correlation between the two causal variants. Furthermore, the assumptions underlying Mendelian randomization^20^ also apply to SMR, and it is possible for variants to act through mechanisms such as horizontal pleiotropy.

By identifying non-significant heterogeneity (HEIDI p > 0.05), we identified a refined set of 625 associations between 28 complex traits and 440 DNA methylation sites (Table S2), which can be described as pleiotropic. We were able to test 581 of these associations with mQTLs generated from a second independent whole-blood dataset (n = 665; a description of this cohort, referred to as phase 2, can be found here^19^). A highly significant proportion (99.2%; sign test p = 1.47 × 10^−172^) had the same direction of association across the two datasets (Figure S3), and a large proportion (n = 337; 58.0%) satisfied the criteria for a pleiotropic association (p < 1.04 × 10^−6^ and HEIDI p > 0.05) in the replication dataset as well. Out of the GWAS traits tested, height was characterized by the most associations (n = 193), an unsurprising observation given that this was the most highly powered GWAS with the largest number of GWAS-significant loci (n = 423). Power for SMR analysis is influenced by the power of the GWAS, which differs for each trait considered, making comparisons between traits relatively difficult.

As demonstrated in its original implementation for eQTLs, the SMR approach based on mQTLs has the potential to nominate loci that currently do not have sufficient statistical power to obtain genome-wide significance on the basis of GWAS data alone but that represent candidates for future genetic studies (Table S3). Our SMR analysis of Tanner staging of puberty, for example, identified DNA methylation sites in nine independent loci (annotated to *APEH* [MIM: 102645], *SYNJ2* [MIM: 609410], *IDO2* [MIM: 612129], *PDZRN4* [MIM: 609730], *HTR2A* [MIM: 182135], *CTDP1* [MIM: 604927], *RAE1* [MIM: 603343], and non-genic regions on chromosomes 4 and 16) that do not have a genome-wide-significant (p < 5 × 10^−8^) variant within 0.5 Mb in the GWAS^21^ (Figure S4). In some genomic regions, DNA methylation sites annotated to different genes are associated with the same phenotype; for example, on chromosome 15, sites annotated to *CHRNA5* (MIM: 118505) and *PSMA4* (MIM: 176846) are associated with the number of cigarettes smoked per day (Figure S5), and on chromosome 17, sites annotated to *ERBB2* (MIM: 164870) and *PGAP3* (MIM: 611801) are associated with total cholesterol (Figure S6). Furthermore, 130 DNA methylation sites were found to be associated with multiple complex traits (ranging from two to six traits; Table S4). In many cases, these overlaps are consistent with either reported phenotypic correlations (e.g., cg24631222 and cg04140906 annotated to *CHRNA5* are associated with both schizophrenia and the number of cigarettes smoked per day [Figure S7], two traits that are epidemiologically linked^22^, ^23^) or shared genetic architecture (e.g., cg10583485, annotated to *DOCK7* [MIM: 615730] [Figure S8], is associated with LDL, triglycerides, and total cholesterol, three traits characterized by a strong genetic correlation^24^). Because genetic correlations could account for some of the overlap between traits, we factored in genetic correlations derived from LD score regression,^24^ which showed that 30 of the 70 pairs of traits with at least one associated DNA methylation site in common are actually characterized by a genetic correlation < 0.2 (Figure S9).

**Figure 1:**
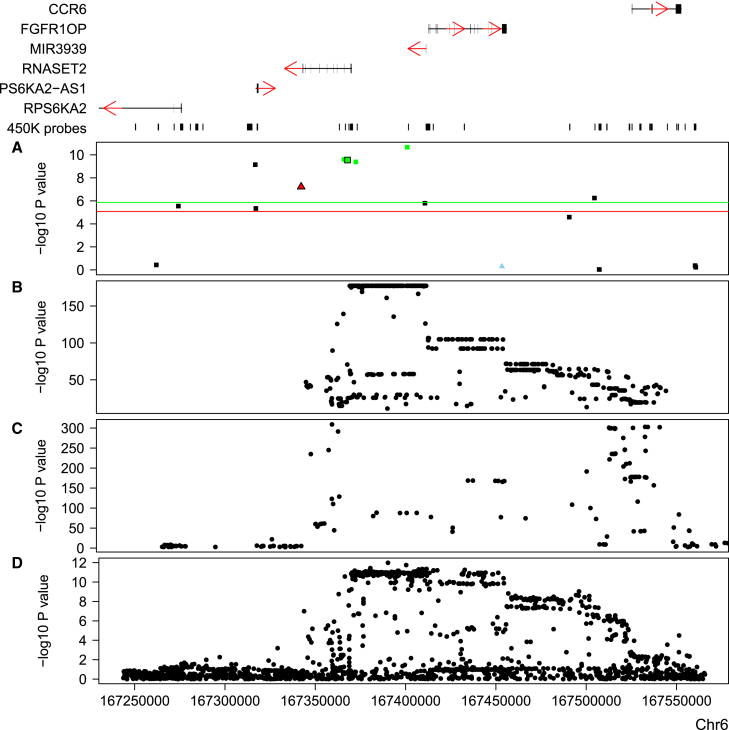
SMR Analysis Using mQTLs and eQTLs Implicates a Role for *RNASET2* in Crohn Disease Shown is a chromosome 6 genomic region (UCSC Genome Browser hg19: 167,243,095–167,565,882) identified in a recent Crohn disease GWAS performed by Liu et al.^5^ Genes located in this region are shown at the top; exons are indicated by thicker bars, and red arrows indicate the direction of transcription. DNA methylation sites interrogated by the Illumina 450K array are indicated by solid vertical lines underneath the genes. The four bottom panels depict the −log_10_ p value (y axis) against genomic location (x axis) from (A) SMR analysis (black squares represent Illumina 450K array DNA methylation sites, blue triangles represent gene expression probes, and green and red coloring highlight those with a non-significant HEIDI test for DNA methylation and gene expression, respectively), (B) blood mQTL (n = 639) results for the DNA methylation site cg25258033 (outlined in black in A), (C) blood eQTL (n = 5,311) results for ILMN1671565 (outlined in black in A), and (D) the Crohn disease GWAS performed by Liu et al.^5^

Multiple DNA methylation sites can be annotated to a single gene, and we identified a total of 337 gene-trait pleiotropic associations with a mean of 1.46 sites associated per gene (range = 1–11). These overlapping associations between a particular complex trait and a gene would not necessarily be expected to be associated in the same direction given that correlation of DNA methylation across a gene is not always positive, and they were not for 20 of the 31 gene-trait associations involving genes with multiple annotated DNA methylation sites. To add further support to the genes prioritized at GWAS loci with the use of blood mQTL data, we aligned these results with SMR analyses performed on publically available whole-blood eQTL data (n = 5,311; p < 5 × 10^−8^) described in detail in a recent paper by Westra et al.^15^ We identified an overlapping set of 2,724 genes that were (1) annotated to DNA methylation sites influenced by significant mQTLs (involving 7,722 distinct DNA methylation sites) and (2) also transcriptionally influenced by variation at significant eQTLs (involving 2,770 gene expression microarray probes), making them suitable for testing in the SMR framework. It should be noted that one limitation to assessing the relationship between mQTLs and eQTLs is that DNA methylation sites, like SNPs, are annotated to genes according to their location; therefore, a lack of overlap in the associations with a particular gene from the SMR analyses between DNA methylation and gene expression should not necessarily be interpreted as inconsistent evidence. Furthermore, the differences in the sample sizes used for generating the mQTL and eQTL datasets could result in different levels of statistical power to detect QTLs. Of the 337 pleiotropic gene-trait associations identified with mQTLs, 86 (25.5%) were also tested with eQTLs in the SMR framework (Figure S10). Of these, 27 (31.4%) involving 17 complex traits associated with expression at 16 genes also met the criteria for representing pleiotropic associations between the trait and gene expression (SMR p < 8.38 × 10^−6^ corrected for 5,966 gene expression probes and HEIDI p > 0.05) (Table S5). An example of overlapping mQTL and eQTL signals for *RNASET2* (MIM: 612944) on chromosome 6 is presented in Figure 1; both *RNASET2* expression (SMR p = 6.04 × 10^−8^) and DNA methylation at two CpG sites in the first intron of the gene (cg25258033: SMR p = 2.84 × 10^−10^; cg25258033: SMR p = 2.50 × 10^−10^) are associated with Crohn disease (MIM: 266600).

**Figure 2:**
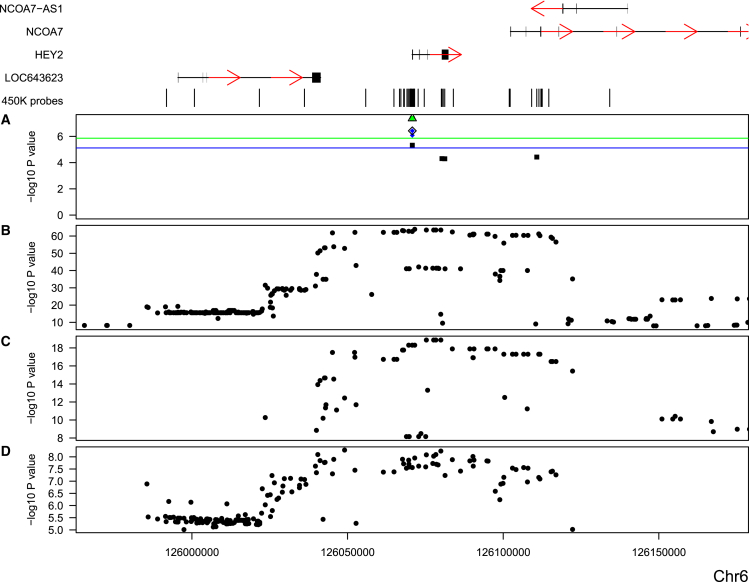
SMR Analysis Using Whole-Blood and Fetal Brain mQTL Data Implicates a Role for *HEY2* in Migraine Shown is a chromosome 6 genomic region (UCSC Genome Browser hg19: 125,970,800–126,170,800) identified in a recent migraine GWAS performed by Gormley et al.^25^ Genes located in this region are shown at the top; exons are indicated by thicker bars, and red arrows indicate the direction of transcription. The four bottom panels depict the −log_10_ p value (y axis) against genomic location (x axis) from (A) SMR analysis (points represent DNA methylation sites interrogated by the Illumina 450K array, squares and diamonds indicate SMR tests from blood and fetal brain mQTLs, respectively, and green squares and blue diamonds highlight those with a non-significant HEIDI test for blood and fetal brain, respectively), mQTL results for the DNA methylation site cg05901451 (outlined in black in A) in (B) blood (n = 639) and (C) fetal brain (n = 166), and (D) the migraine GWAS performed by Gormley et al. (n = 59,674 case and 316,078 control samples).^25^

Given the tissue-specific and developmentally dynamic nature of gene regulation, we were next interested in examining the consistency of our findings in a different tissue. So, we repeated the SMR analysis on mQTLs identified in our recent analysis of human fetal brain (n = 166; mQTL p < 1 × 10^−8^; a detailed description of this dataset can be found here^18^). The majority (75.4%) of SNP-DNA methylation relationships identified for SMR analysis in whole blood are characterized by a consistent direction of effect when tested in fetal brain (sign test p = 4.94 × 10^−324^; Figure S11). Despite the strong concordance of mQTL effects across tissues, the smaller number of samples used for generating the fetal brain dataset (n = 166) means that only a subset (4,691 [13.3%]) of these mQTL associations passed our mQTL significance threshold (p < 1 × 10^−8^) and were included in the subsequent SMR analyses; almost all of these (96.0%; sign test p < 2.2 × 10^−308^) were characterized by the same direction of effect in both tissues (Figure S12). Of the 625 pleiotropic associations identified with whole-blood mQTLs, 84 (13.5%) involved a DNA methylation site that also had a significant fetal brain mQTL (p < 1 × 10^−8^), meaning it could be tested with the SMR framework (Figure S13). Of these 84 pleiotropic associations, 35 (41.7%) met the criteria (i.e., SMR p < 5.40 × 10^−6^ corrected for 9,265 DNA methylation sites tested and HEIDI p > 0.05) for also having a pleiotropic association involving nine complex traits in fetal brain (Table S6). Whereas six (17.1%) of the site-trait associations involved brain-related phenotypes (five for schizophrenia and one for migraine [MIM: 157300]), the majority (82.9%) involved traits that are presumed to affect other tissues (e.g., total cholesterol and Crohn disease), suggesting that effects are common across tissues. Figure 2 summarizes SMR analysis across the *HEY2*-*NOCA7* region on chromosome 6, which was implicated in a recent GWAS of migraine.^25^ Manhattan plots for the genetic analysis of cg05901451, located in the 5′ UTR of *HEY2* (MIM: 604674), in whole blood and fetal brain show a profile highly comparable to that of the migraine GWAS, consistent with overlapping genetic signals influencing DNA methylation in both tissues and migraine.^25^

**Figure 3:**
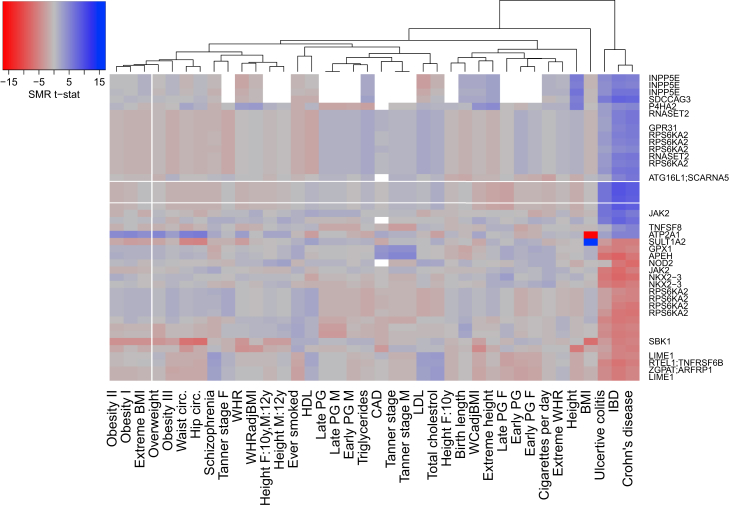
Heatmap of the SMR Results for 32 DNA Methylation Sites Associated with Crohn Disease across 38 GWAS Datasets Each square in the heatmap represents the t-statistic (b_SMR/se_SMR) of the GWAS trait (columns) for a DNA methylation site (row; n = 32) associated with Crohn disease. Only phenotypes (n = 38) tested against at least 20,000 DNA methylation sites were included in this comparison. SMR p < 1.38 × 10^−6^ and HEIDI p > 0.05.

Finally, comparing the SMR results across multiple complex traits gives a potential insight into shared pleiotropic associations between pairs of traits. We performed hierarchical clustering of SMR results for 38 complex traits, selected because they were tested against a minimum of 20,000 DNA methylation sites, to identify consistent signatures (Figure S14). Figure 3, for example, depicts the association statistics for 43 DNA methylation sites associated with Crohn disease (SMR p < 1.42 × 10^−6^) across all 38 phenotypes; interestingly, we observed a highly concordant profile between Crohn disease and ulcerative colitis across all associated sites, consistent with the strong genetic correlation between these traits (Figure S9). The SMR results might highlight which genes are characterized by shared effects between traits. There is also a notable overlap with BMI, waist, and hip circumference at specific loci (i.e., *ATP2A1* [MIM: 108730], *SULT1A2* [MIM: 601292], and *SBK1* [MIM: 300374]), an interesting observation given the negligible genetic correlations between these traits and Crohn disease.

Together, these analyses demonstrate the utility of the SMR approach for identifying instances where complex traits and variable DNA methylation are pleiotropically associated with genetic variation. This approach could facilitate our understanding of the functional consequences of genetic risk variants for a range of complex traits and facilitate the localization and prioritization of specific genes within genomic regions identified by GWASs.

## Web Resources

ChunkChromosome, http://genome.sph.umich.edu/wiki/ChunkChromosome
Fetal brain mQTL data, http://epigenetics.essex.ac.uk/mQTL/All_Imputed_BonfSignificant_mQTLs.csv.gz
OMIM, http://www.omim.org
SMR, http://cnsgenomics.com/software/smr/download.html
UCSC Genome Browser, https://genome.ucsc.edu/
Whole-blood eQTL data, http://cnsgenomics.com/software/smr/westra_eqtl_hg19.zip
Whole-blood mQTL data, http://epigenetics.essex.ac.uk/schizophrenia/BloodmQTL.csv.tgz

## Supplemental Data

Document S1. Figures S1–S12

Table S1. Details of All Publically Available GWAS Results Included in SMR Analysis

Table S2. Results from SMR Analysis for All Significant Associations between Complex Traits and DNA Methylation Significance: p < 1.42 × 10^−6^.

Table S3. Associations from SMR Analysis where No SNP within 0.5 Mb Has Obtained Genome-wide Significance in GWASs These loci therefore represent associations worthy of further investigation for that complex trait. Significance: p < 5 × 10^−8^.

Table S4. Overlapping Associations with Specific DNA Methylation Sites between Complex Traits Entries above the diagonal detail the specific Illumina 450K array probe, and entries below the diagonal detail the gene that the probe is located within.

Table S5. Gene-Trait Associations Identified with SMR Analysis Using Whole-Blood mQTLs and Blood eQTLs

Table S6. DNA Methylation Sites Associated through mQTLs Identified in Both Whole Blood and Fetal Brain

Document S2. Article plus Supplemental Data
