# Supplementary material for: Pleiotropic Effects of Trait-Associated Genetic Variation on DNA Methylation: Utility for Refining GWAS Loci
Source: Am J Hum Genet. 2017 May 18;100(6):954–9. doi: 10.1016/j.ajhg.2017.04.013 (PMC5473725; doi:10.1016/j.ajhg.2017.04.013)
Supplement: Document S1. Figures S1–S12 [file mmc1.pdf]

The American Journal of Human Genetics, Volume *100*

Supplemental Data

Pleiotropic Effects of Trait-Associated Genetic Variation on DNA Methylation: Utility for Refining GWAS Loci

Eilis Hannon, Mike Weedon, Nicholas Bray, Michael O'Donovan, and Jonathan Mill

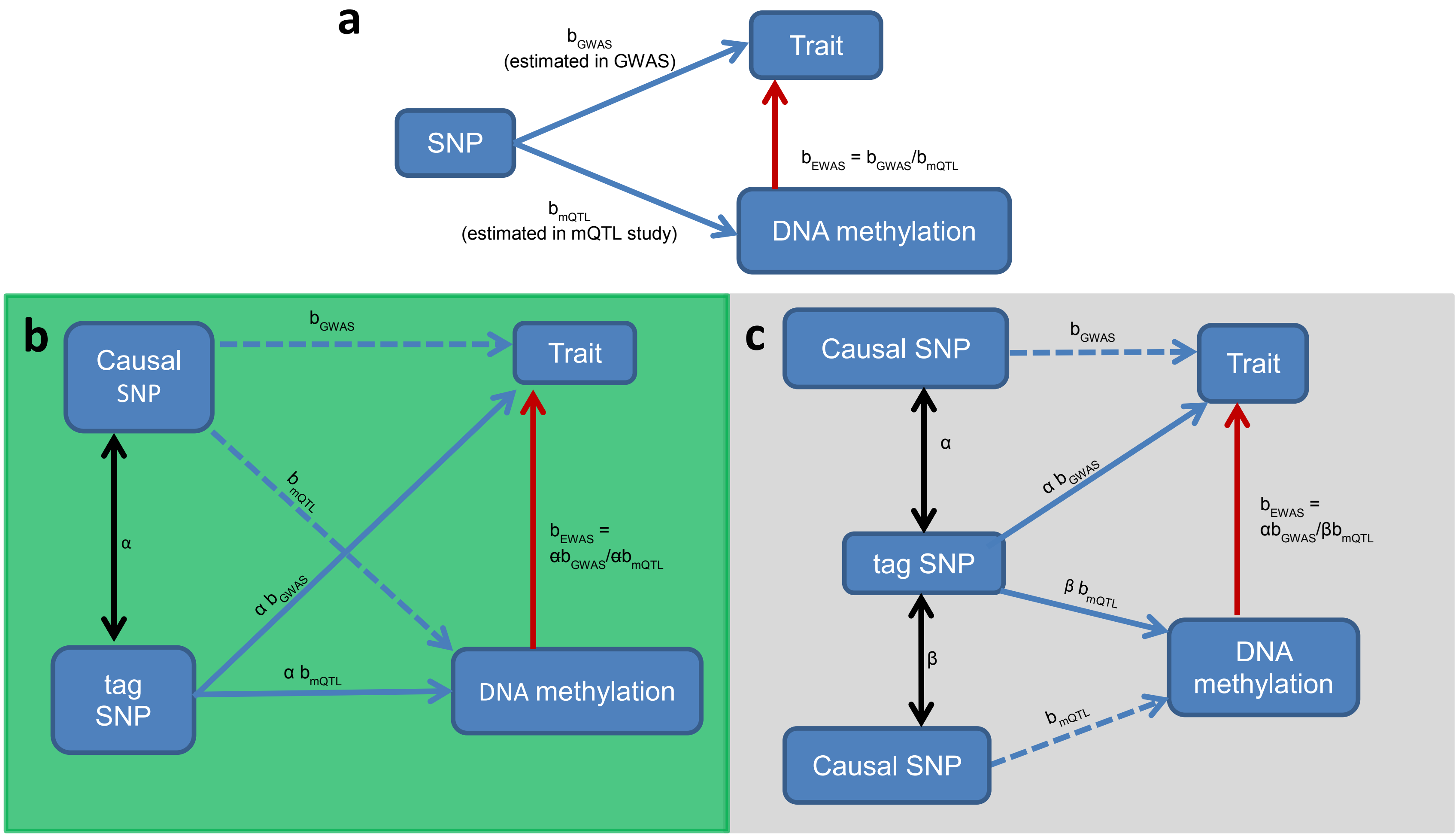

Figure S1: Schematic of the SMR analysis approach used in this study. Panel **a**) demonstrates the first stage of the SMR analysis, which tests for an association between DNA methylation and a trait of interest. Blue solid arrows represent known information taken from GWAS or mQTL results, while red arrows indicate the relationship being derived. The second stage of the SMR analysis aims to distinguish between two scenarios, pleiotropy and linkage, depicted in panels **b**) and **c**) respectively. In these figures dashed blue arrows indicate the true causal associations estimated via "tag SNPs". "tag SNPs" are highly correlated (represented by solid black arrows) with the "causal SNP" quantified as α or β. In panel **b**) as the same causal SNP is associated with both the trait of interest and DNA methylation at a specific site; there is only one correlation statistic (α), which is cancelled out when estimating the effect b_{EWAS}. In contrast, in panel **c**) there are distinct causal SNPs for DNA methylation and the trait, and therefore two correlations with the "tag SNP" (α and β), which do not cancel each other out. Therefore, the estimate of b_{EWAS} will exhibit heterogeneity when different "tag SNPs" are tested, whereas in the scenario depicted in panel **b**) the estimate of b_{EWAS} will be consistent regardless of the choice of "tag SNP". GWAS- genome-wide association analysis study; EWAS- epigenome wide association analysis study.

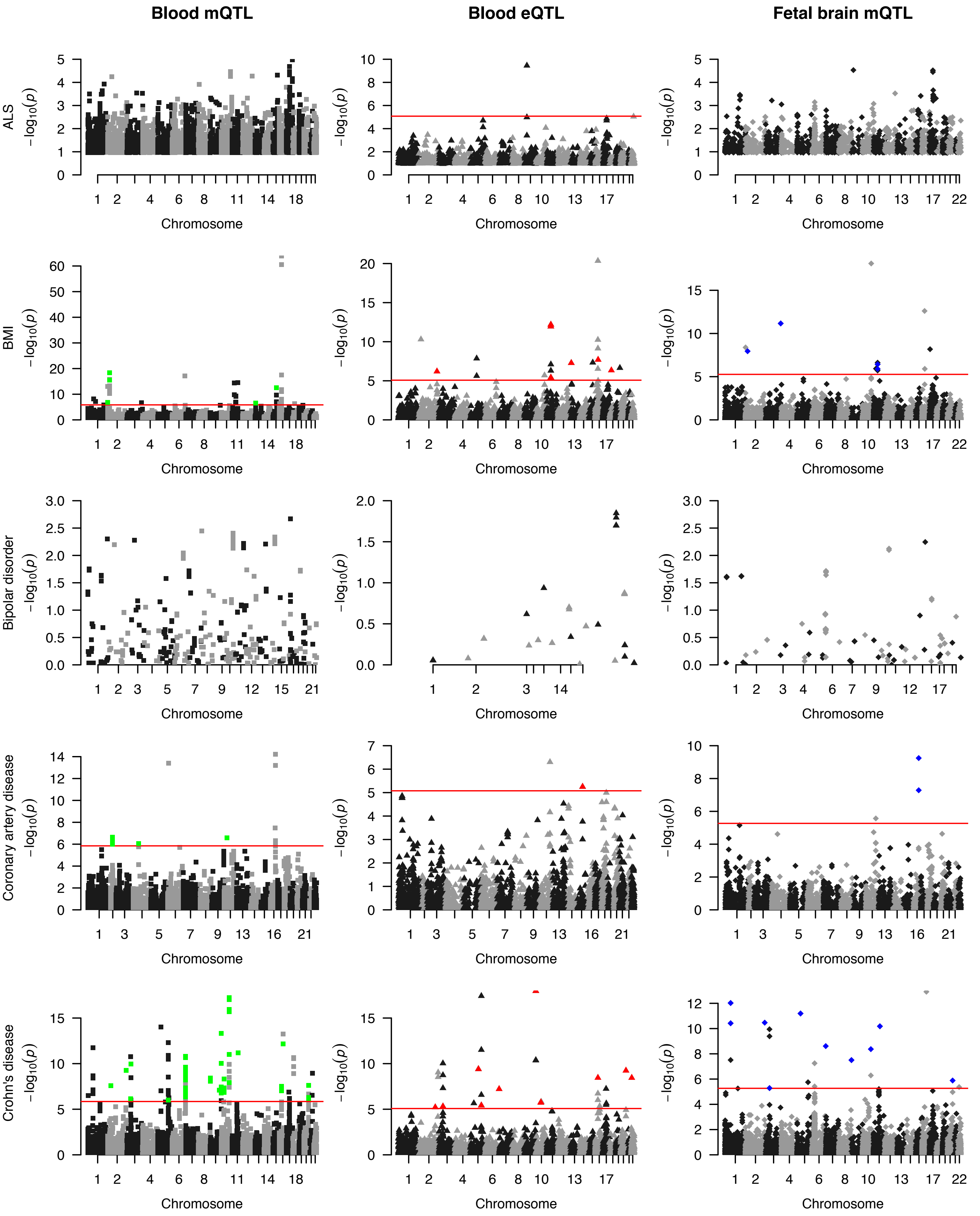
Blood mQTL
Blood eQTL
Fetal brain mQTL
ALS
BMI
Bipolar disorder
Coronary artery disease
Crohn's disease
$-\log_{10}(p)$
Chromosome

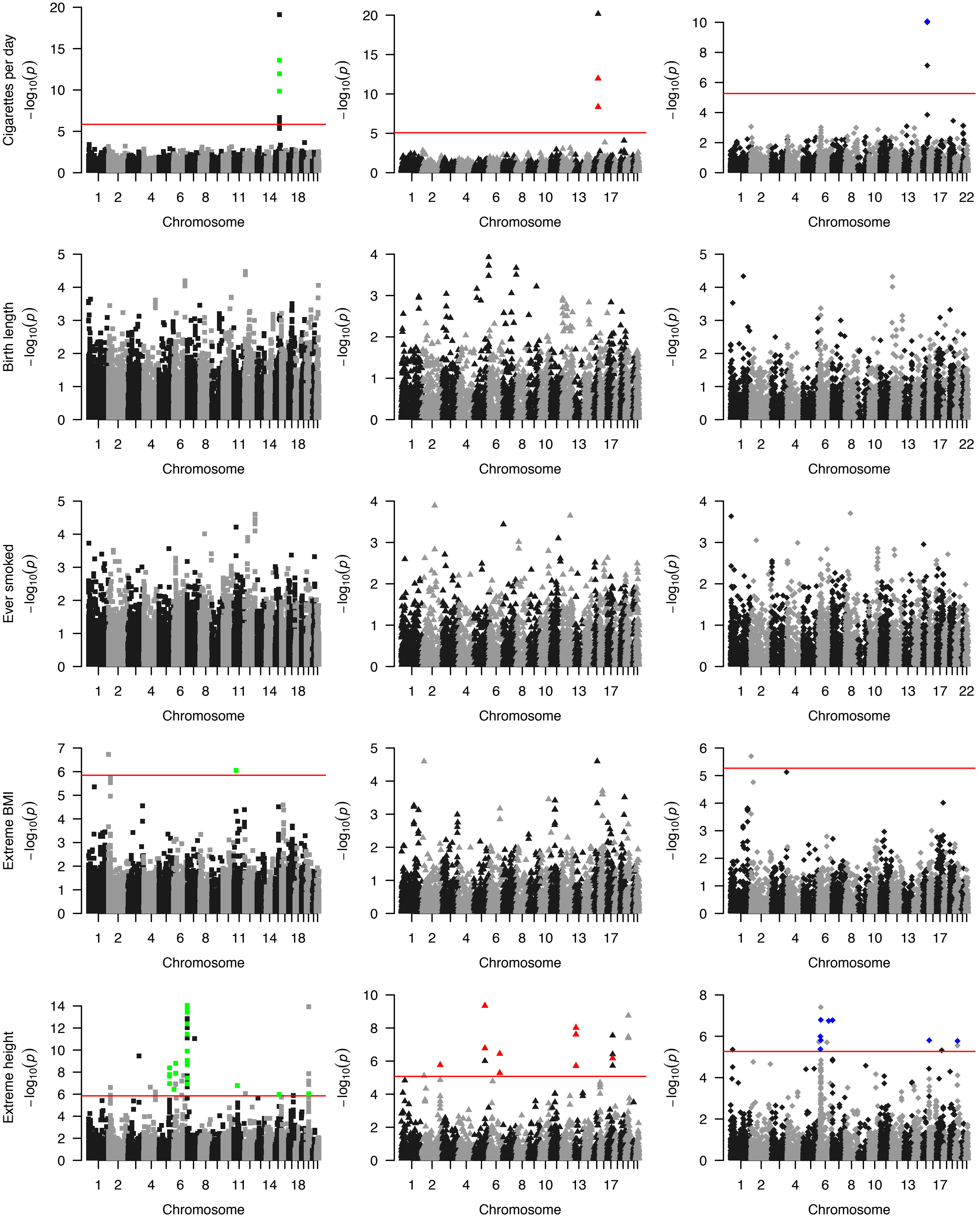
Cigarettes per day
Birth length
Ever smoked
Extreme BMI
Extreme height
$-\log_{10}(p)$
Chromosome
1 2 4 6 8 11 14 18
1 2 4 6 8 10 13 17
1 2 4 6 8 10 13 17 22

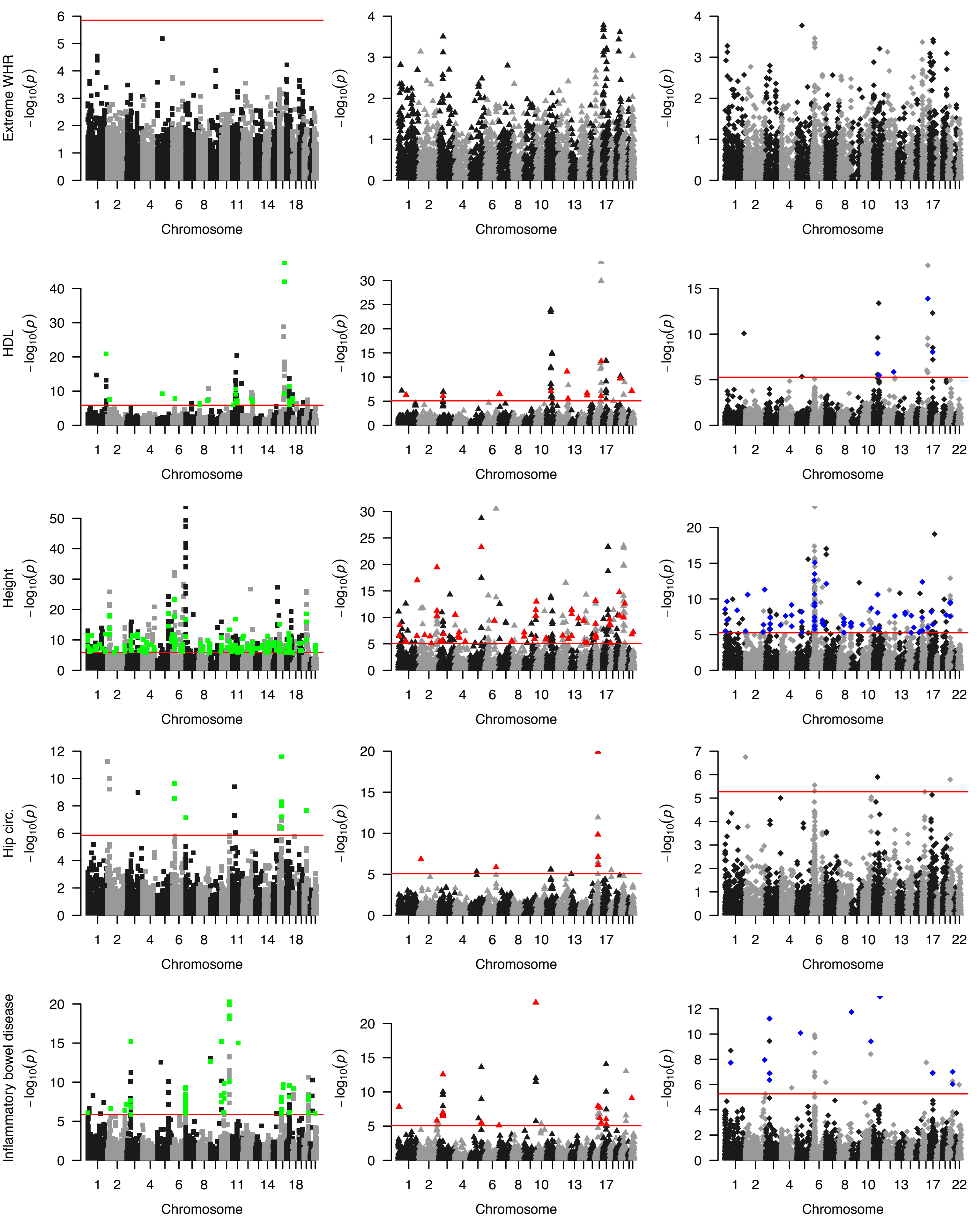

Extreme WHR
HDL
Height
Hip circ.
Inflammatory bowel disease
−log10(p)
Chromosome

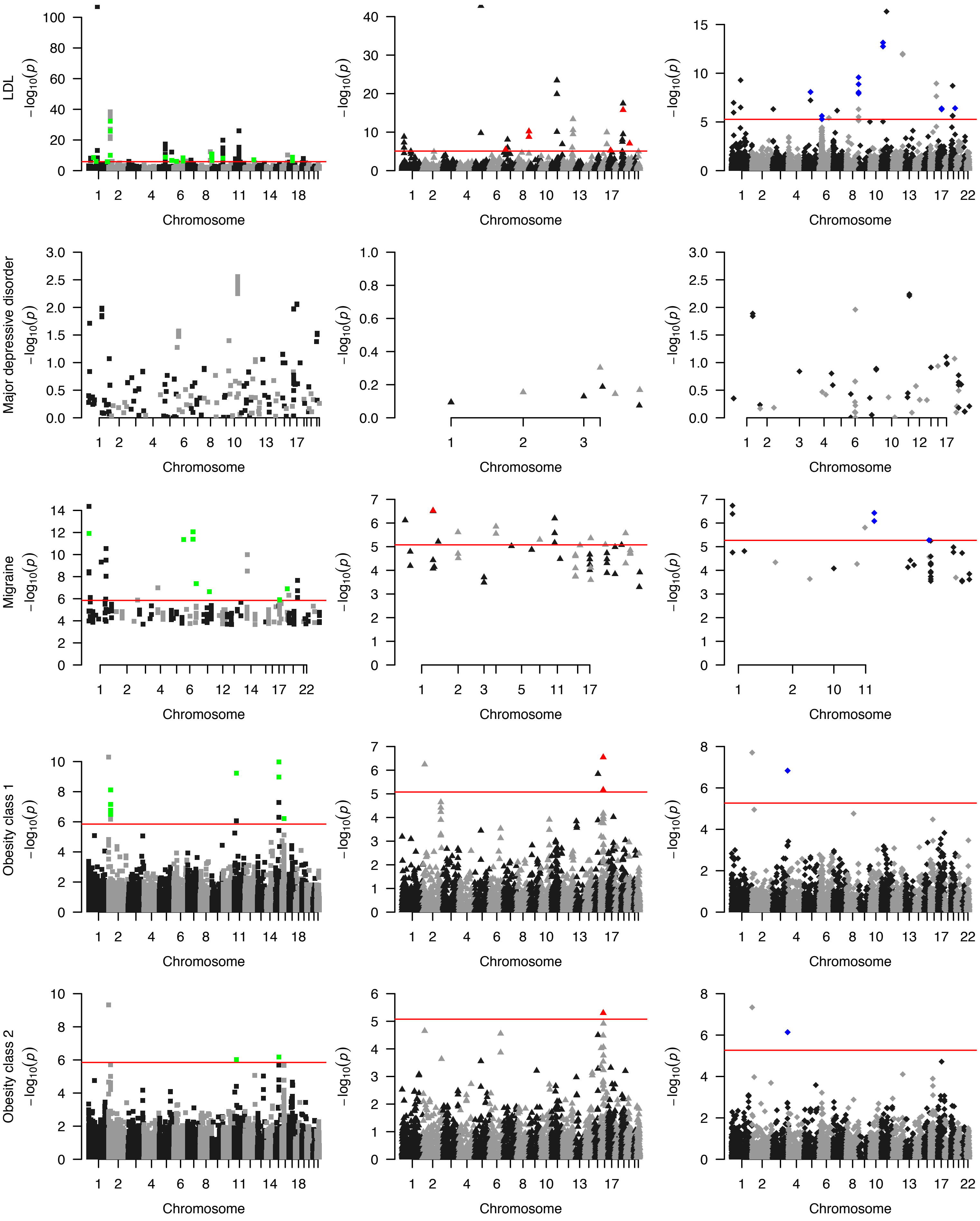

LDL
Major depressive disorder
Migraine
Obesity class 1
Obesity class 2
$-\log_{10}(p)$
Chromosome

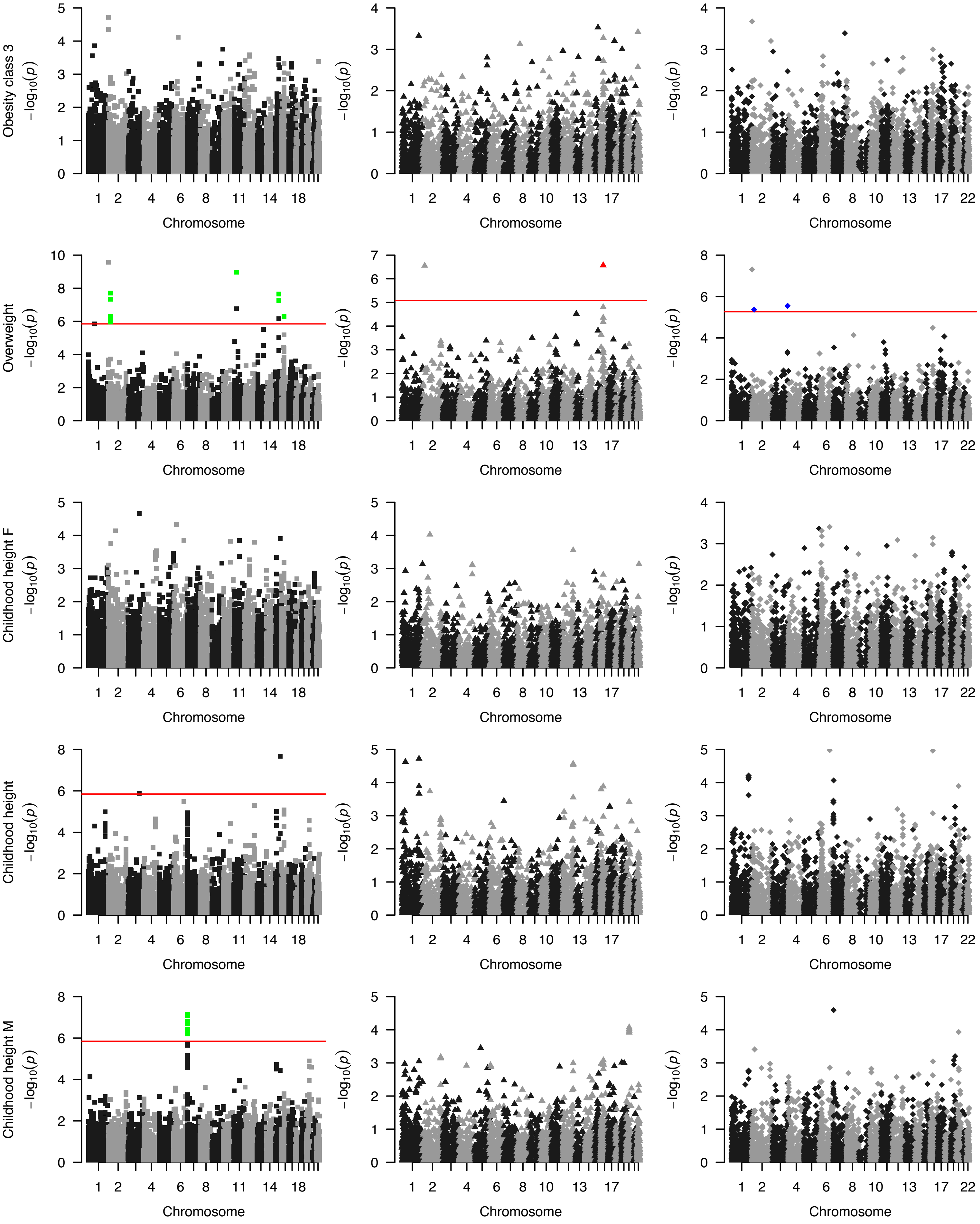

Obesity class 3
Overweight
Childhood height F
Childhood height
Childhood height M
−log10(p)
Chromosome
1 2 4 6 8 11 14 18
1 2 4 6 8 10 13 17
1 2 4 6 8 10 13 17 22

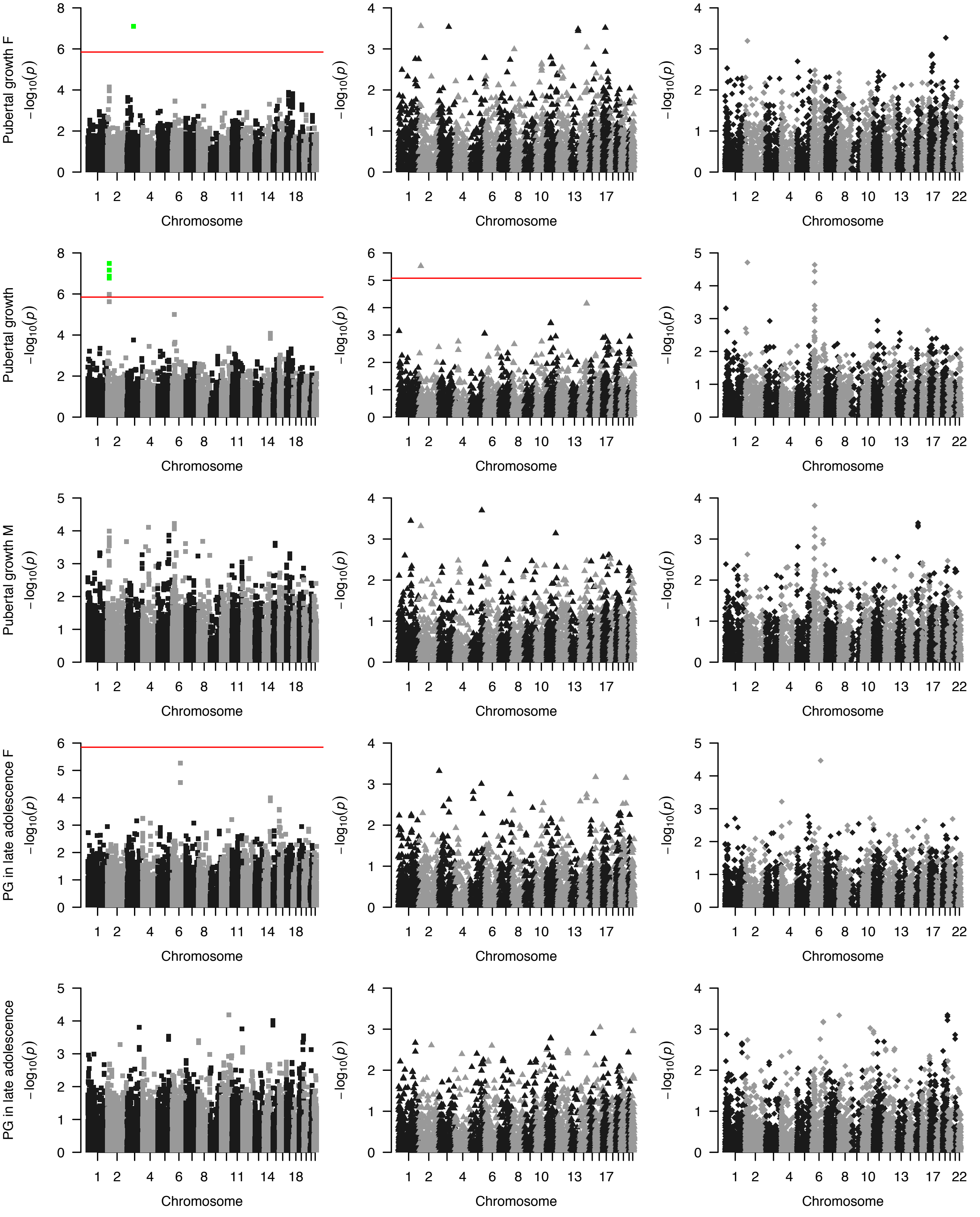

Pubertal growth F
Pubertal growth
Pubertal growth M
PG in late adolescence F
PG in late adolescence
−log10(p)
Chromosome

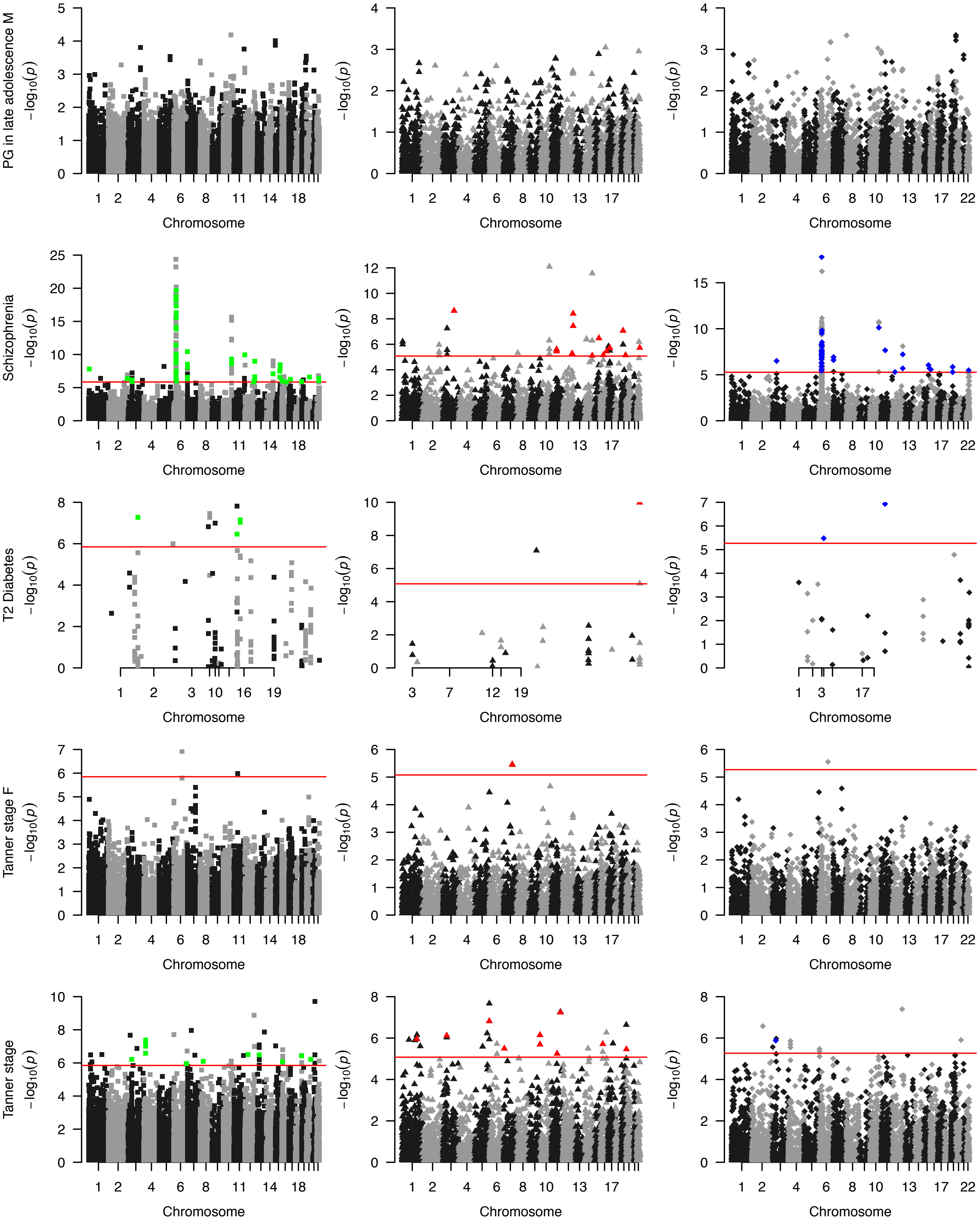
PG in late adolescence M
Schizophrenia
T2 Diabetes
Tanner stage F
Tanner stage
$-\log_{10}(p)$
Chromosome

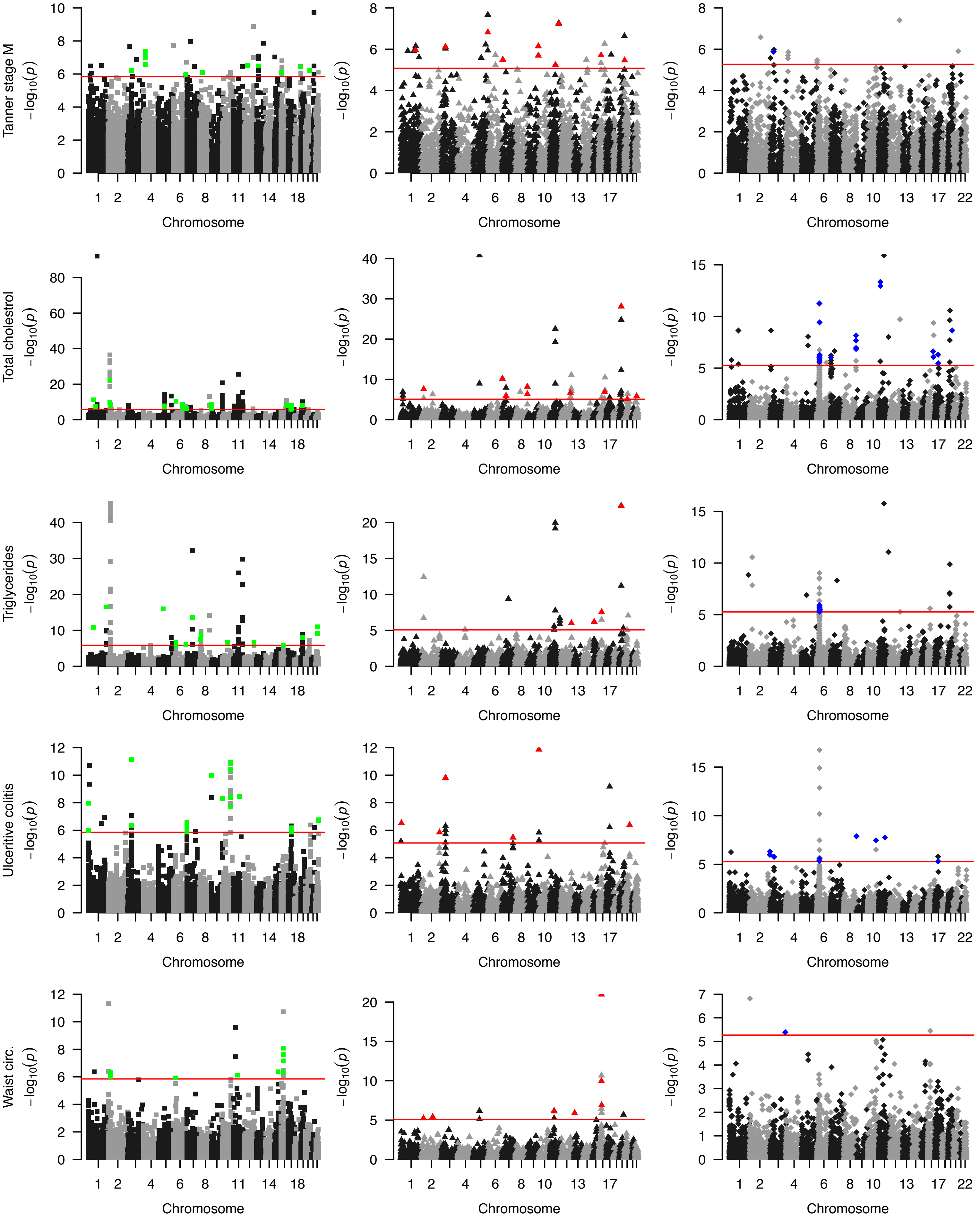

Tanner stage M
Total cholestrol
Triglycerides
Ulceritive colitis
Waist circ.
$-\log_{10}(p)$
Chromosome

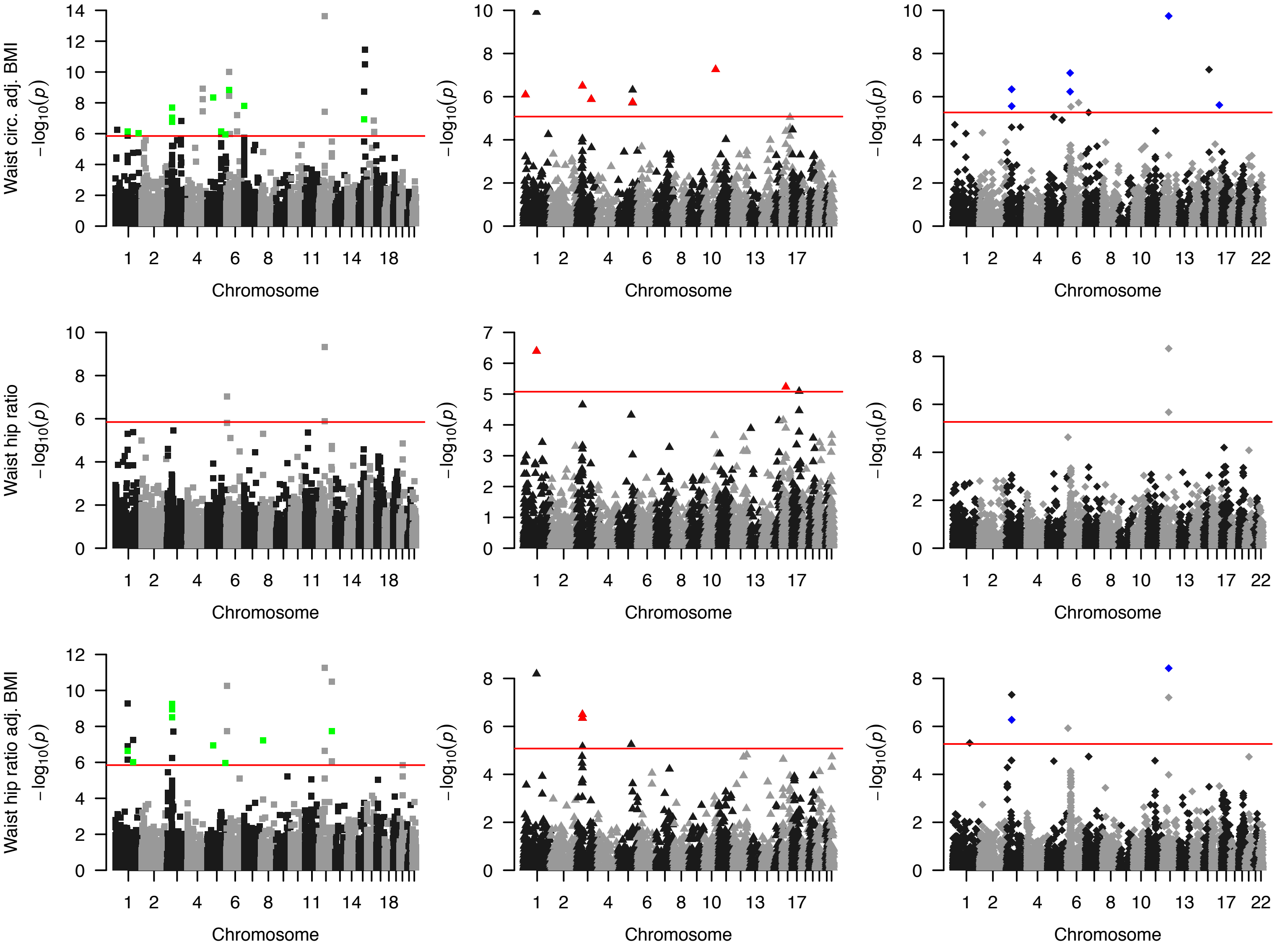

Waist circ. adj. BMI
Waist hip ratio
Waist hip ratio adj. BMI
$-\log_{10}(p)$
Chromosome

Figure S2: Manhattan plots of Summarised Mendelian Randomisation (SMR) tests for pleiotropic effects between complex traits and DNA methylation. Shown on the y-axis of each plot is the –log10 *P*-value from the SMR analysis. Panels on the left display results using DNA methylation quantitative trait loci (mQTL) generated from blood, panels in the middle display results using gene expression quantitative trait loci (eQTL) generated from blood and panels on the right display results using mQTL in fetal brain tissue. Each point represents an SMR test for a particular DNA methylation site or gene expression probe. The red horizontal line represents the genome-wide multiple testing significance threshold (blood mQTL: $P < 1.42 \times 10^{-6}$; blood eQTL: $P < 8.38 \times 10^{-6}$; fetal brain mQTL: $P < 2.14 \times 10^{-6}$); green, red and blue points highlight the significant SMR tests from blood mQTL, blood eQTL and fetal brain mQTL, respectively, which are not characterized by significant heterogeneity (i.e. $P > 0.05$), indicating pleiotropic relationships between that trait and either DNA methylation or gene expression.

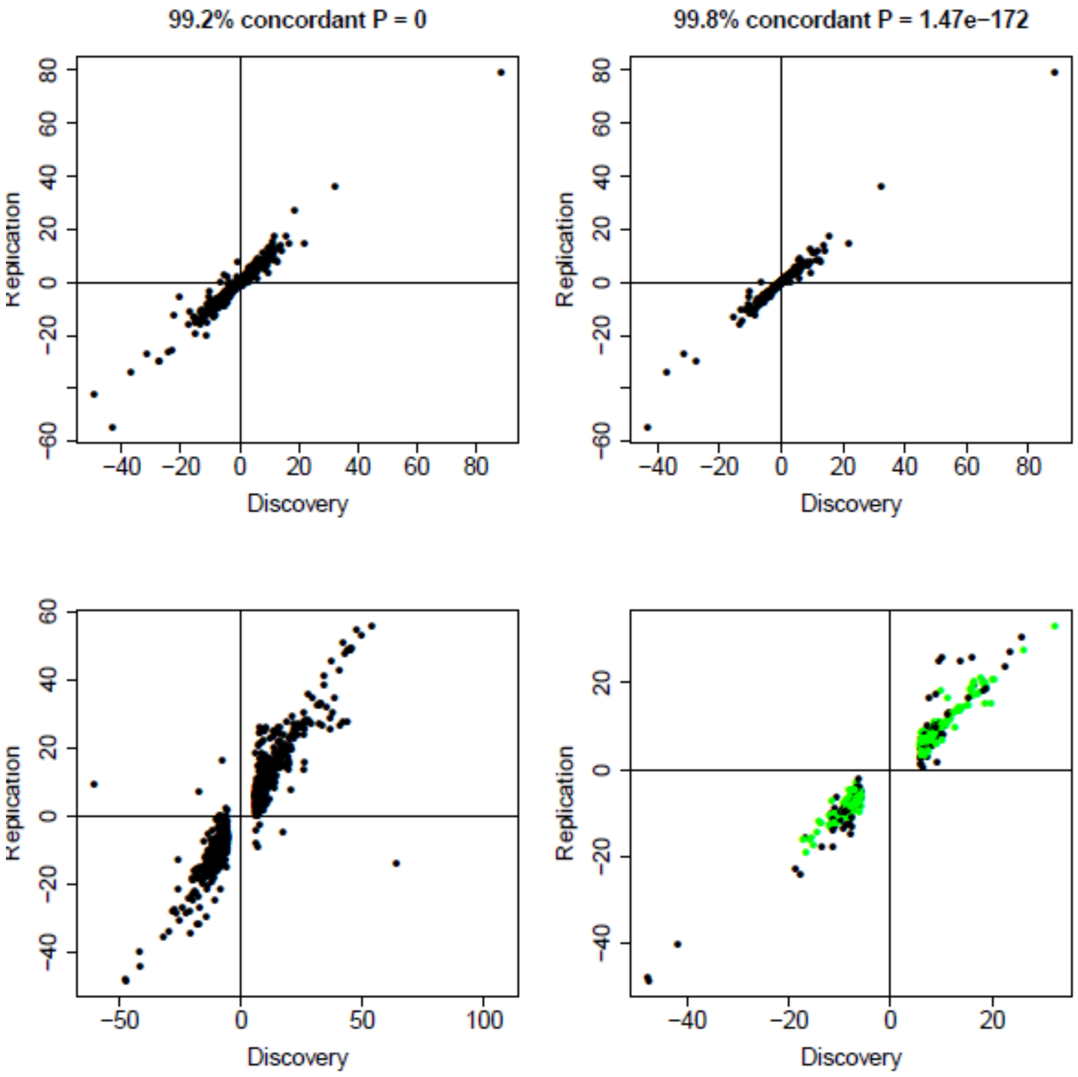

Figure S3: SMR results replicate in a second mQTL dataset. Shown are scatterplots of the association between DNA methylation sites and complex traits comparing results from our original ‘discovery’ cohort (X-axis) and our ‘replication’ cohort (Y-axis). The top row plots the regression coefficient (b_SMR) and the bottom row plots the signed log SMR P value for the discovery cohort and replication cohorts. Panels on the left contains all 1,723 associations identified in the discovery cohort (SMR P < 1.42 x 10-6) and tested in the replication cohort; panels on the right contains all 581 pleiotropic associations identified in the discovery cohort (SMR P < 1.42 x 10-6 and HEIDI P > 0.05) and tested in replication cohort. In the bottom right-hand panel, the green points indicate the sites with HEIDI P > 0.05 in the replication dataset. Details of the discovery and replication cohorts can be found in this manuscript[1] refered to as Phase 1 and Phase 2 respectively.

a

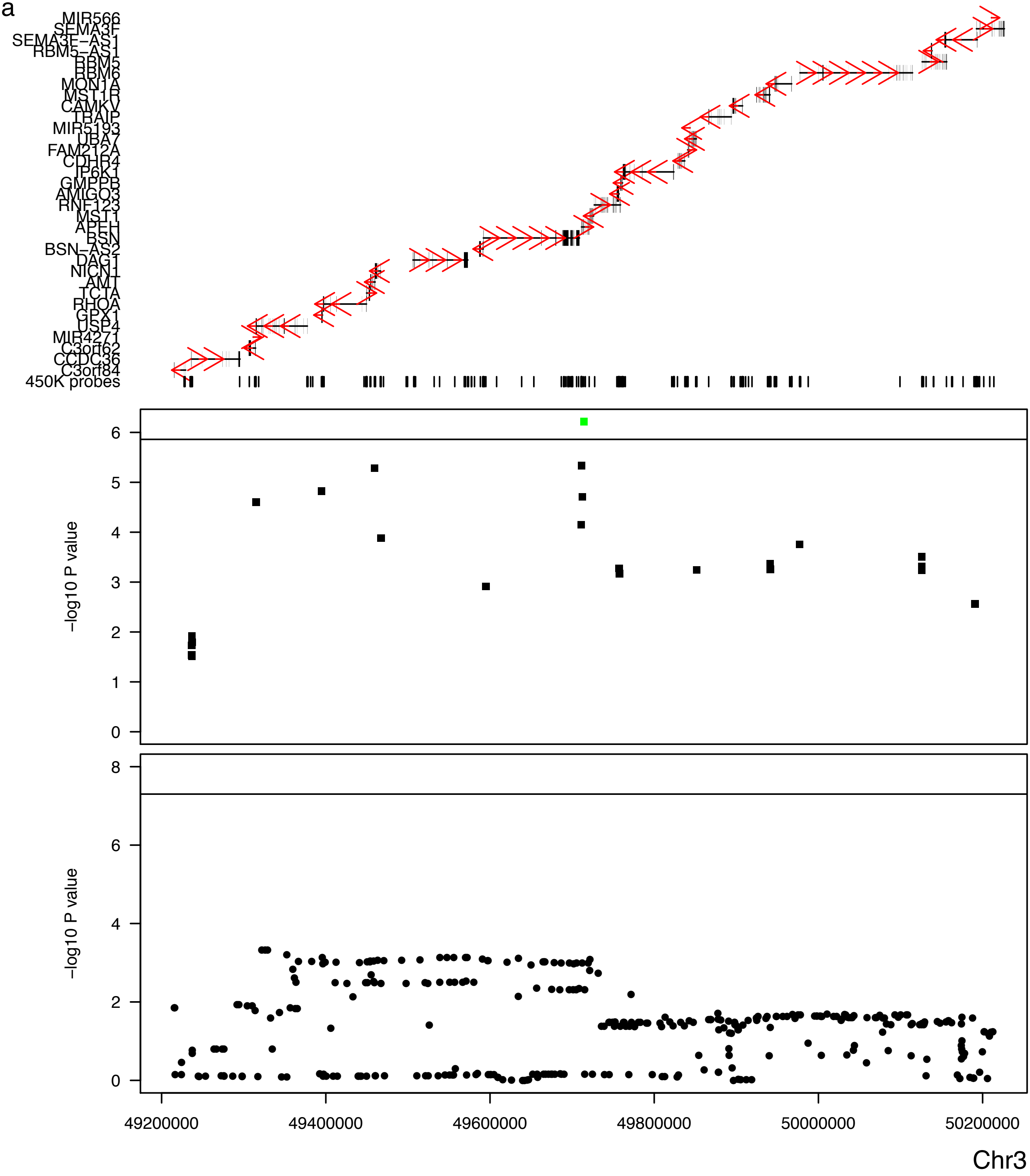
MIR566
SEMA3F
SEMA3F-AS1
RBM5-AS1
RBM5
RBM6
MON1A
MST1R
CAMKV
TRAIP
MIR5193
UBA7
FAM212A
CDHR4
IP6K1
GMPPB
AMIGO3
RNF123
MST1
APEH
BSN
BSN-AS2
DAG1
NICN1
AMT
TCTA
RHOA
GPX1
USP4
MIR4271
C3orf62
CCDC36
C3orf84
450K probes
-log10 P value
-log10 P value
49200000
49400000
49600000
49800000
50000000
50200000
Chr3

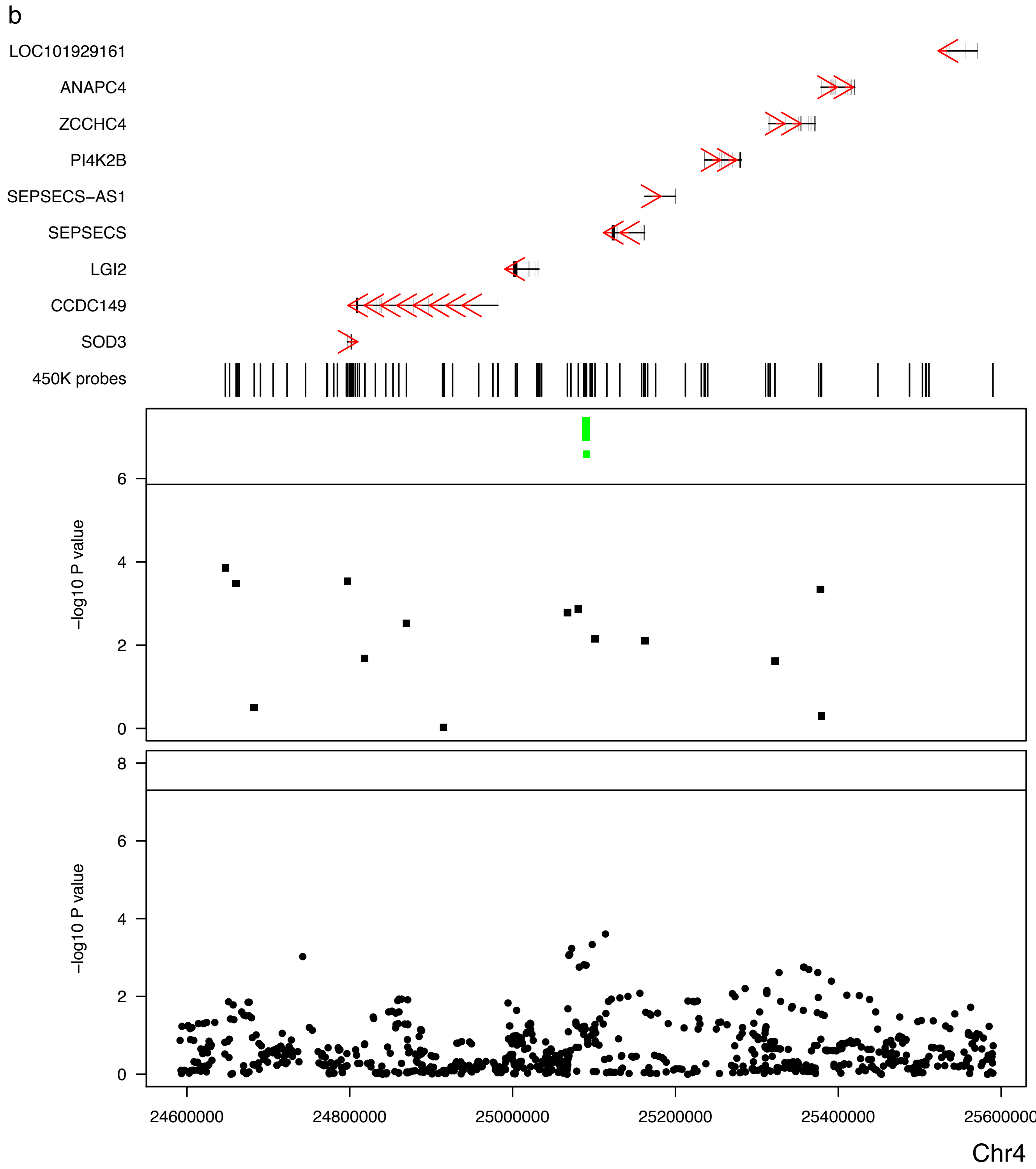

b
LOC101929161
ANAPC4
ZCCHC4
PI4K2B
SEPSECS−AS1
SEPSECS
LGI2
CCDC149
SOD3
450K probes
−log10 P value
0
2
4
6
−log10 P value
0
2
4
6
8
24600000
24800000
25000000
25200000
25400000
25600000
Chr4

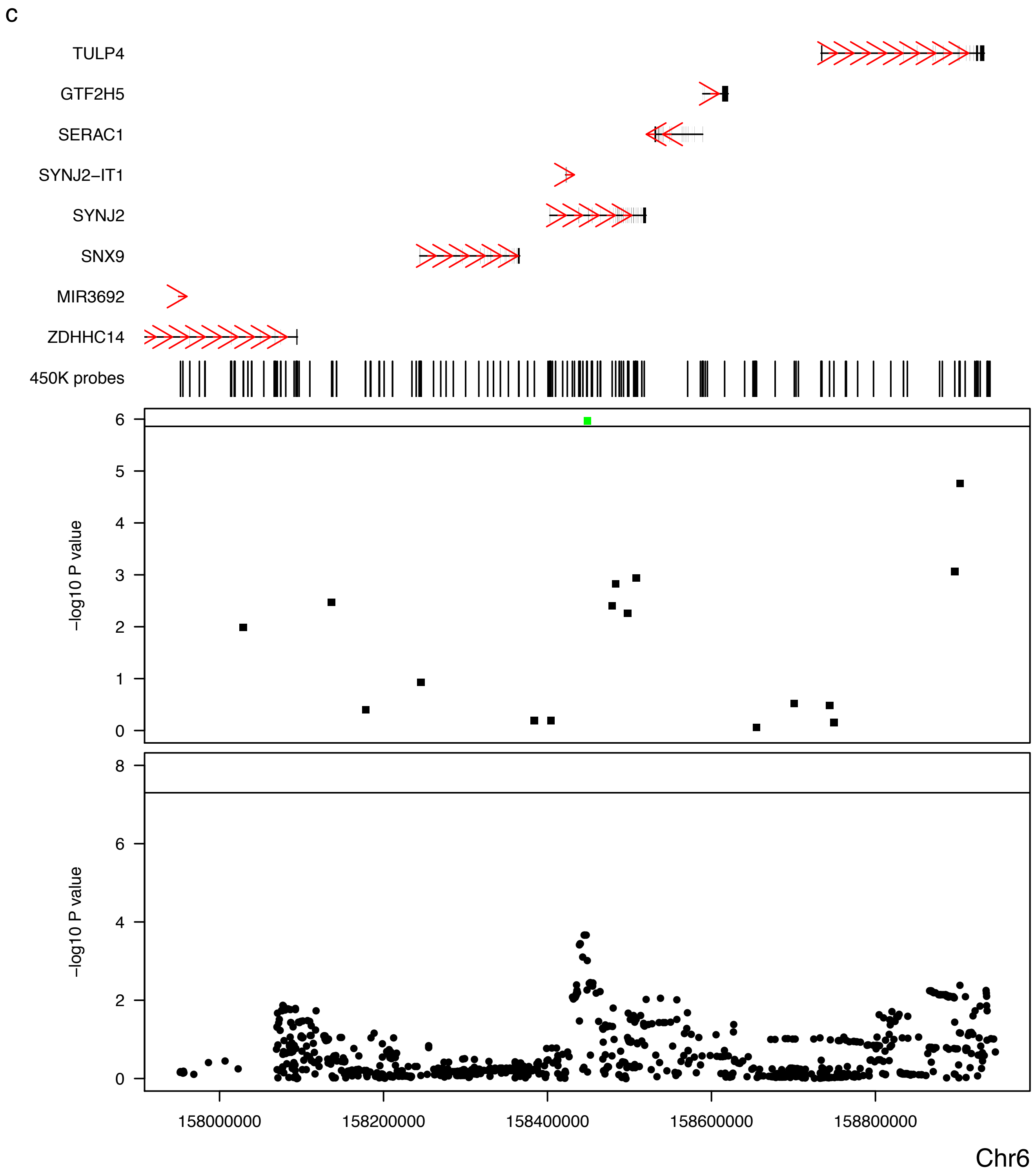
c
TULP4
GTF2H5
SERAC1
SYNJ2−IT1
SYNJ2
SNX9
MIR3692
ZDHHC14
450K probes
−log10 P value
−log10 P value
158000000
158200000
158400000
158600000
158800000
Chr6

d

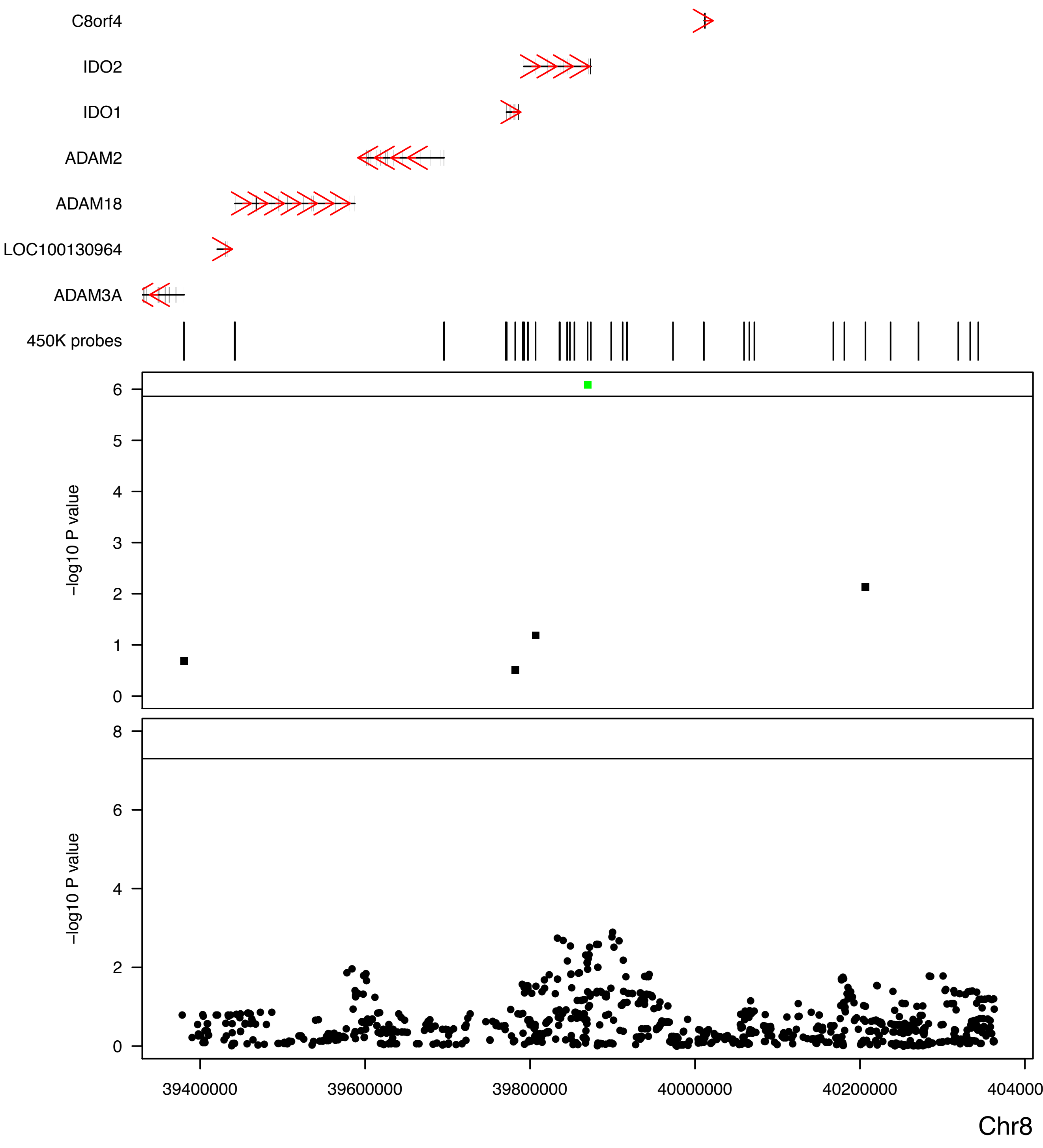

C8orf4
IDO2
IDO1
ADAM2
ADAM18
LOC100130964
ADAM3A
450K probes
−log10 P value
0
1
2
3
4
5
6
−log10 P value
0
2
4
6
8
39400000
39600000
39800000
40000000
40200000
404000
Chr8

e

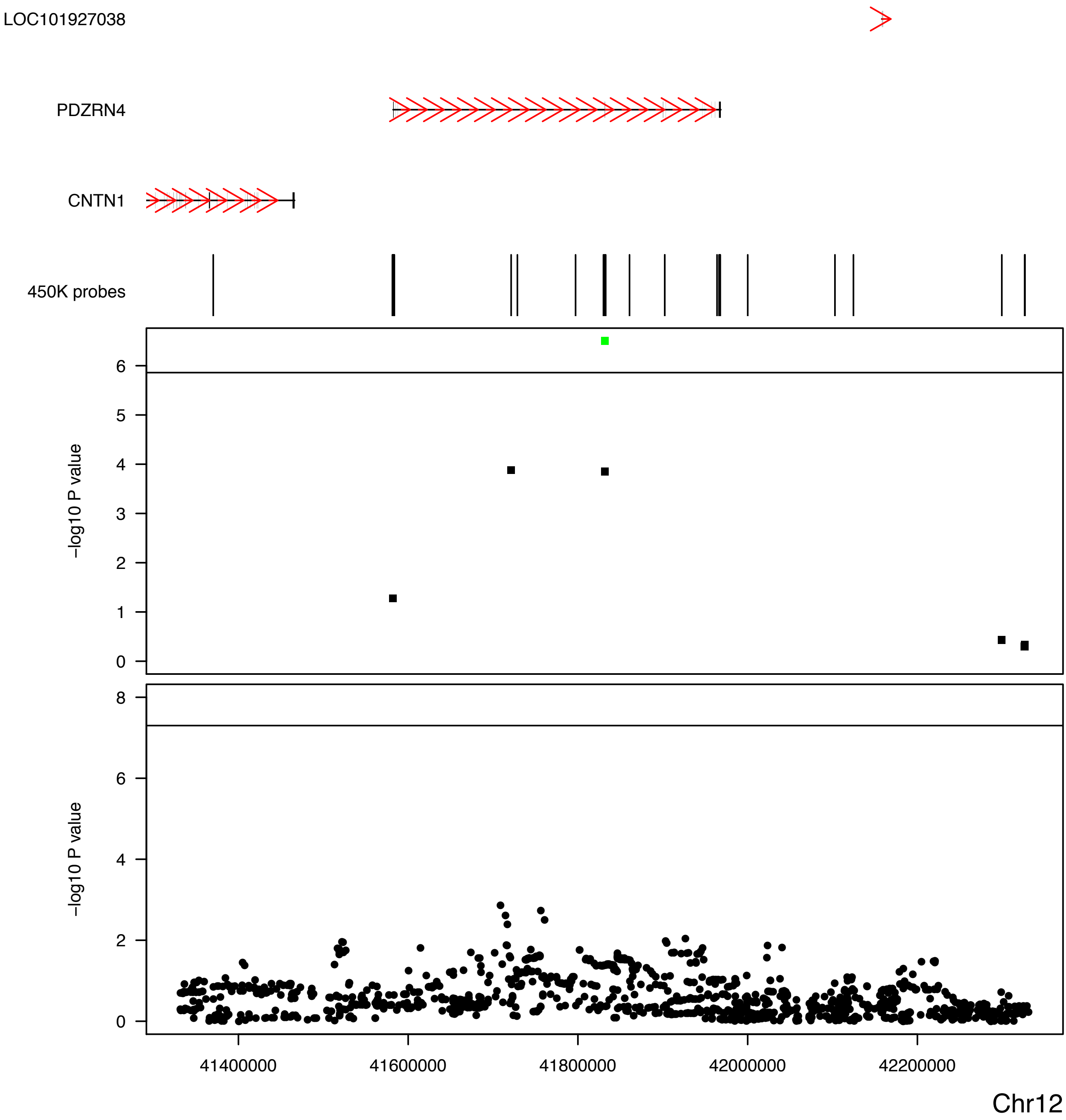

LOC101927038
PDZRN4
CNTN1
450K probes
-log10 P value
6
5
4
3
2
1
0
-log10 P value
8
6
4
2
0
41400000
41600000
41800000
42000000
42200000
Chr12

f

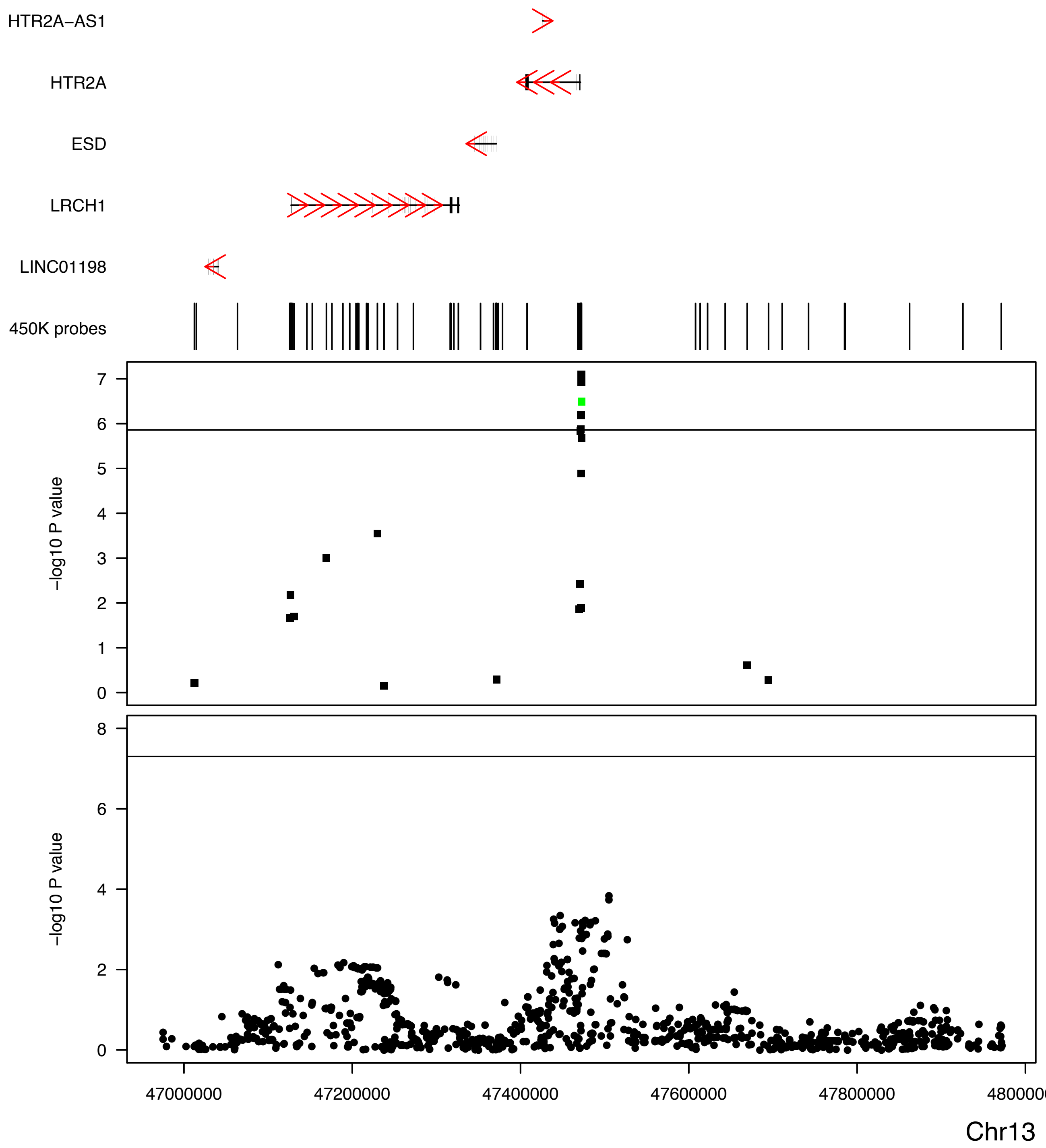

HTR2A−AS1
HTR2A
ESD
LRCH1
LINC01198
450K probes
−log10 P value
0
1
2
3
4
5
6
7
−log10 P value
0
2
4
6
8
47000000
47200000
47400000
47600000
47800000
Chr13

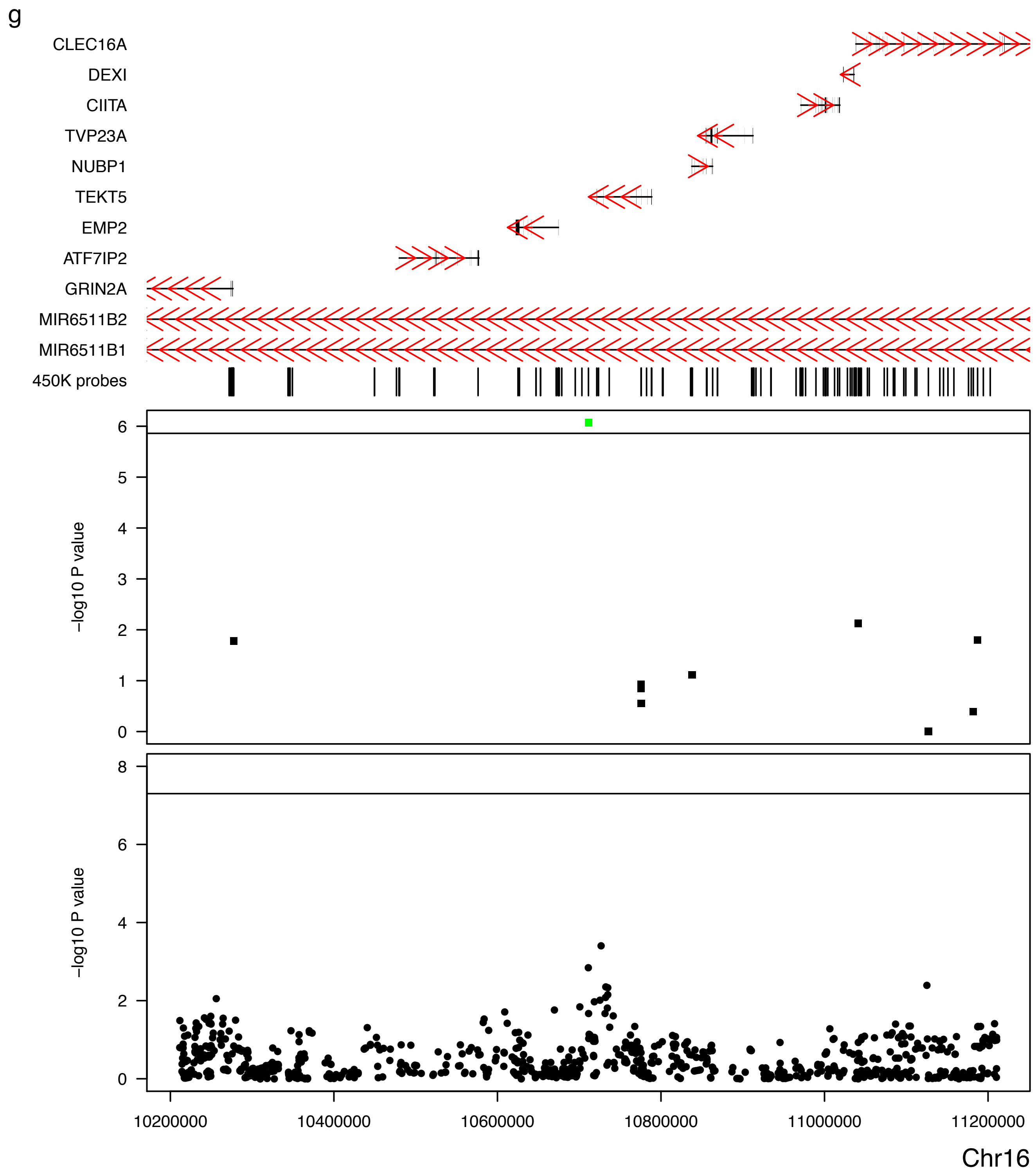

g
CLEC16A
DEXI
CIITA
TVP23A
NUBP1
TEKT5
EMP2
ATF7IP2
GRIN2A
MIR6511B2
MIR6511B1
450K probes
−log10 P value
−log10 P value
10200000
10400000
10600000
10800000
11000000
11200000
Chr16

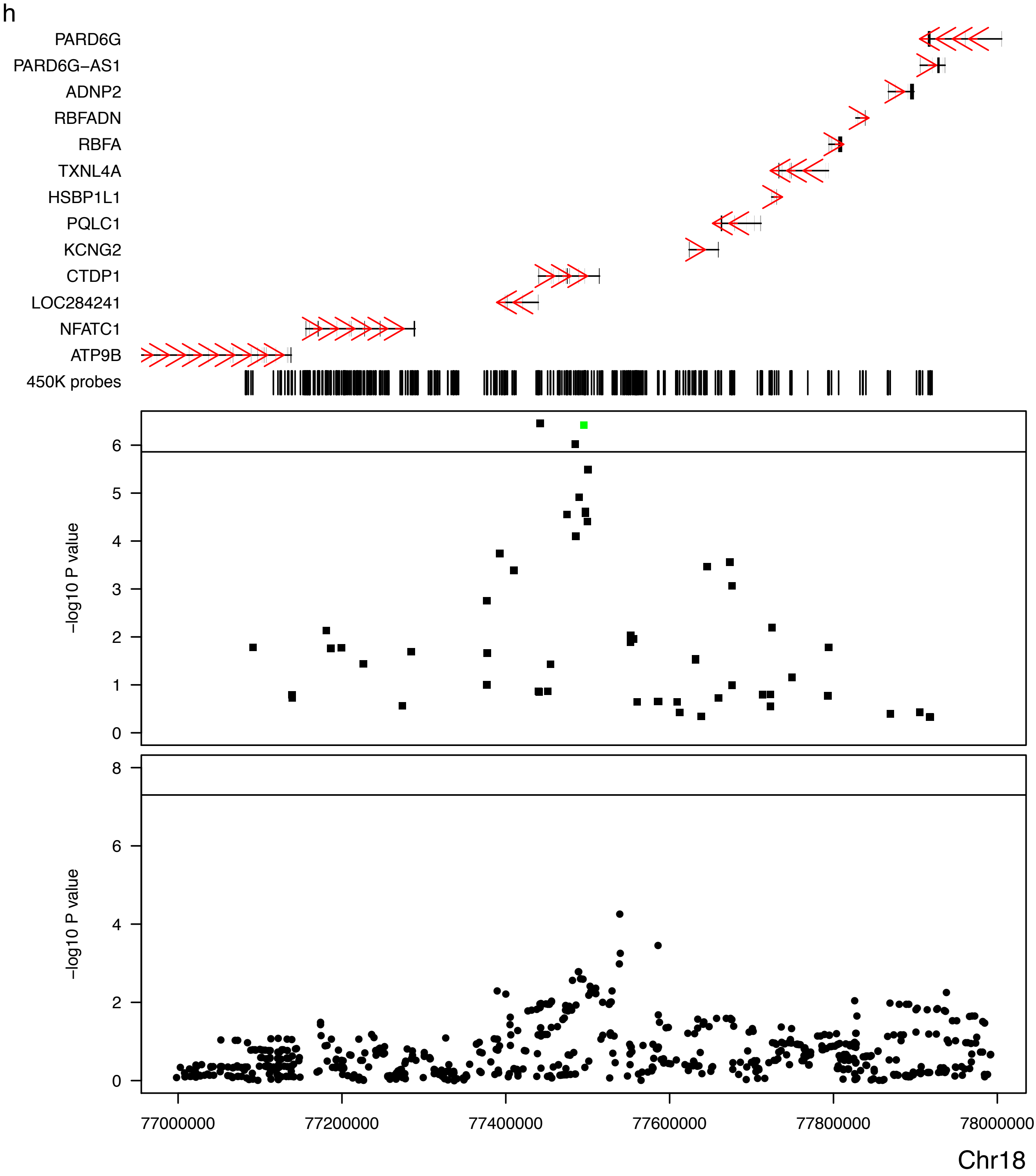

h
PARD6G
PARD6G−AS1
ADNP2
RBFADN
RBFA
TXNL4A
HSBP1L1
PQLC1
KCNG2
CTDP1
LOC284241
NFATC1
ATP9B
450K probes
−log10 P value
0
1
2
3
4
5
6
−log10 P value
0
2
4
6
8
77000000
77200000
77400000
77600000
77800000
78000000
Chr18

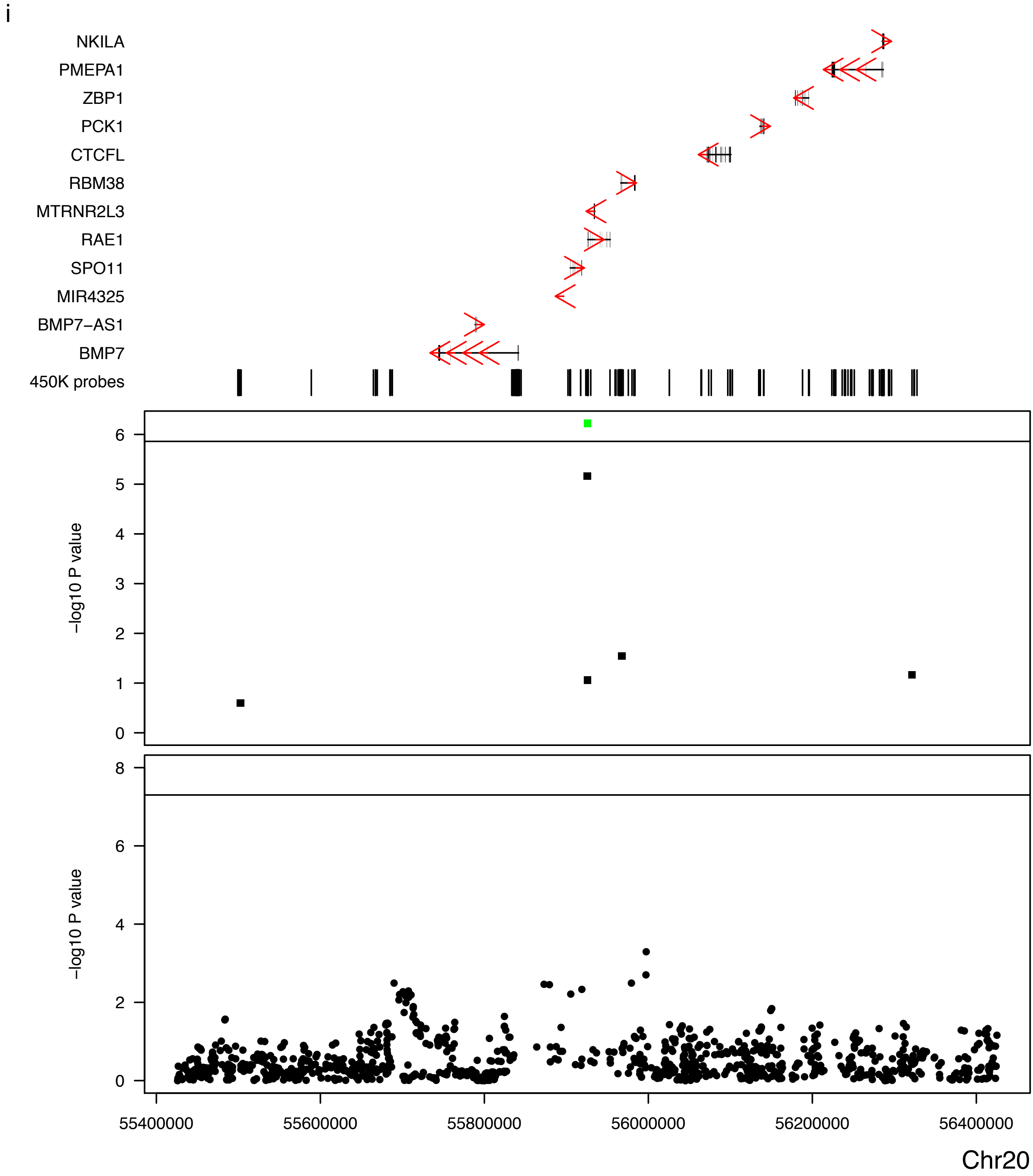

i
NKILA
PMEPA1
ZBP1
PCK1
CTCFL
RBM38
MTRNR2L3
RAE1
SPO11
MIR4325
BMP7−AS1
BMP7
450K probes
−log10 P value
0
1
2
3
4
5
6
−log10 P value
0
2
4
6
8
55400000
55600000
55800000
56000000
56200000
56400000
Chr20

Figure S4: Novel loci identified using SMR analysis for puberty (Tanner stage). Shown are examples where the SMR analysis identified a significant association between DNA methylation at **a**) cg06313718, **b**) cg06759629, cg01218619, cg19312667, cg02428792, cg26262055, and cg17688837, **c**) cg05987787, **d**) cg03595199, **e**) cg15417244, **f**) cg07075299, **g**) cg27409771, **h**) cg25357022, **i**) cg19836589 and Tanner stage. In each example, there is no association reported in the GWAS analysis within 0.5 Mb[2]. For each example, there is a gene track along the top and zoomed in Manhattan plots of the SMR analysis *P*-values (middle panel) and GWAS *P*-values (bottom panel). The black solid horizontal lines, indicate the multiple testing threshold for the SMR analysis ($P < 1.38 \times 10^{-6}$) and GWAS ($P < 5 \times 10^{-8}$); in the SMR Manhattan plots the green points indicate those characterized by a non-significant heterogeneity test ($P > 0.05$).

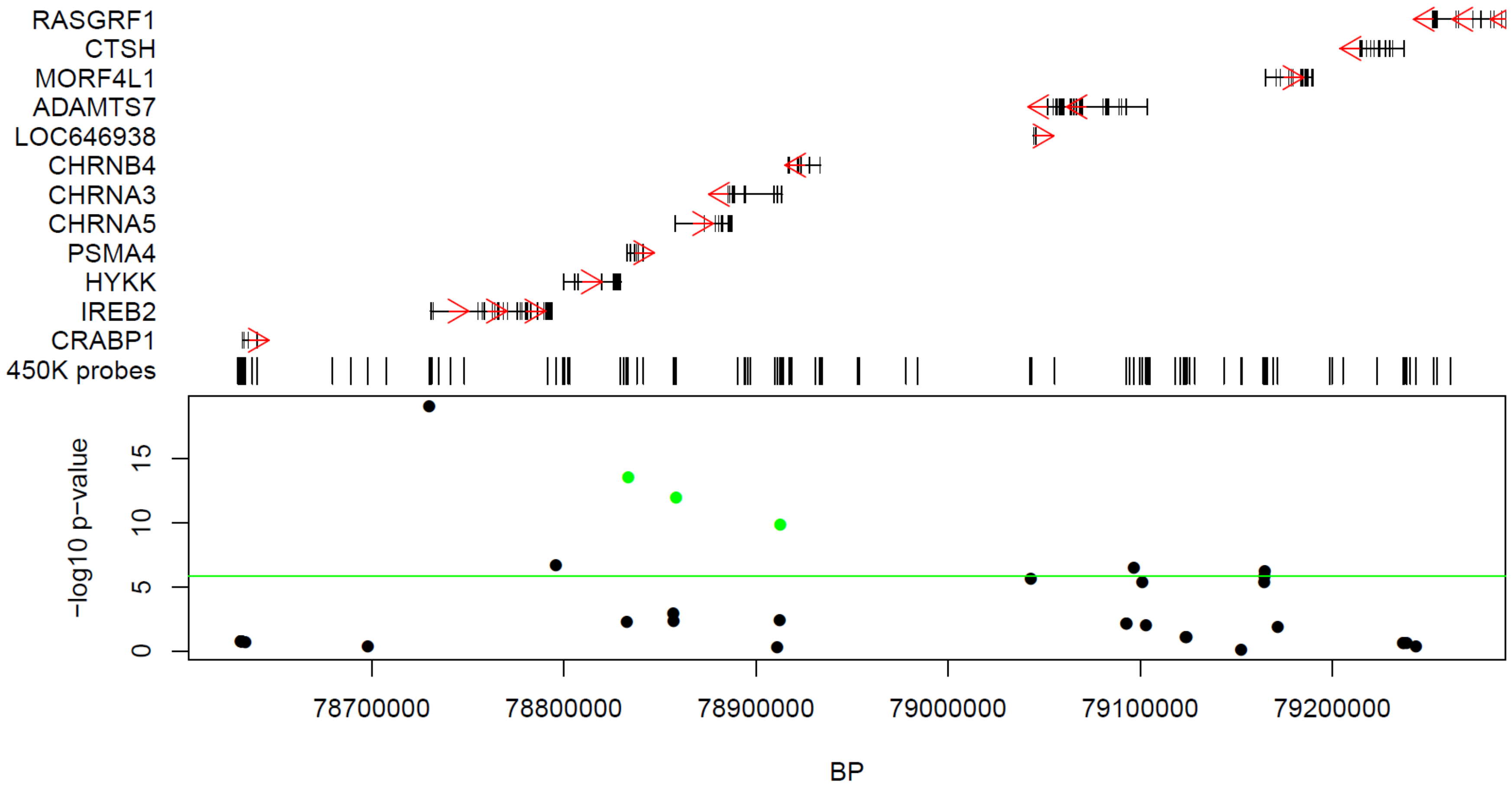

Figure S5: SMR analysis across a region on chromosome 15 identified by a GWAS analysis of cigerettes smoked per day. Shown on the y-axis is the SMR test –log10 *P*-value. Each point represents a SMR test for an individual DNA methylation site. The green horizontal line indicates the significance threshold ($P < 1.42 \times 10^{-6}$); green points highlight the significant SMR tests which did not show significant heterogeneity ($P > 0.05$); i.e. the genetic associations indicate a pleiotropic relationship between the complex trait and DNA methylation.

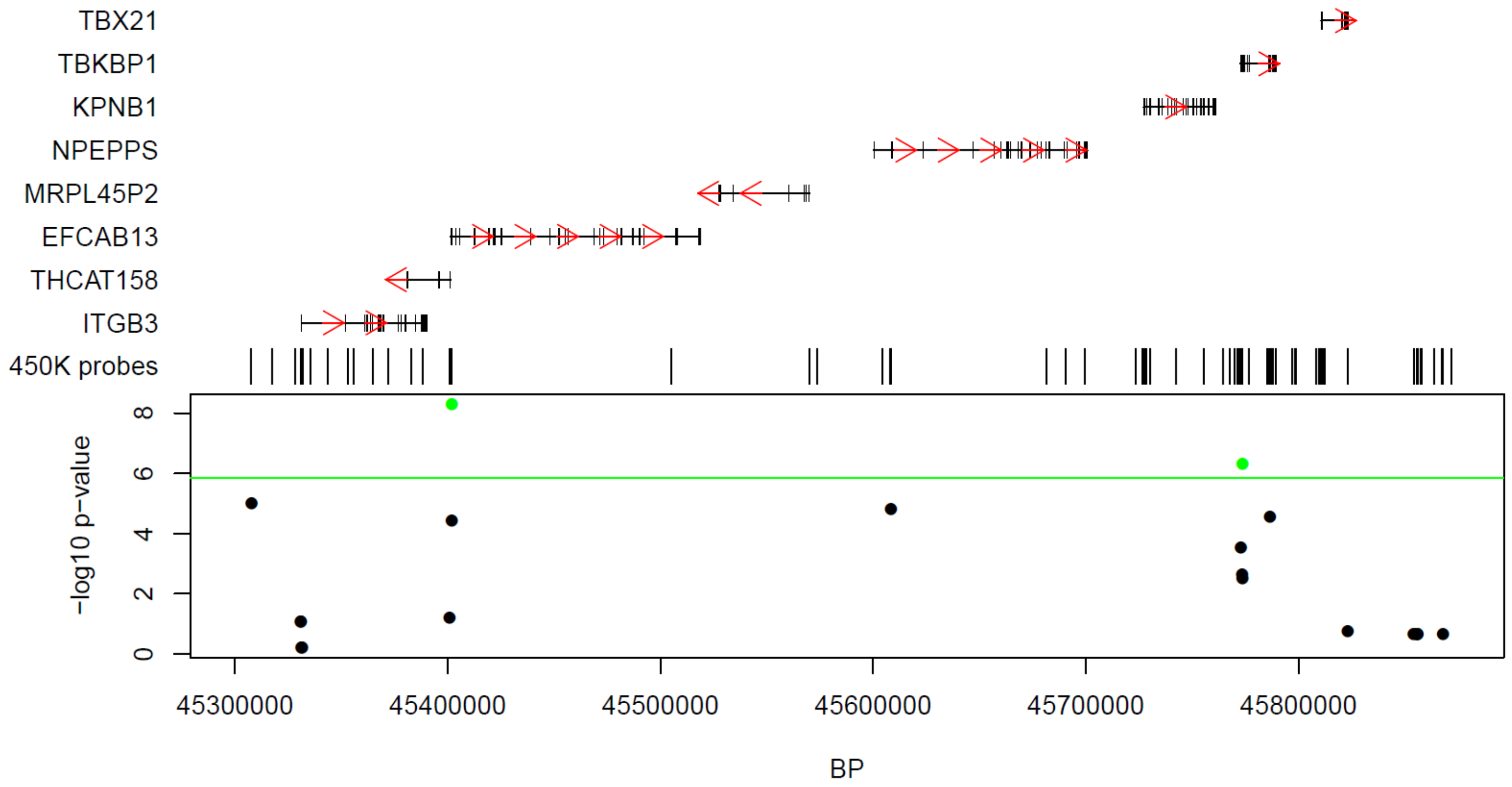

Figure S6: SMR analysis across a region on chromosome 17 identified by a GWAS analysis of total cholestrol. Shown on the y-axis is the SMR test –log10 *P*-value. Each point represents a SMR test for an individual DNA methylation site. The green horizontal line indicates the significance threshold ($P < 1.42 \times 10^{-6}$); green points highlight the significant SMR tests which did not show significant heterogeneity ($P > 0.05$); i.e. the genetic associations indicate a pleiotropic relationship between the complex trait and DNA methylation.

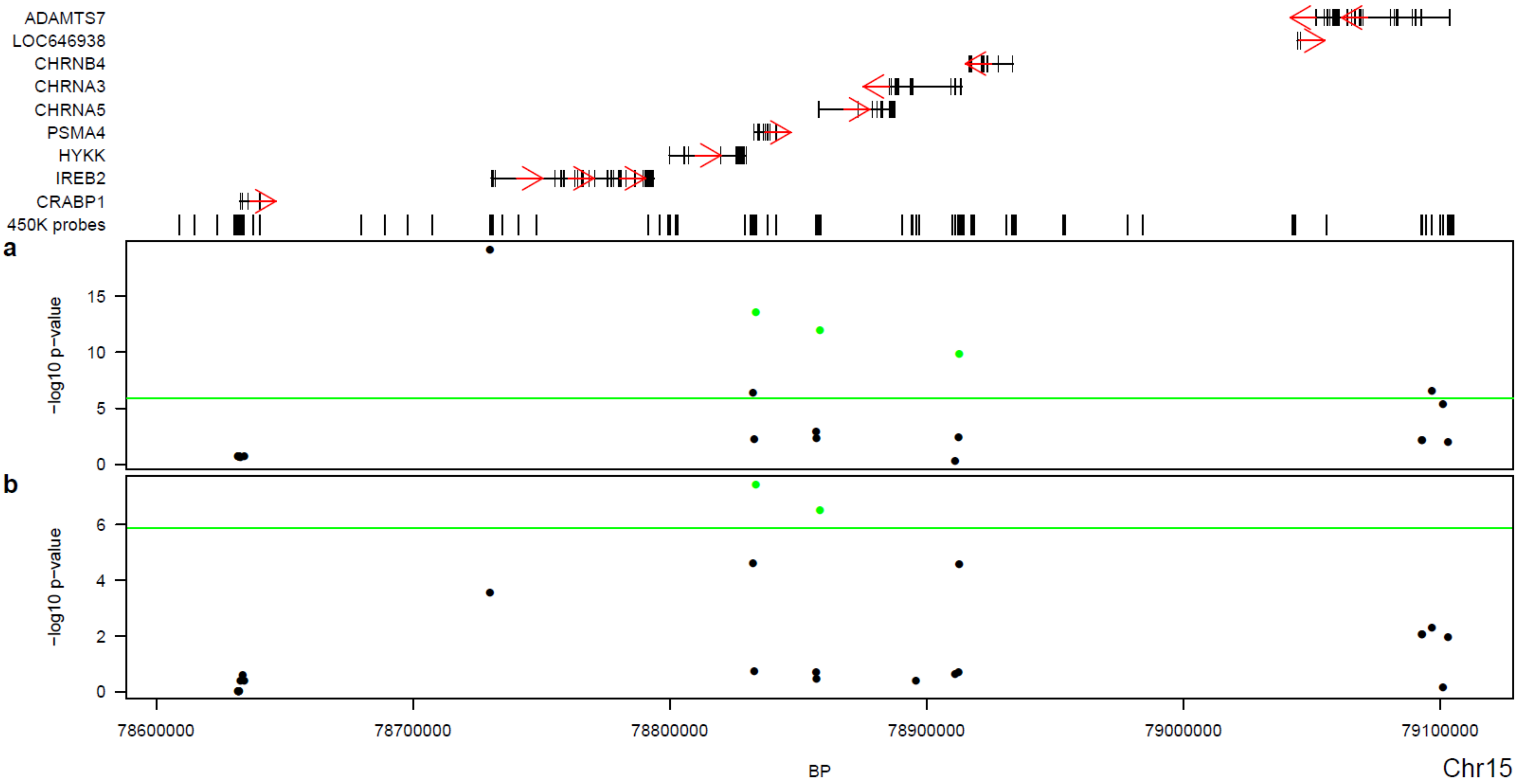

Figure S7: DNA methylation at CpG sites annotated to *CHRNA5* are associated with both schizophrenia and cigerettes smoked per day. Manhattan plots of SMR analysis across the *CHRNA5* locus where DNA methylation at cg24631222 and cg04140906 is associated with both **a**) schizophrenia[3] and **b**) cigarettes per day[4]. Shown on the y-axis is the SMR test –log10 *P*-value. Each point represents an SMR test for an individual DNA methylation site. The green horizontal line indicates the significance threshold ($P < 1.42 \times 10^{-6}$); green points highlight the significant SMR tests which were not characterized by significant heterogeneity ($P > 0.05$); i.e. the genetic associations indicate a pleiotropic relationship between the complex trait and DNA methylation.

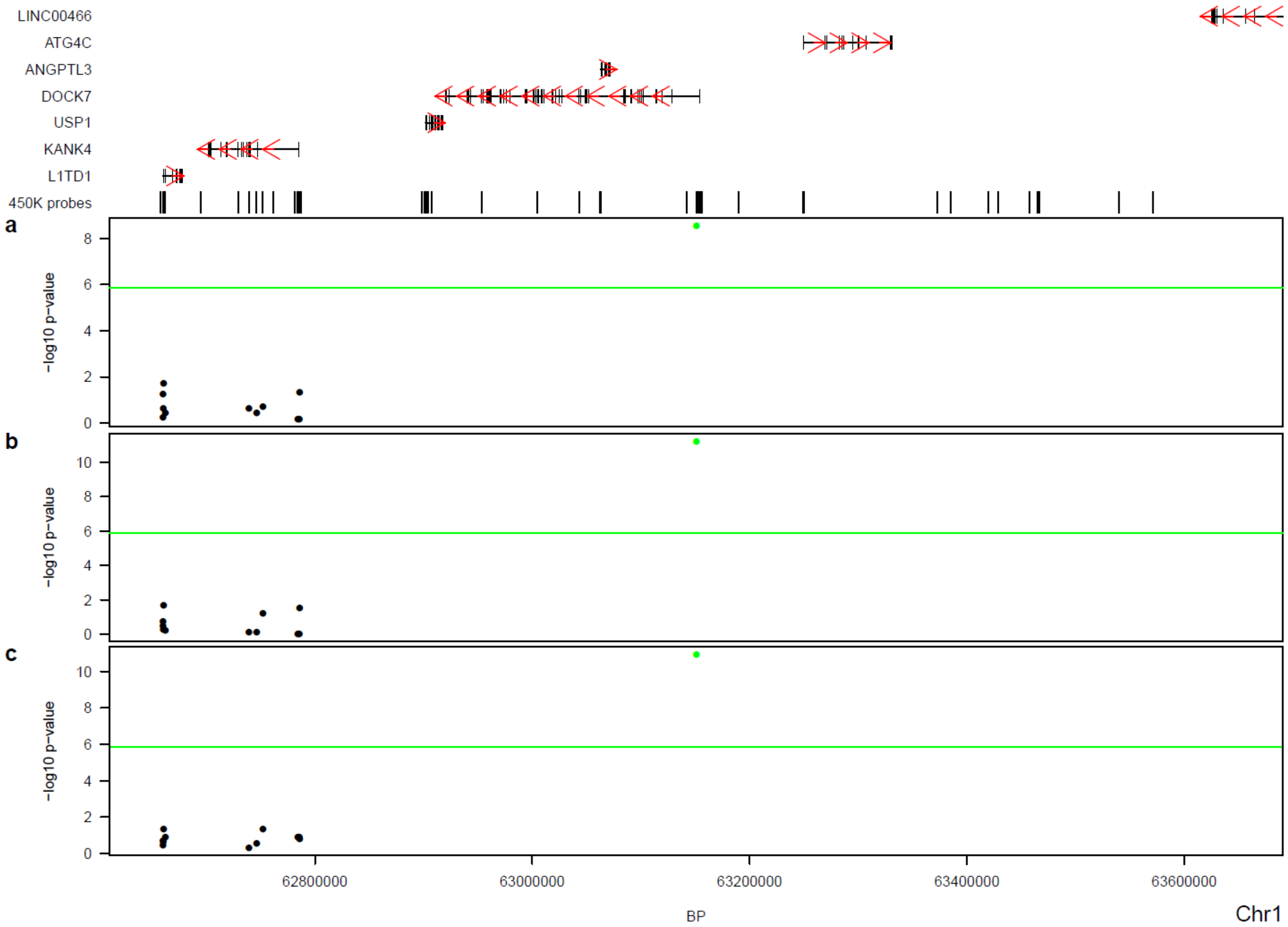

Figure S8: DNA methylation at *DOCK7* is associated with LDL, total cholesterol and triglycerides. Manhattan plots of SMR analysis across the *DOCK7* locus where DNA methylation at cg10583485 is associated with both **a**) LDL, **b**) total cholesterol and **c**) triglycerides[5]. Shown on the y-axis is the SMR test –log10 *P*-value. Each point represents an SMR test for an individual DNA methylation site. The green horizontal line indicates the significance threshold ($P < 1.42 \times 10^{-6}$); green points highlight the significant SMR tests which were not characterized by significant heterogeneity ($P > 0.05$); i.e. the genetic associations indicate a pleiotropic relationship between the complex trait and DNA methylation.

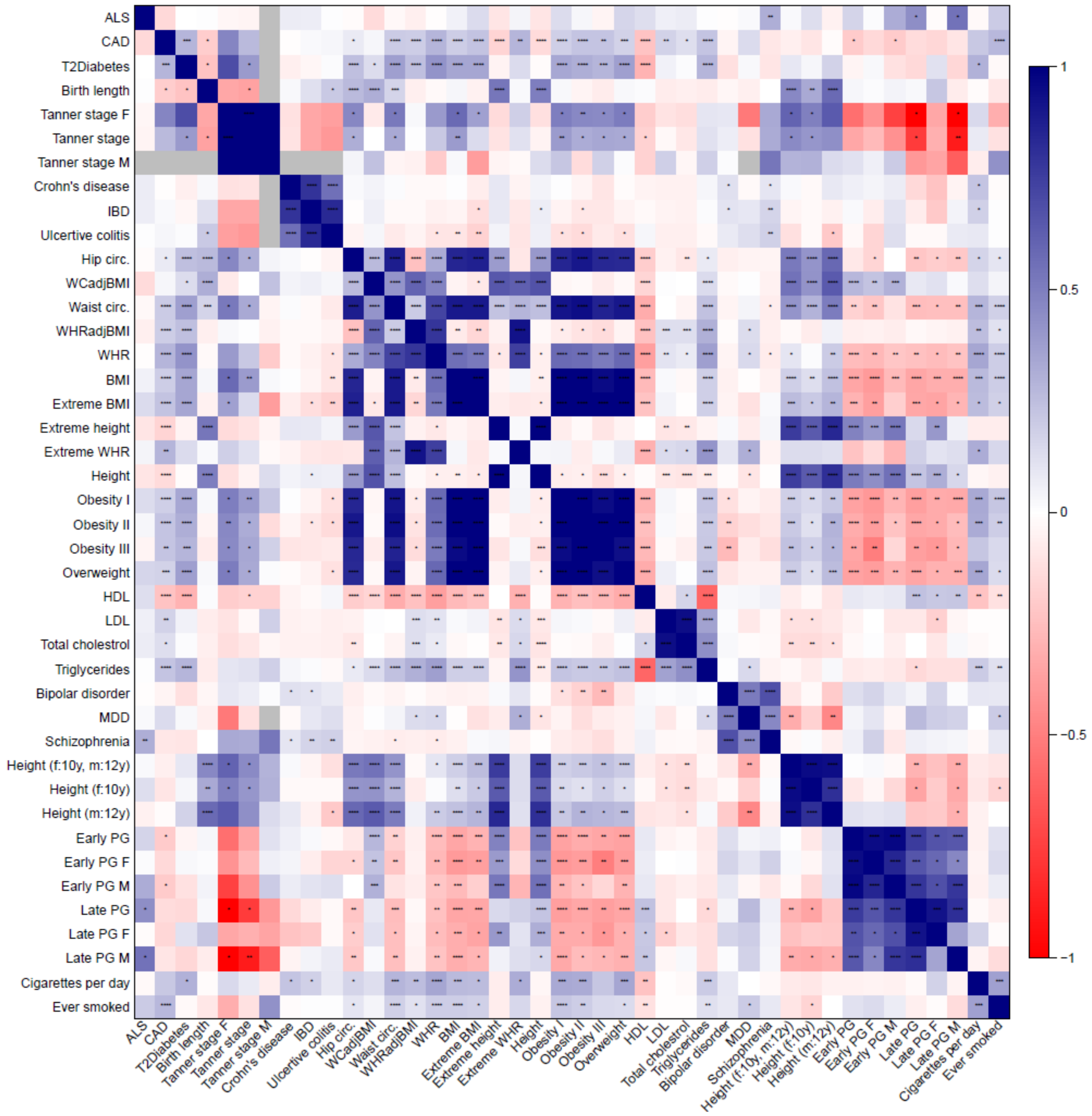

Figure S9: **A heat map of genetic correlations between complex traits considered for the SMR analysis.** The colour of each square represents the magnitude of the correlation between two traits, while asterisk indicate the significance of a non-zero correlation where * P < 0.05; ** P< 0.005; *** P < 0.0005; *** P < 0.00005. Genetic correlations were calculated using LDScore regression[6]. F-female; M-male; y – years; PG – pubertal growth; ALS – Amyloid lateral sclerosis; BMI – body mass index; CAD- coronary artery disease; WHR – waist hip ratio; IBD – inflammatory bowel disease; HDL/LDL – high/low density lipoprotein; MDD – major depressive disorder.

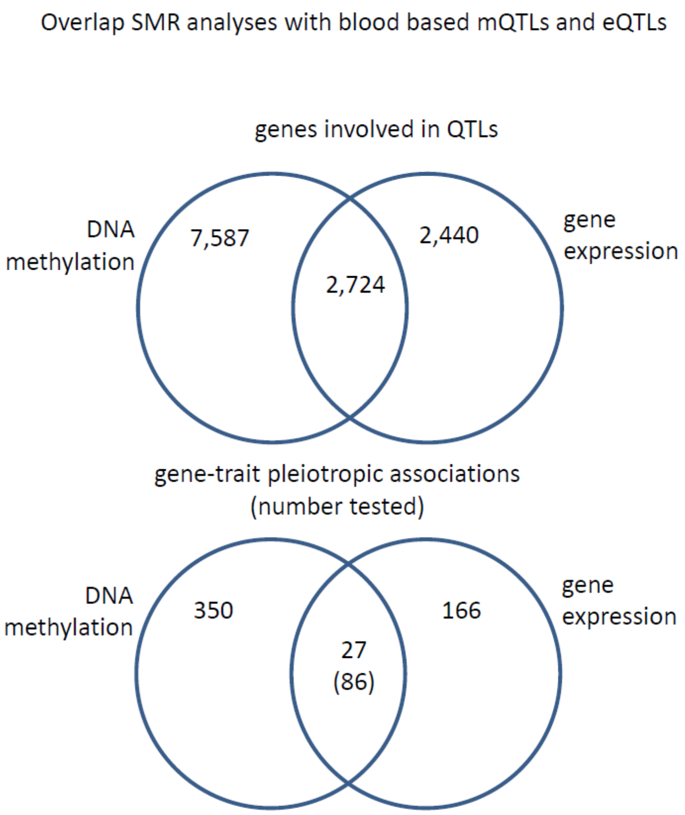

Figure S10: Flow diagram depicting the overlap of SMR analysis results obtained using blood mQTLs and blood eQTLs.

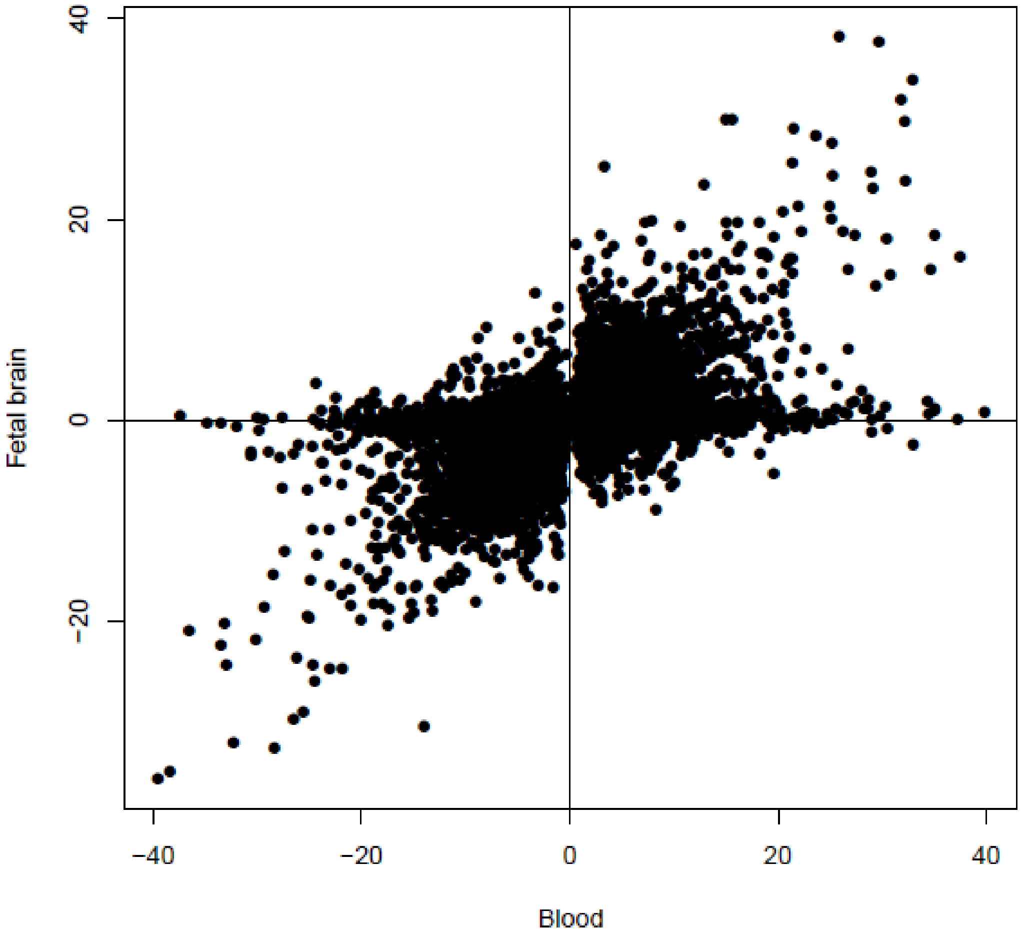

Figure S11: Scatterplot of effect sizes for mQTL identified in blood and tested fetal brain. All blood mQTL ($P < 1x\text{-}10^{-10}$) included in the SMR analysis were tested in fetal brain samples. Each point represents a SNP-DNA methylation site pairing, with the difference in DNA methylation per allele (%) shown for blood (x-axis) and fetal brain (y-axis).

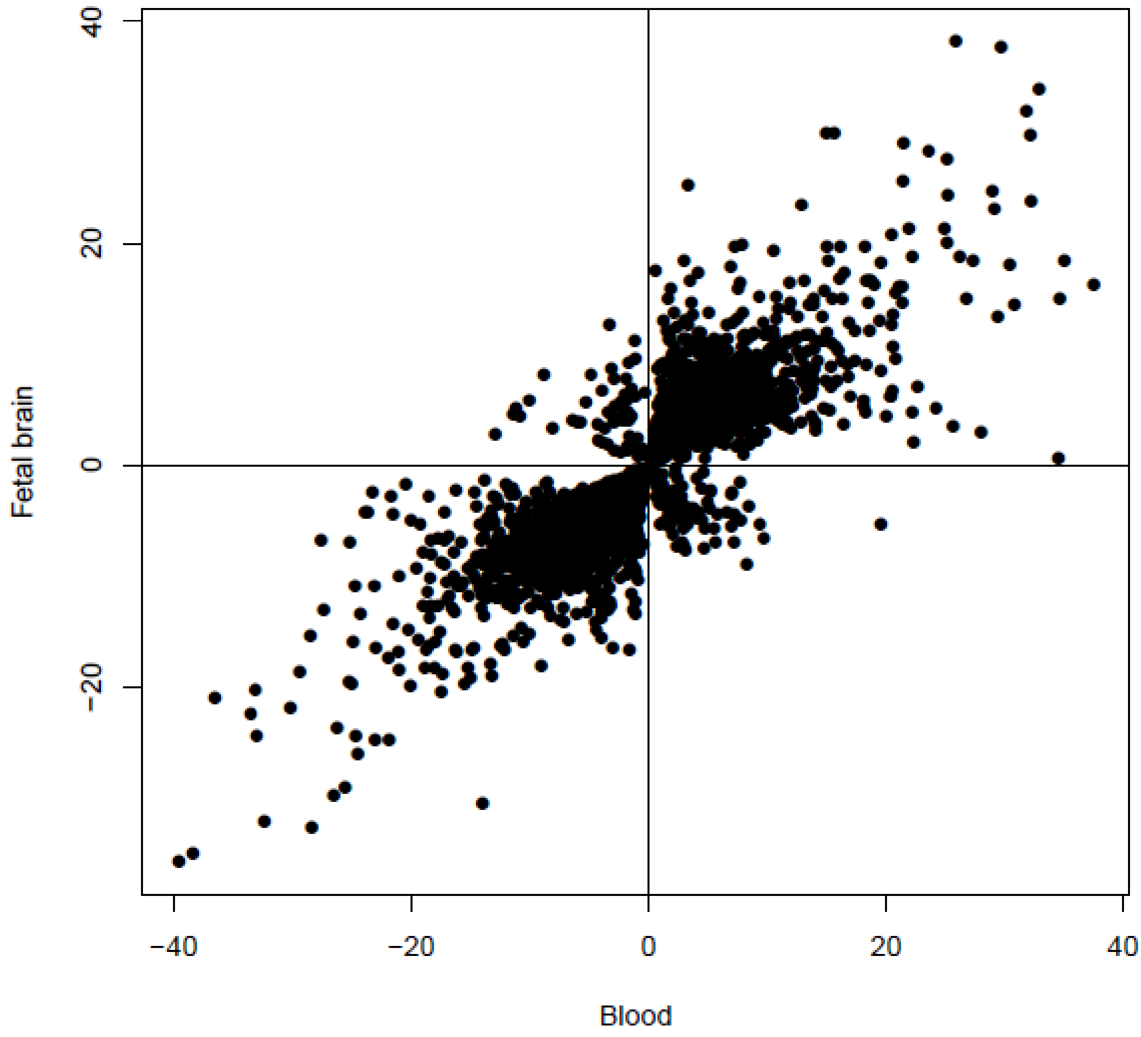

Figure S12: Scatterplot of effect sizes for mQTL identified and included in the SMR analysis for both blood and fetal brain. Each point represents a SNP-DNA methylation site pairing, with the difference in DNA methylation per allele (%) shown for blood (x-axis) and fetal brain (y-axis).

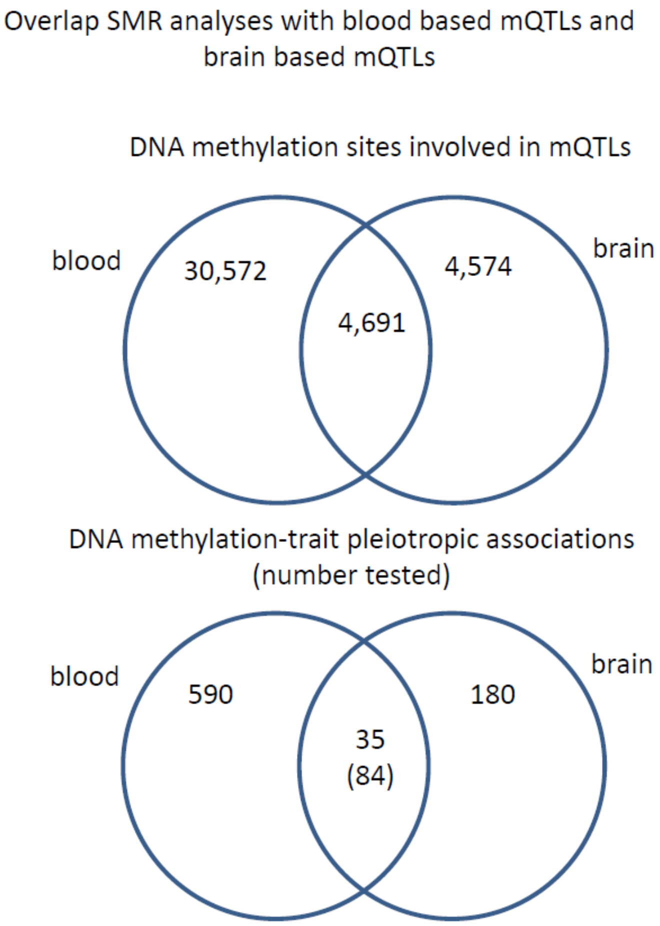

Figure S13: Flow diagram demonstrating the overlap of EWAS associations identified with SMR analysis using blood and fetal brain mQTLs.

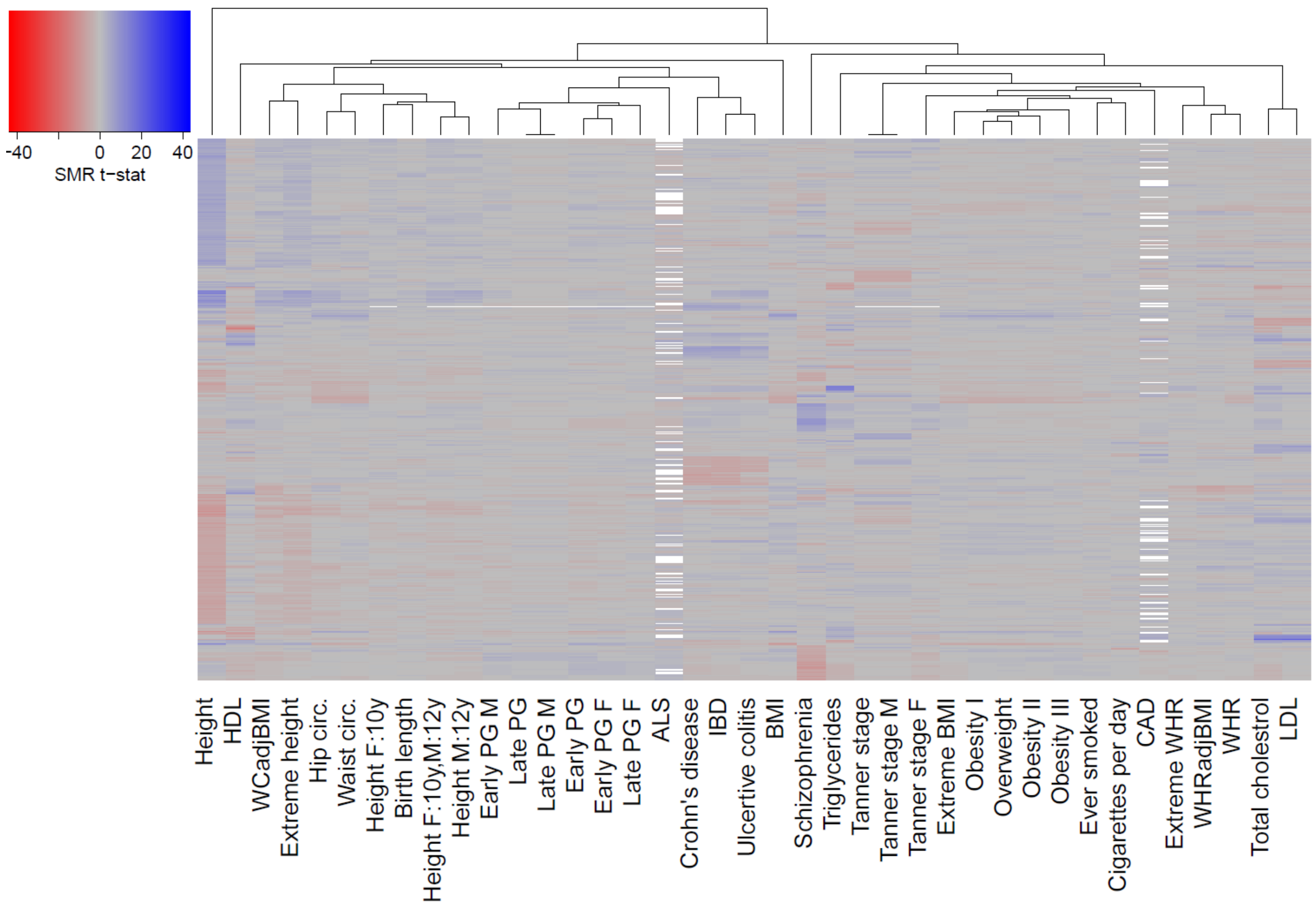

Figure S14: Heat-map of SMR probe associations across 38 GWAS traits. Shown is the t-statistic (b_SMR/se_SMR) of the GWAS trait (columns) for an individual DNA methylation site (row). Only traits (n = 38) tested against at least 20,000 DNA methylation sites were included in this analysis for DNA methylation sites (n = 1,044) associated with at least one phenotype. SMR- summarized mendelian randomization; WCadjBMI – waist circumference adjusted for body mass index; WHRadjBMI – waist hip ratio adjusted for body mass index; circ. – circumference; F-female; M-male; y – years; PG – pubertal growth; BMI – body mass index; CAD- coronary artery disease; WHR – waist hip ratio; IBD – inflammatory bowel disease; HDL/LDL – high/low density lipoprotein.

References

1. Hannon, E. *et al*. An integrated genetic-epigenetic analysis of schizophrenia: evidence for co-localization of genetic associations and differential DNA methylation. *Genome Biol* **17**, 176 (2016).
2. Cousminer, D.L. *et al*. Genome-wide association study of sexual maturation in males and females highlights a role for body mass and menarche loci in male puberty. *Hum Mol Genet* **23**, 4452-64 (2014).
3. Schizophrenia Working Group of the PGC *et al*. Biological insights from 108 schizophrenia-associated genetic loci. *Nature* **511**, 421-+ (2014).
4. Tobacco and Genetics Consortium. Genome-wide meta-analyses identify multiple loci associated with smoking behavior. *Nat Genet* **42**, 441-7 (2010).
5. Global Lipids Genetics Consortium *et al*. Discovery and refinement of loci associated with lipid levels. *Nat Genet* **45**, 1274-83 (2013).
6. Bulik-Sullivan, B. *et al*. An atlas of genetic correlations across human diseases and traits. *Nat Genet* **47**, 1236-41 (2015).
