# Supplementary material for: Pleiotropic Effects of Trait-Associated Genetic Variation on DNA Methylation: Utility for Refining GWAS Loci
Source: Am J Hum Genet. 2017 May 18;100(6):954–9. doi: 10.1016/j.ajhg.2017.04.013 (PMC5473725; doi:10.1016/j.ajhg.2017.04.013)
Supplement: Document S2. Article plus Supplemental Data [file mmc8.pdf]

REPORT

Pleiotropic Effects of Trait-Associated Genetic Variation on DNA Methylation: Utility for Refining GWAS Loci

Eilis Hannon,[1] Mike Weedon,[1] Nicholas Bray,[2] Michael O'Donovan,[2] and Jonathan Mill[1,*]

Most genetic variants identified in genome-wide association studies (GWASs) of complex traits are thought to act by affecting gene regulation rather than directly altering the protein product. As a consequence, the actual genes involved in disease are not necessarily the most proximal to the associated variants. By integrating data from GWAS analyses with those from genetic studies of regulatory variation, it is possible to identify variants pleiotropically associated with both a complex trait and measures of gene regulation. In this study, we used summary-data-based Mendelian randomization (SMR), a method developed to identify variants pleiotropically associated with both complex traits and gene expression, to identify variants associated with complex traits and DNA methylation. We used large DNA methylation quantitative trait locus (mQTL) datasets generated from two different tissues (blood and fetal brain) to prioritize genes for >40 complex traits with robust GWAS data and found considerable overlap with the results of SMR analyses performed with expression QTL (eQTL) data. We identified multiple examples of variable DNA methylation associated with GWAS variants for a range of complex traits, demonstrating the utility of this approach for refining genetic association signals.

There has been major progress in the identification of genetic variants influencing a diverse range of complex human phenotypes, including anthropometric measures (e.g., height and weight),[1,2] cardiovascular disease,[3,4] inflammatory disorders,[5] neurological diseases,[6,7] and psychiatric illness.[8–10] The challenge is now to improve our understanding of the biological effects of these genetic risk factors, especially because the actual genes involved in mediating phenotypic variation are not necessarily the most proximal to the lead SNPs identified in genome-wide association studies (GWASs). Supported by the observation that GWAS variants are preferentially located in enhancers and regions of open chromatin,[11,12] the majority of common genetic risk factors are predicted to influence gene regulation rather than directly affect the coding sequences of transcribed proteins.[13]

Expression quantitative trait loci (eQTLs) have been successfully used for investigating the functional consequences of GWAS variants.[14,15] The co-localization of GWAS and eQTL variants, however, is not sufficient to show that the overlapping association signals are causally related, given that the association signals might be tagging different causal variants in the same linkage disequilibrium (LD) block. Recently, an approach called summary-data-based Mendelian randomization (SMR) was proposed as a strategy for identifying overlapping genetic signals associated with both phenotypic and transcriptional variation and subsequently distinguishing pleiotropic effects (i.e., where the same variant influences both outcomes, although not necessarily dependently) from those that are artifacts of LD.[16] Genetic effects on gene expression can be mediated by epigenetic processes such as changes in DNA methylation, a cytosine modification that has an essential role in mammalian development.[17] We have previously demonstrated the utility of DNA methylation QTLs (mQTLs) for interpreting GWAS findings by identifying specific examples where genetic polymorphisms associated with schizophrenia (MIM: 181500) co-localize with variants associated with DNA methylation.[18,19] In this study, we applied the SMR approach to test 35,263 DNA methylation sites against 43 complex phenotypes with robust GWAS data (Table S1) by using mQTLs identified in our recent analysis of methylomic variation in whole blood and imputed SNP genotypes ($n = 639$; mQTL $p < 1 \times 10^{-10}$; a full description of this dataset, referred to as phase 1, can be found here[19]) in conjunction with publicly available summary data from a series of well-powered GWAS analyses.

The first stage of the SMR analysis identifies the most significantly associated SNP for a DNA methylation site (that is also present in the GWAS dataset) as an instrumental variable for testing for an association with a phenotype by the two-step least-squares (2SLS) approach, which uses the same SNP to compare the mQTL coefficients with those from a GWAS of the phenotype (Figure S1A). This approach identified 1,932 associations ($p < 1.42 \times 10^{-6}$ corrected for 35,263 DNA methylation sites) between 31 complex traits and 1,354 individual DNA methylation sites (Table S2 and Figure S2). Because these associations can be driven by two highly correlated but different causal variants for the GWAS trait and DNA methylation, the second stage of the SMR approach repeats the analysis with alternative SNPs associated with DNA methylation as the instrument and performs a HEIDI (heterogeneity in dependent instruments) test for heterogeneity in the resulting association statistics. If a single causal variant is associated with both

[1]University of Exeter Medical School, Exeter EX2 5DW, UK; [2]MRC Centre for Neuropsychiatric Genetics and Genomics, Cardiff University School of Medicine, Cardiff CF24 4HQ, UK
*Correspondence: j.mill@exeter.ac.uk
http://dx.doi.org/10.1016/j.ajhg.2017.04.013.
© 2017 The Author(s). This is an open access article under the CC BY license (http://creativecommons.org/licenses/by/4.0/).

the phenotype and DNA methylation, the association statistics will be identical regardless of the selected instrument (Figure S1B), and the HEIDI p value will be non-significant. In contrast, if two separate causal variants are each correlated with the instrument, there will be variation in the results from different instruments (Figure S1C), as indicated by a significant HEIDI p value. It should be noted that this approach is unable to distinguish these two scenarios if the two causal variants are in perfect LD and power is inversely proportional to the strength of the correlation between the two causal variants. Furthermore, the assumptions underlying Mendelian randomization[20] also apply to SMR, and it is possible for variants to act through mechanisms such as horizontal pleiotropy.

By identifying non-significant heterogeneity (HEIDI $p > 0.05$), we identified a refined set of 625 associations between 28 complex traits and 440 DNA methylation sites (Table S2), which can be described as pleiotropic. We were able to test 581 of these associations with mQTLs generated from a second independent whole-blood dataset ($n = 665$; a description of this cohort, referred to as phase 2, can be found here[19]). A highly significant proportion (99.2%; sign test $p = 1.47 \times 10^{-172}$) had the same direction of association across the two datasets (Figure S3), and a large proportion ($n = 337$; 58.0%) satisfied the criteria for a pleiotropic association ($p < 1.04 \times 10^{-6}$ and HEIDI $p > 0.05$) in the replication dataset as well. Out of the GWAS traits tested, height was characterized by the most associations ($n = 193$), an unsurprising observation given that this was the most highly powered GWAS with the largest number of GWAS-significant loci ($n = 423$). Power for SMR analysis is influenced by the power of the GWAS, which differs for each trait considered, making comparisons between traits relatively difficult.

As demonstrated in its original implementation for eQTLs, the SMR approach based on mQTLs has the potential to nominate loci that currently do not have sufficient statistical power to obtain genome-wide significance on the basis of GWAS data alone but that represent candidates for future genetic studies (Table S3). Our SMR analysis of Tanner staging of puberty, for example, identified DNA methylation sites in nine independent loci (annotated to *APEH* [MIM: 102645], *SYNJ2* [MIM: 609410], *IDO2* [MIM: 612129], *PDZRN4* [MIM: 609730], *HTR2A* [MIM: 182135], *CTDP1* [MIM: 604927], *RAE1* [MIM: 603343], and non-genic regions on chromosomes 4 and 16) that do not have a genome-wide-significant ($p < 5 \times 10^{-8}$) variant within 0.5 Mb in the GWAS[21] (Figure S4). In some genomic regions, DNA methylation sites annotated to different genes are associated with the same phenotype; for example, on chromosome 15, sites annotated to *CHRNA5* (MIM: 118505) and *PSMA4* (MIM: 176846) are associated with the number of cigarettes smoked per day (Figure S5), and on chromosome 17, sites annotated to *ERBB2* (MIM: 164870) and *PGAP3* (MIM: 611801) are associated with total cholesterol (Figure S6). Furthermore, 130 DNA methylation sites were found to be associated with multiple complex traits (ranging from two to six traits; Table S4). In many cases, these overlaps are consistent with either reported phenotypic correlations (e.g., cg24631222 and cg04140906 annotated to *CHRNA5* are associated with both schizophrenia and the number of cigarettes smoked per day [Figure S7], two traits that are epidemiologically linked[22,23]) or shared genetic architecture (e.g., cg10583485, annotated to *DOCK7* [MIM: 615730] [Figure S8], is associated with LDL, triglycerides, and total cholesterol, three traits characterized by a strong genetic correlation[24]). Because genetic correlations could account for some of the overlap between traits, we factored in genetic correlations derived from LD score regression,[24] which showed that 30 of the 70 pairs of traits with at least one associated DNA methylation site in common are actually characterized by a genetic correlation < 0.2 (Figure S9).

Multiple DNA methylation sites can be annotated to a single gene, and we identified a total of 337 gene-trait pleiotropic associations with a mean of 1.46 sites associated per gene (range = 1–11). These overlapping associations between a particular complex trait and a gene would not necessarily be expected to be associated in the same direction given that correlation of DNA methylation across a gene is not always positive, and they were not for 20 of the 31 gene-trait associations involving genes with multiple annotated DNA methylation sites. To add further support to the genes prioritized at GWAS loci with the use of blood mQTL data, we aligned these results with SMR analyses performed on publically available whole-blood eQTL data ($n = 5{,}311$; $p < 5 \times 10^{-8}$) described in detail in a recent paper by Westra et al.[15] We identified an overlapping set of 2,724 genes that were (1) annotated to DNA methylation sites influenced by significant mQTLs (involving 7,722 distinct DNA methylation sites) and (2) also transcriptionally influenced by variation at significant eQTLs (involving 2,770 gene expression microarray probes), making them suitable for testing in the SMR framework. It should be noted that one limitation to assessing the relationship between mQTLs and eQTLs is that DNA methylation sites, like SNPs, are annotated to genes according to their location; therefore, a lack of overlap in the associations with a particular gene from the SMR analyses between DNA methylation and gene expression should not necessarily be interpreted as inconsistent evidence. Furthermore, the differences in the sample sizes used for generating the mQTL and eQTL datasets could result in different levels of statistical power to detect QTLs. Of the 337 pleiotropic gene-trait associations identified with mQTLs, 86 (25.5%) were also tested with eQTLs in the SMR framework (Figure S10). Of these, 27 (31.4%) involving 17 complex traits associated with expression at 16 genes also met the criteria for representing pleiotropic associations between the trait and gene expression (SMR $p < 8.38 \times 10^{-6}$ corrected for 5,966 gene expression probes and HEIDI $p > 0.05$) (Table S5). An example of overlapping mQTL and eQTL signals for *RNASET2* (MIM: 612944) on chromosome 6 is presented in Figure 1; both *RNASET2* expression

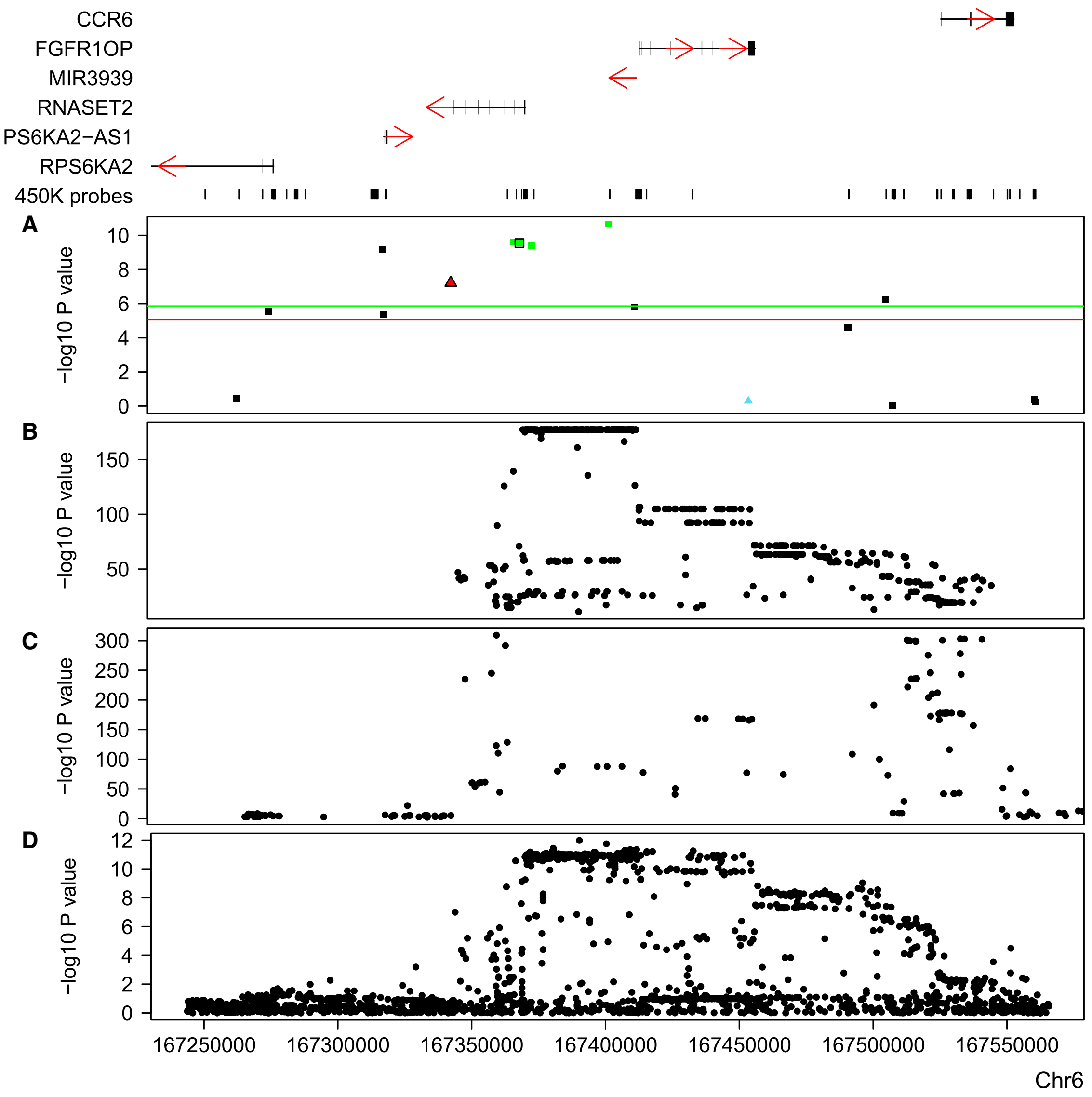

Figure 1. SMR Analysis Using mQTLs and eQTLs Implicates a Role for *RNASET2* in Crohn Disease
Shown is a chromosome 6 genomic region (UCSC Genome Browser hg19: 167,243,095–167,565,882) identified in a recent Crohn disease GWAS performed by Liu et al.[5] Genes located in this region are shown at the top; exons are indicated by thicker bars, and red arrows indicate the direction of transcription. DNA methylation sites interrogated by the Illumina 450K array are indicated by solid vertical lines underneath the genes. The four bottom panels depict the $-\log_{10}$ p value (y axis) against genomic location (x axis) from (A) SMR analysis (black squares represent Illumina 450K array DNA methylation sites, blue triangles represent gene expression probes, and green and red coloring highlight those with a non-significant HEIDI test for DNA methylation and gene expression, respectively), (B) blood mQTL (n = 639) results for the DNA methylation site cg25258033 (outlined in black in A), (C) blood eQTL (n = 5,311) results for ILMN1671565 (outlined in black in A), and (D) the Crohn disease GWAS performed by Liu et al.[5]

(SMR $p = 6.04 \times 10^{-8}$) and DNA methylation at two CpG sites in the first intron of the gene (cg25258033: SMR $p = 2.84 \times 10^{-10}$; cg25258033: SMR $p = 2.50 \times 10^{-10}$) are associated with Crohn disease (MIM: 266600).

Given the tissue-specific and developmentally dynamic nature of gene regulation, we were next interested in examining the consistency of our findings in a different tissue. So, we repeated the SMR analysis on mQTLs identified in our recent analysis of human fetal brain (n = 166; mQTL $p < 1 \times 10^{-8}$; a detailed description of this dataset can be found here[18]). The majority (75.4%) of SNP-DNA methylation relationships identified for SMR analysis in whole blood are characterized by a consistent direction of effect when tested in fetal brain (sign test $p = 4.94 \times 10^{-324}$; Figure S11). Despite the strong concordance of mQTL effects across tissues, the smaller number of samples used for generating the fetal brain dataset (n = 166) means that only a subset (4,691 [13.3%]) of these mQTL associations passed our mQTL significance threshold ($p < 1 \times 10^{-8}$) and were included in the subsequent SMR analyses; almost all of these (96.0%; sign test $p < 2.2 \times 10^{-308}$) were characterized by the same direction of effect in both tissues (Figure S12). Of the 625 pleiotropic associations identified with whole-blood mQTLs, 84 (13.5%) involved

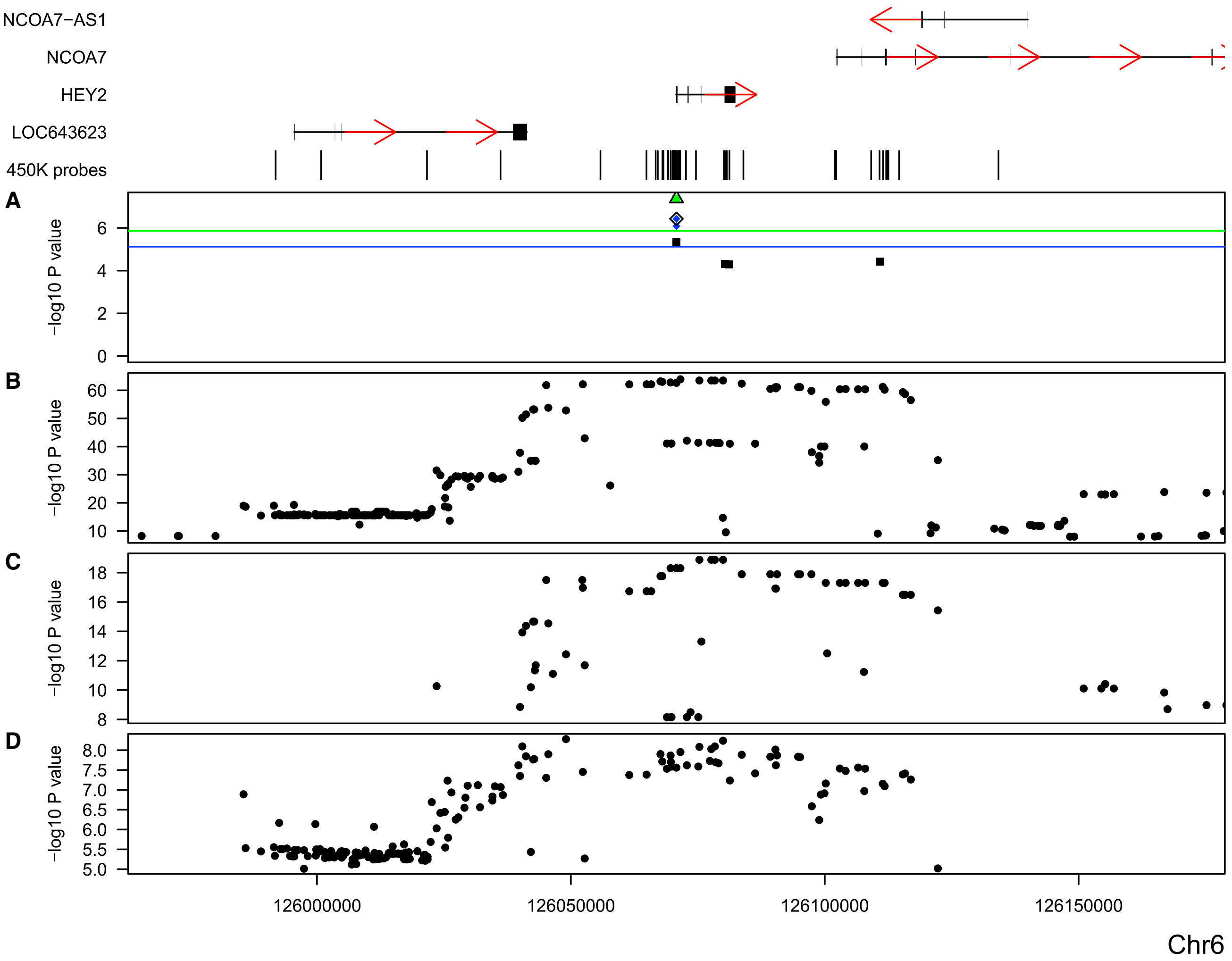

Figure 2. SMR Analysis Using Whole-Blood and Fetal Brain mQTL Data Implicates a Role for *HEY2* in Migraine
Shown is a chromosome 6 genomic region (UCSC Genome Browser hg19: 125,970,800–126,170,800) identified in a recent migraine GWAS performed by Gormley et al.[25] Genes located in this region are shown at the top; exons are indicated by thicker bars, and red arrows indicate the direction of transcription. The four bottom panels depict the $-\log_{10}$ p value (y axis) against genomic location (x axis) from (A) SMR analysis (points represent DNA methylation sites interrogated by the Illumina 450K array, squares and diamonds indicate SMR tests from blood and fetal brain mQTLs, respectively, and green squares and blue diamonds highlight those with a non-significant HEIDI test for blood and fetal brain, respectively), mQTL results for the DNA methylation site cg05901451 (outlined in black in A) in (B) blood (n = 639) and (C) fetal brain (n = 166), and (D) the migraine GWAS performed by Gormley et al. (n = 59,674 case and 316,078 control samples).[25]

a DNA methylation site that also had a significant fetal brain mQTL ($p < 1 \times 10^{-8}$), meaning it could be tested with the SMR framework (Figure S13). Of these 84 pleiotropic associations, 35 (41.7%) met the criteria (i.e., SMR $p < 5.40 \times 10^{-6}$ corrected for 9,265 DNA methylation sites tested and HEIDI $p > 0.05$) for also having a pleiotropic association involving nine complex traits in fetal brain (Table S6). Whereas six (17.1%) of the site-trait associations involved brain-related phenotypes (five for schizophrenia and one for migraine [MIM: 157300]), the majority (82.9%) involved traits that are presumed to affect other tissues (e.g., total cholesterol and Crohn disease), suggesting that effects are common across tissues. Figure 2 summarizes SMR analysis across the *HEY2-NOCA7* region on chromosome 6, which was implicated in a recent GWAS of migraine.[25] Manhattan plots for the genetic analysis of cg05901451, located in the 5′ UTR of *HEY2* (MIM: 604674), in whole blood and fetal brain show a profile highly comparable to that of the migraine GWAS, consistent with overlapping genetic signals influencing DNA methylation in both tissues and migraine.[25]

Finally, comparing the SMR results across multiple complex traits gives a potential insight into shared pleiotropic associations between pairs of traits. We performed hierarchical clustering of SMR results for 38 complex traits, selected because they were tested against a minimum of 20,000 DNA methylation sites, to identify consistent signatures (Figure S14). Figure 3, for example, depicts the association statistics for 43 DNA methylation sites associated with Crohn disease (SMR $p < 1.42 \times 10^{-6}$) across all 38 phenotypes; interestingly, we observed a highly concordant profile between Crohn disease and ulcerative colitis

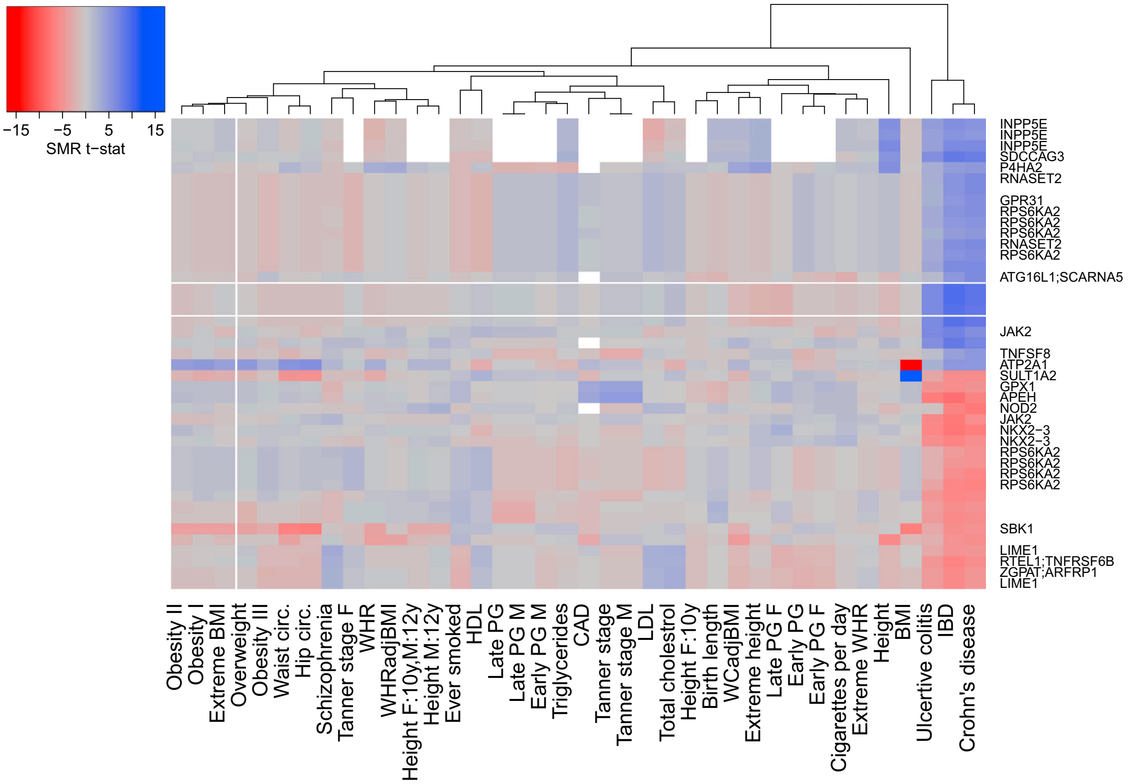

Figure 3. Heatmap of the SMR Results for 32 DNA Methylation Sites Associated with Crohn Disease across 38 GWAS Datasets
Each square in the heatmap represents the t-statistic (b_SMR/se_SMR) of the GWAS trait (columns) for a DNA methylation site (row; n = 32) associated with Crohn disease. Only phenotypes (n = 38) tested against at least 20,000 DNA methylation sites were included in this comparison. SMR $p < 1.38 \times 10^{-6}$ and HEIDI $p > 0.05$.

across all associated sites, consistent with the strong genetic correlation between these traits (Figure S9). The SMR results might highlight which genes are characterized by shared effects between traits. There is also a notable overlap with BMI, waist, and hip circumference at specific loci (i.e., *ATP2A1* [MIM: 108730], *SULT1A2* [MIM: 601292], and *SBK1* [MIM: 300374]), an interesting observation given the negligible genetic correlations between these traits and Crohn disease.

Together, these analyses demonstrate the utility of the SMR approach for identifying instances where complex traits and variable DNA methylation are pleiotropically associated with genetic variation. This approach could facilitate our understanding of the functional consequences of genetic risk variants for a range of complex traits and facilitate the localization and prioritization of specific genes within genomic regions identified by GWASs.

Supplemental Data

Supplemental Data include 12 figures and 6 tables and can be found with this article online at http://dx.doi.org/10.1016/j.ajhg.2017.04.013.

Acknowledgments

This work was funded by a grant from the UK Medical Research Council (MR/K013807/1) to J.M.

Received: October 25, 2016
Accepted: April 19, 2017
Published: May 18, 2017

Web Resources

ChunkChromosome, http://genome.sph.umich.edu/wiki/ChunkChromosome
Fetal brain mQTL data, http://epigenetics.essex.ac.uk/mQTL/All_Imputed_BonfSignificant_mQTLs.csv.gz
OMIM, http://www.omim.org
SMR, http://cnsgenomics.com/software/smr/download.html
UCSC Genome Browser, https://genome.ucsc.edu/
Whole-blood eQTL data, http://cnsgenomics.com/software/smr/westra_eqtl_hg19.zip
Whole-blood mQTL data, http://epigenetics.essex.ac.uk/schizophrenia/BloodmQTL.csv.tgz

References

1. Locke, A.E., Kahali, B., Berndt, S.I., Justice, A.E., Pers, T.H., Day, F.R., Powell, C., Vedantam, S., Buchkovich, M.L., Yang, J., et al.; LifeLines Cohort Study; ADIPOGen Consortium; AGEN-BMI Working Group; CARDIOGRAMplusC4D Consortium; CKDGen Consortium; GLGC; ICBP; MAGIC Investigators; MuTHER Consortium; MIGen Consortium; PAGE Consortium; ReproGen Consortium; GENIE Consortium; and International Endogene Consortium (2015). Genetic

studies of body mass index yield new insights for obesity biology. Nature *518*, 197–206.
2. Wood, A.R., Esko, T., Yang, J., Vedantam, S., Pers, T.H., Gustafsson, S., Chu, A.Y., Estrada, K., Luan, J., Kutalik, Z., et al.; Electronic Medical Records and Genomics (eMEMERGEGE) Consortium; MIGen Consortium; PAGEGE Consortium; and LifeLines Cohort Study (2014). Defining the role of common variation in the genomic and biological architecture of adult human height. Nat. Genet. *46*, 1173–1186.
3. Nikpay, M., Goel, A., Won, H.H., Hall, L.M., Willenborg, C., Kanoni, S., Saleheen, D., Kyriakou, T., Nelson, C.P., Hopewell, J.C., et al.; CARDIoGRAMplusC4D Consortium (2015). A comprehensive 1,000 Genomes-based genome-wide association meta-analysis of coronary artery disease. Nat. Genet. *47*, 1121–1130.
4. Schunkert, H., König, I.R., Kathiresan, S., Reilly, M.P., Assimes, T.L., Holm, H., Preuss, M., Stewart, A.F., Barbalic, M., Gieger, C., et al.; Cardiogenics; and CARDIoGRAM Consortium (2011). Large-scale association analysis identifies 13 new susceptibility loci for coronary artery disease. Nat. Genet. *43*, 333–338.
5. Liu, J.Z., van Sommeren, S., Huang, H., Ng, S.C., Alberts, R., Takahashi, A., Ripke, S., Lee, J.C., Jostins, L., Shah, T., et al.; International Multiple Sclerosis Genetics Consortium; and International IBD Genetics Consortium (2015). Association analyses identify 38 susceptibility loci for inflammatory bowel disease and highlight shared genetic risk across populations. Nat. Genet. *47*, 979–986.
6. Lambert, J.C., Ibrahim-Verbaas, C.A., Harold, D., Naj, A.C., Sims, R., Bellenguez, C., DeStafano, A.L., Bis, J.C., Beecham, G.W., Grenier-Boley, B., et al.; European Alzheimer's Disease Initiative (EADI); Genetic and Environmental Risk in Alzheimer's Disease; Alzheimer's Disease Genetic Consortium; and Cohorts for Heart and Aging Research in Genomic Epidemiology (2013). Meta-analysis of 74,046 individuals identifies 11 new susceptibility loci for Alzheimer's disease. Nat. Genet. *45*, 1452–1458.
7. van Rheenen, W., Shatunov, A., Dekker, A.M., McLaughlin, R.L., Diekstra, F.P., Pulit, S.L., van der Spek, R.A., Võsa, U., de Jong, S., Robinson, M.R., et al.; PARALS Registry; SLALOM Group; SLAP Registry; FALS Sequencing Consortium; SLAGEN Consortium; and NNIPPS Study Group (2016). Genome-wide association analyses identify new risk variants and the genetic architecture of amyotrophic lateral sclerosis. Nat. Genet. *48*, 1043–1048.
8. Ripke, S., Wray, N.R., Lewis, C.M., Hamilton, S.P., Weissman, M.M., Breen, G., Byrne, E.M., Blackwood, D.H., Boomsma, D.I., Cichon, S., et al.; Major Depressive Disorder Working Group of the Psychiatric GWAS Consortium (2013). A mega-analysis of genome-wide association studies for major depressive disorder. Mol. Psychiatry *18*, 497–511.
9. Schizophrenia Working Group of the Psychiatric Genomics Consortium (2014). Biological insights from 108 schizophrenia-associated genetic loci. Nature *511*, 421–427.
10. Psychiatric GWAS Consortium Bipolar Disorder Working Group (2011). Large-scale genome-wide association analysis of bipolar disorder identifies a new susceptibility locus near ODZ4. Nat. Genet. *43*, 977–983.
11. Schaub, M.A., Boyle, A.P., Kundaje, A., Batzoglou, S., and Snyder, M. (2012). Linking disease associations with regulatory information in the human genome. Genome Res. *22*, 1748–1759.
12. Ernst, J., Kheradpour, P., Mikkelsen, T.S., Shoresh, N., Ward, L.D., Epstein, C.B., Zhang, X., Wang, L., Issner, R., Coyne, M., et al. (2011). Mapping and analysis of chromatin state dynamics in nine human cell types. Nature *473*, 43–49.
13. Maurano, M.T., Humbert, R., Rynes, E., Thurman, R.E., Haugen, E., Wang, H., Reynolds, A.P., Sandstrom, R., Qu, H., Brody, J., et al. (2012). Systematic localization of common disease-associated variation in regulatory DNA. Science *337*, 1190–1195.
14. Li, M., Jaffe, A.E., Straub, R.E., Tao, R., Shin, J.H., Wang, Y., Chen, Q., Li, C., Jia, Y., Ohi, K., et al. (2016). A human-specific AS3MT isoform and BORCS7 are molecular risk factors in the 10q24.32 schizophrenia-associated locus. Nat. Med. *22*, 649–656.
15. Westra, H.J., Peters, M.J., Esko, T., Yaghootkar, H., Schurmann, C., Kettunen, J., Christiansen, M.W., Fairfax, B.P., Schramm, K., Powell, J.E., et al. (2013). Systematic identification of trans eQTLs as putative drivers of known disease associations. Nat. Genet. *45*, 1238–1243.
16. Zhu, Z., Zhang, F., Hu, H., Bakshi, A., Robinson, M.R., Powell, J.E., Montgomery, G.W., Goddard, M.E., Wray, N.R., Visscher, P.M., and Yang, J. (2016). Integration of summary data from GWAS and eQTL studies predicts complex trait gene targets. Nat. Genet. *48*, 481–487.
17. Bird, A. (2002). DNA methylation patterns and epigenetic memory. Genes Dev. *16*, 6–21.
18. Hannon, E., Spiers, H., Viana, J., Pidsley, R., Burrage, J., Murphy, T.M., Troakes, C., Turecki, G., O'Donovan, M.C., Schalkwyk, L.C., et al. (2016). Methylation QTLs in the developing brain and their enrichment in schizophrenia risk loci. Nat. Neurosci. *19*, 48–54.
19. Hannon, E., Dempster, E., Viana, J., Burrage, J., Smith, A.R., Macdonald, R., St Clair, D., Mustard, C., Breen, G., Therman, S., et al. (2016). An integrated genetic-epigenetic analysis of schizophrenia: evidence for co-localization of genetic associations and differential DNA methylation. Genome Biol. *17*, 176.
20. Smith, G.D., and Ebrahim, S. (2003). 'Mendelian randomization': can genetic epidemiology contribute to understanding environmental determinants of disease? Int. J. Epidemiol. *32*, 1–22.
21. Cousminer, D.L., Stergiakouli, E., Berry, D.J., Ang, W., Groen-Blokhuis, M.M., Körner, A., Siitonen, N., Ntalla, I., Marinelli, M., Perry, J.R., et al.; ReproGen Consortium; and Early Growth Genetics Consortium (2014). Genome-wide association study of sexual maturation in males and females highlights a role for body mass and menarche loci in male puberty. Hum. Mol. Genet. *23*, 4452–4464.
22. de Leon, J., and Diaz, F.J. (2005). A meta-analysis of worldwide studies demonstrates an association between schizophrenia and tobacco smoking behaviors. Schizophr. Res. *76*, 135–157.
23. McClave, A.K., McKnight-Eily, L.R., Davis, S.P., and Dube, S.R. (2010). Smoking characteristics of adults with selected lifetime mental illnesses: results from the 2007 National Health Interview Survey. Am. J. Public Health *100*, 2464–2472.
24. Bulik-Sullivan, B., Finucane, H.K., Anttila, V., Gusev, A., Day, F.R., Loh, P.R., Duncan, L., Perry, J.R., Patterson, N., Robinson, E.B., et al.; ReproGen Consortium; Psychiatric Genomics Consortium; and Genetic Consortium for Anorexia Nervosa of the Wellcome Trust Case Control Consortium 3 (2015). An atlas of genetic correlations across human diseases and traits. Nat. Genet. *47*, 1236–1241.
25. Gormley, P., Anttila, V., Winsvold, B.S., Palta, P., Esko, T., Pers, T.H., Farh, K.H., Cuenca-Leon, E., Muona, M., Furlotte, N.A., et al.; International Headache Genetics Consortium (2016). Meta-analysis of 375,000 individuals identifies 38 susceptibility loci for migraine. Nat. Genet. *48*, 856–866.

The American Journal of Human Genetics, Volume *100*

Supplemental Data

Pleiotropic Effects of Trait-Associated Genetic Variation on DNA Methylation: Utility for Refining GWAS Loci

Eilis Hannon, Mike Weedon, Nicholas Bray, Michael O'Donovan, and Jonathan Mill

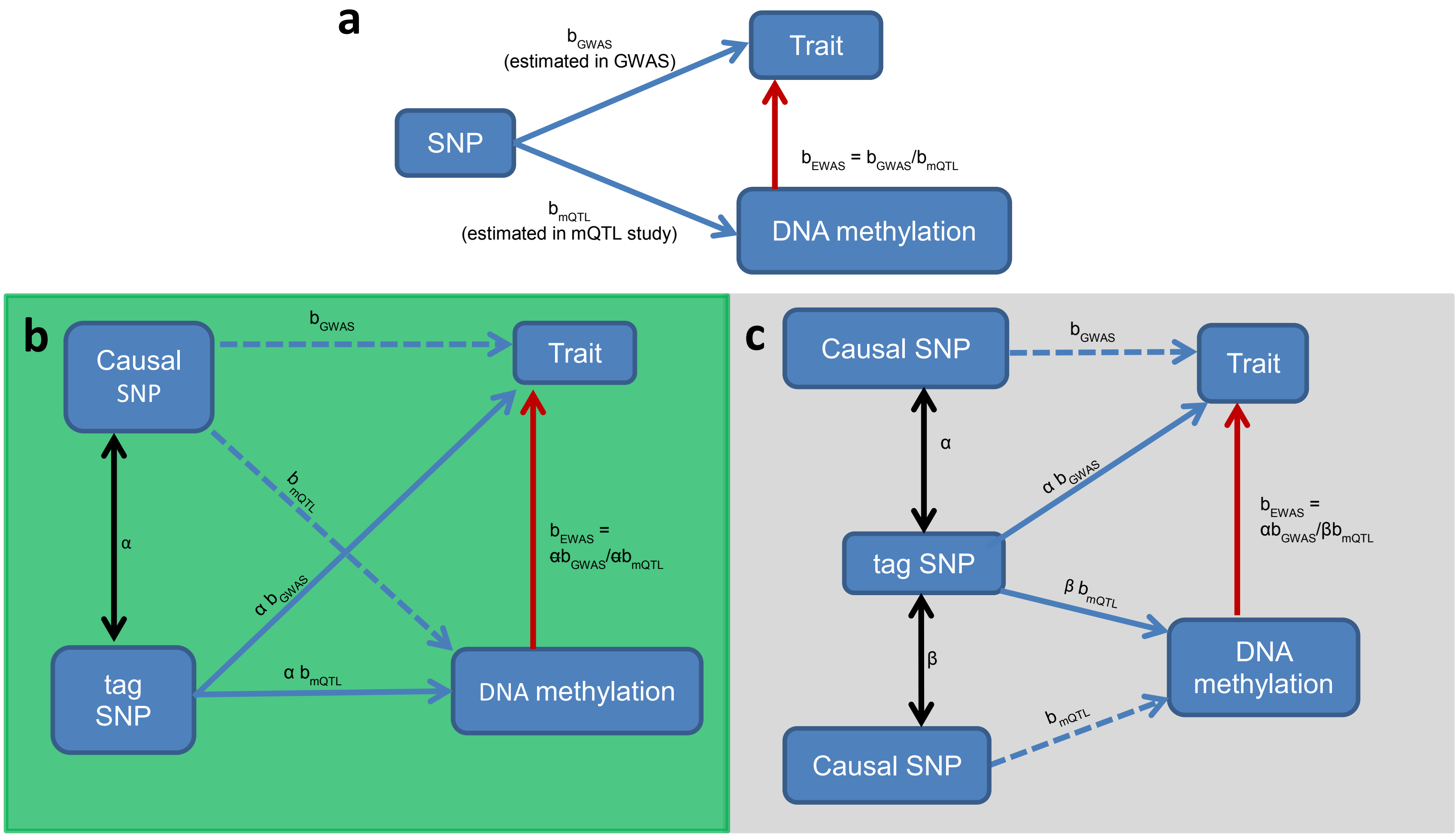

Figure S1: Schematic of the SMR analysis approach used in this study. Panel **a**) demonstrates the first stage of the SMR analysis, which tests for an association between DNA methylation and a trait of interest. Blue solid arrows represent known information taken from GWAS or mQTL results, while red arrows indicate the relationship being derived. The second stage of the SMR analysis aims to distinguish between two scenarios, pleiotropy and linkage, depicted in panels **b**) and **c**) respectively. In these figures dashed blue arrows indicate the true causal associations estimated via "tag SNPs". "tag SNPs" are highly correlated (represented by solid black arrows) with the "causal SNP" quantified as α or β. In panel **b**) as the same causal SNP is associated with both the trait of interest and DNA methylation at a specific site; there is only one correlation statistic (α), which is cancelled out when estimating the effect b_{EWAS}. In contrast, in panel **c**) there are distinct causal SNPs for DNA methylation and the trait, and therefore two correlations with the "tag SNP" (α and β), which do not cancel each other out. Therefore, the estimate of b_{EWAS} will exhibit heterogeneity when different "tag SNPs" are tested, whereas in the scenario depicted in panel **b**) the estimate of b_{EWAS} will be consistent regardless of the choice of "tag SNP". GWAS- genome-wide association analysis study; EWAS- epigenome wide association analysis study.

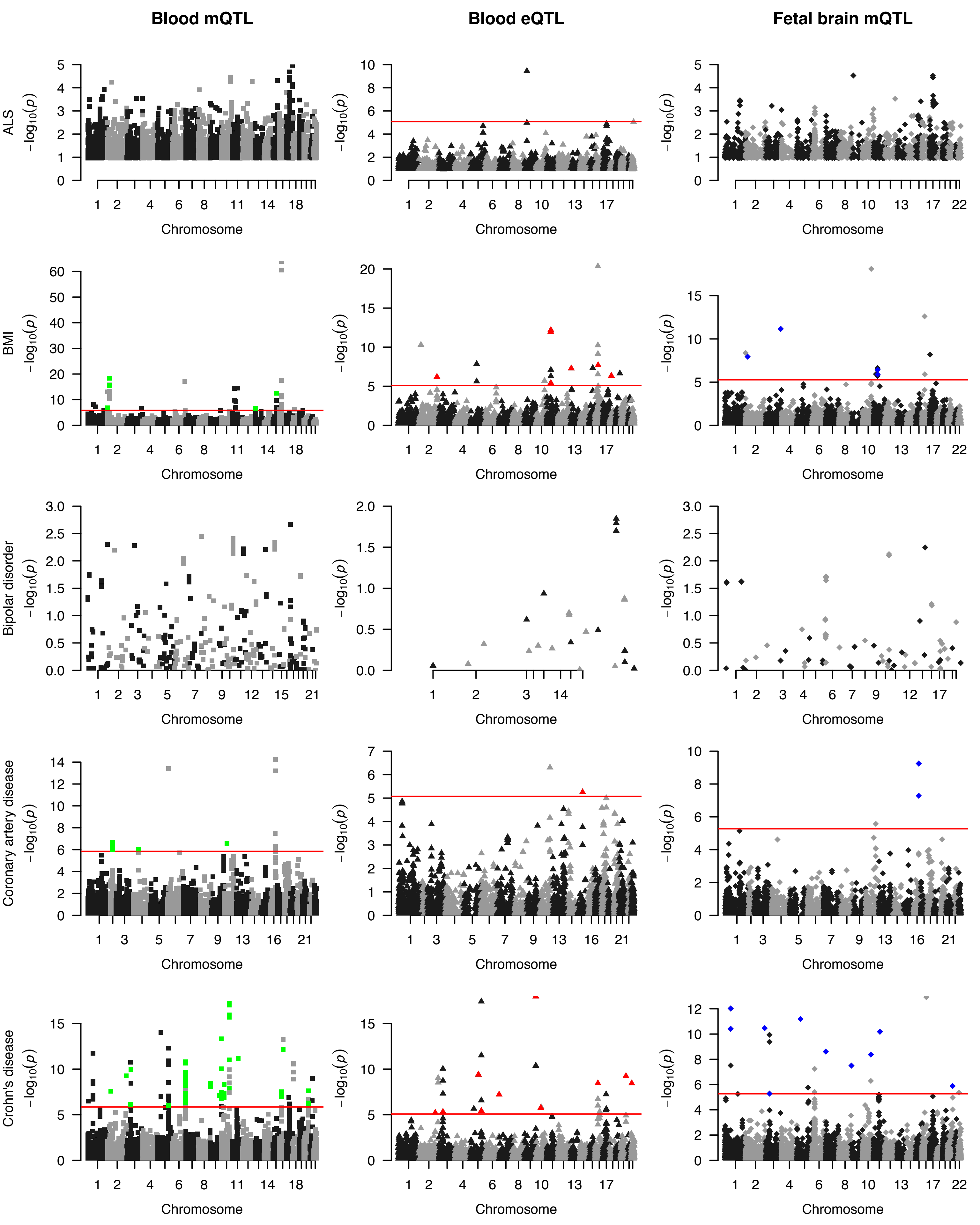

Blood mQTL
Blood eQTL
Fetal brain mQTL
ALS
BMI
Bipolar disorder
Coronary artery disease
Crohn's disease
$-\log_{10}(p)$
Chromosome

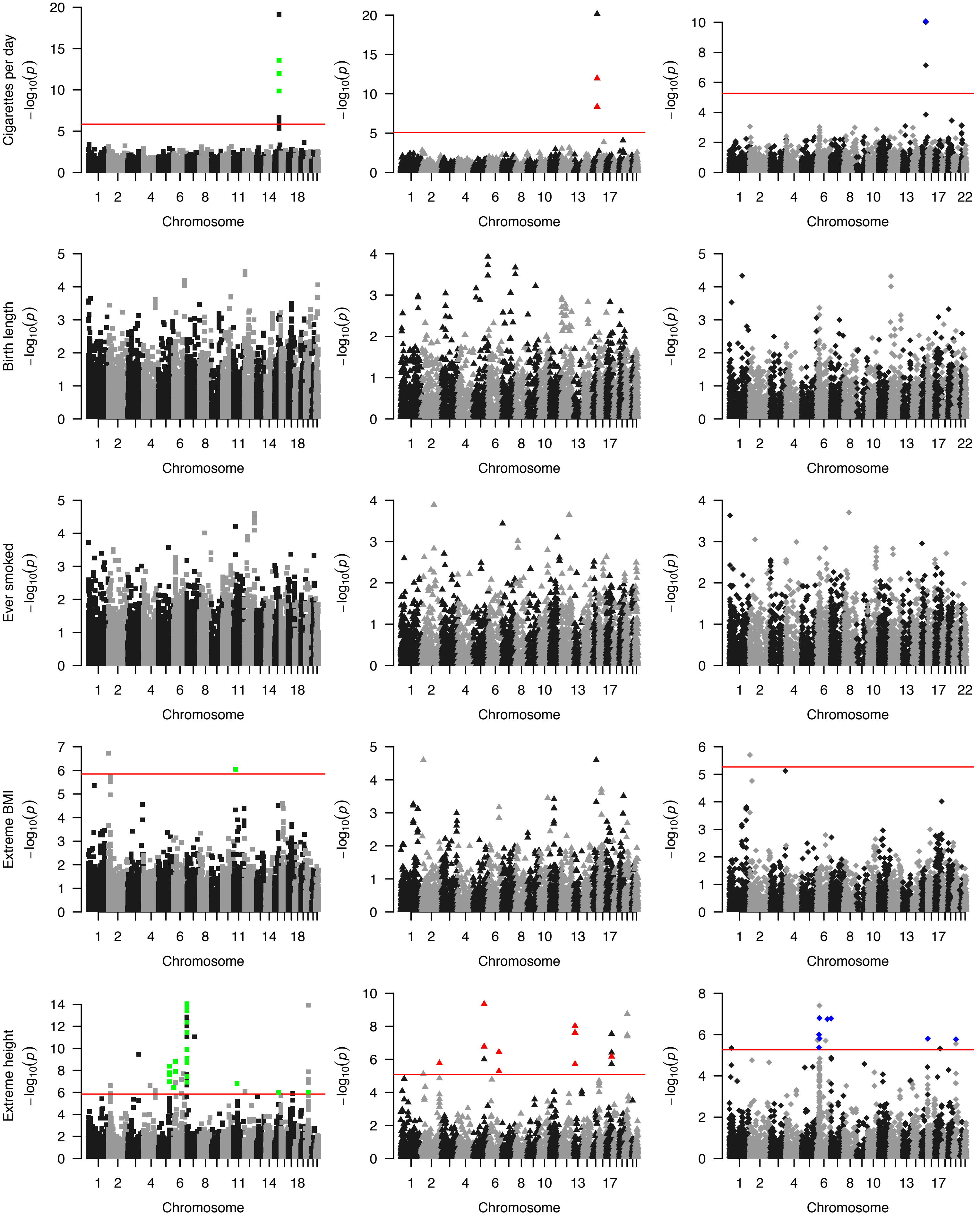

Cigarettes per day
Birth length
Ever smoked
Extreme BMI
Extreme height
$-\log_{10}(p)$
Chromosome
1 2 4 6 8 11 14 18
1 2 4 6 8 10 13 17
1 2 4 6 8 10 13 17 22

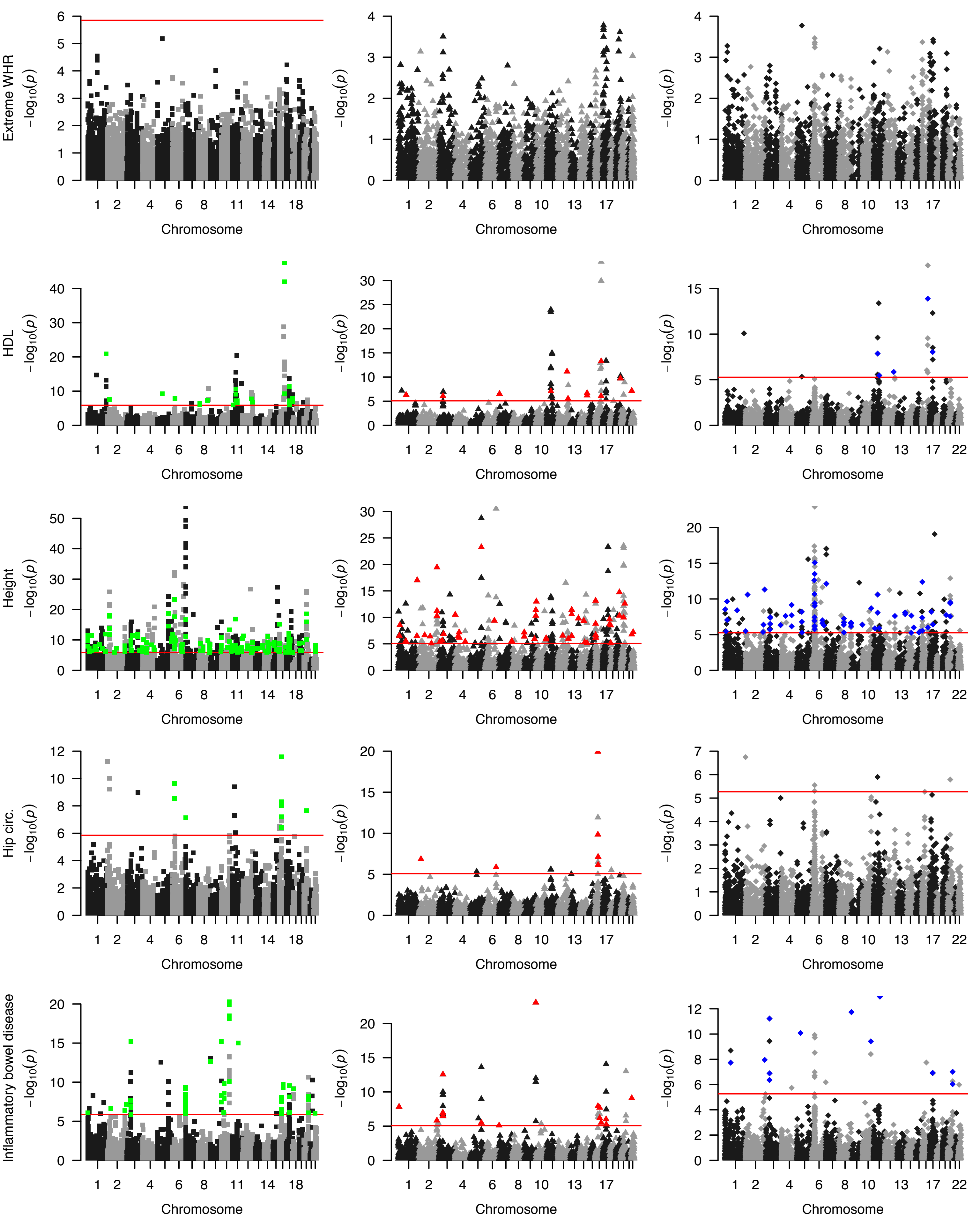
Extreme WHR
HDL
Height
Hip circ.
Inflammatory bowel disease
$-\log_{10}(p)$
Chromosome

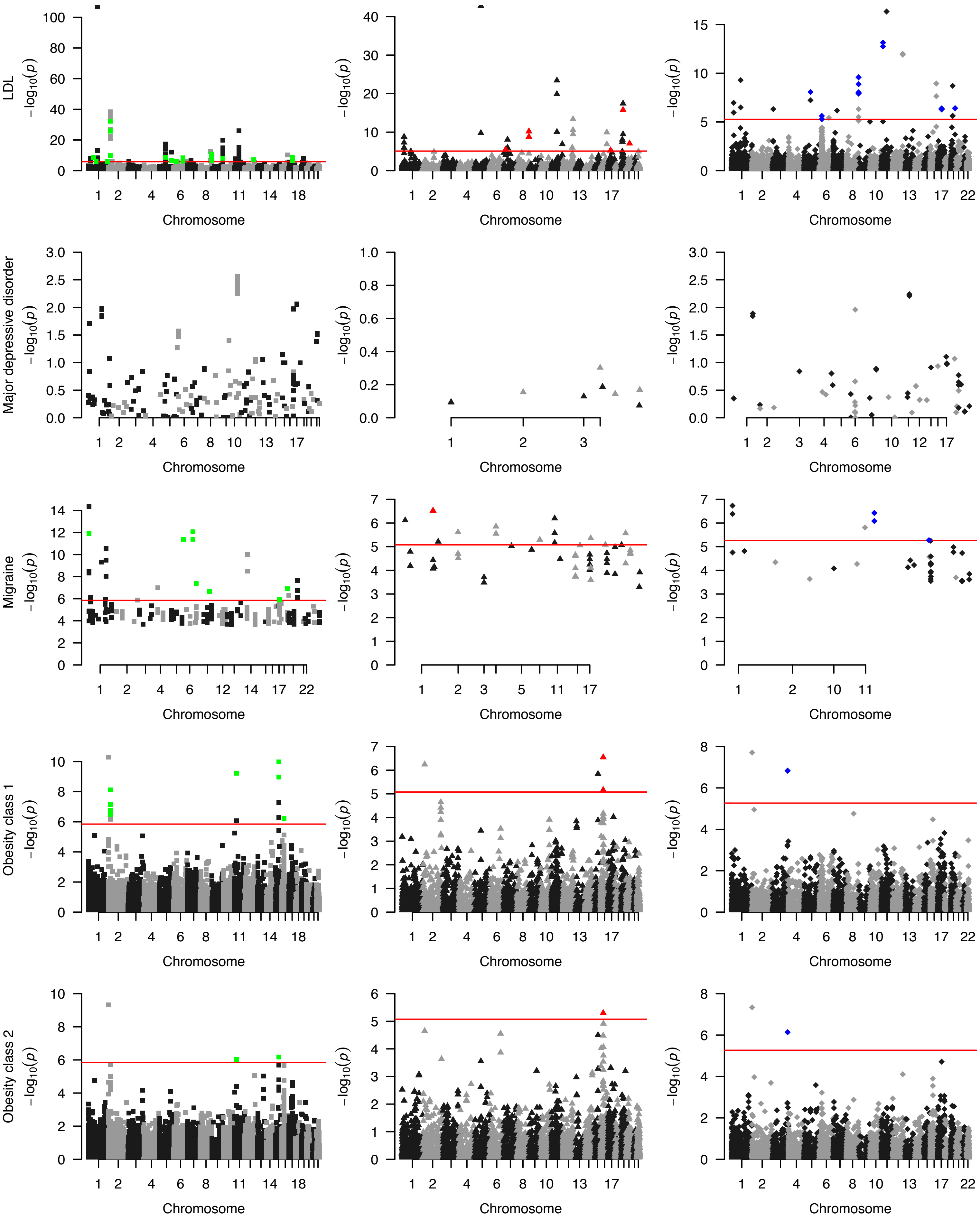
LDL
Major depressive disorder
Migraine
Obesity class 1
Obesity class 2
−log10(p)
Chromosome

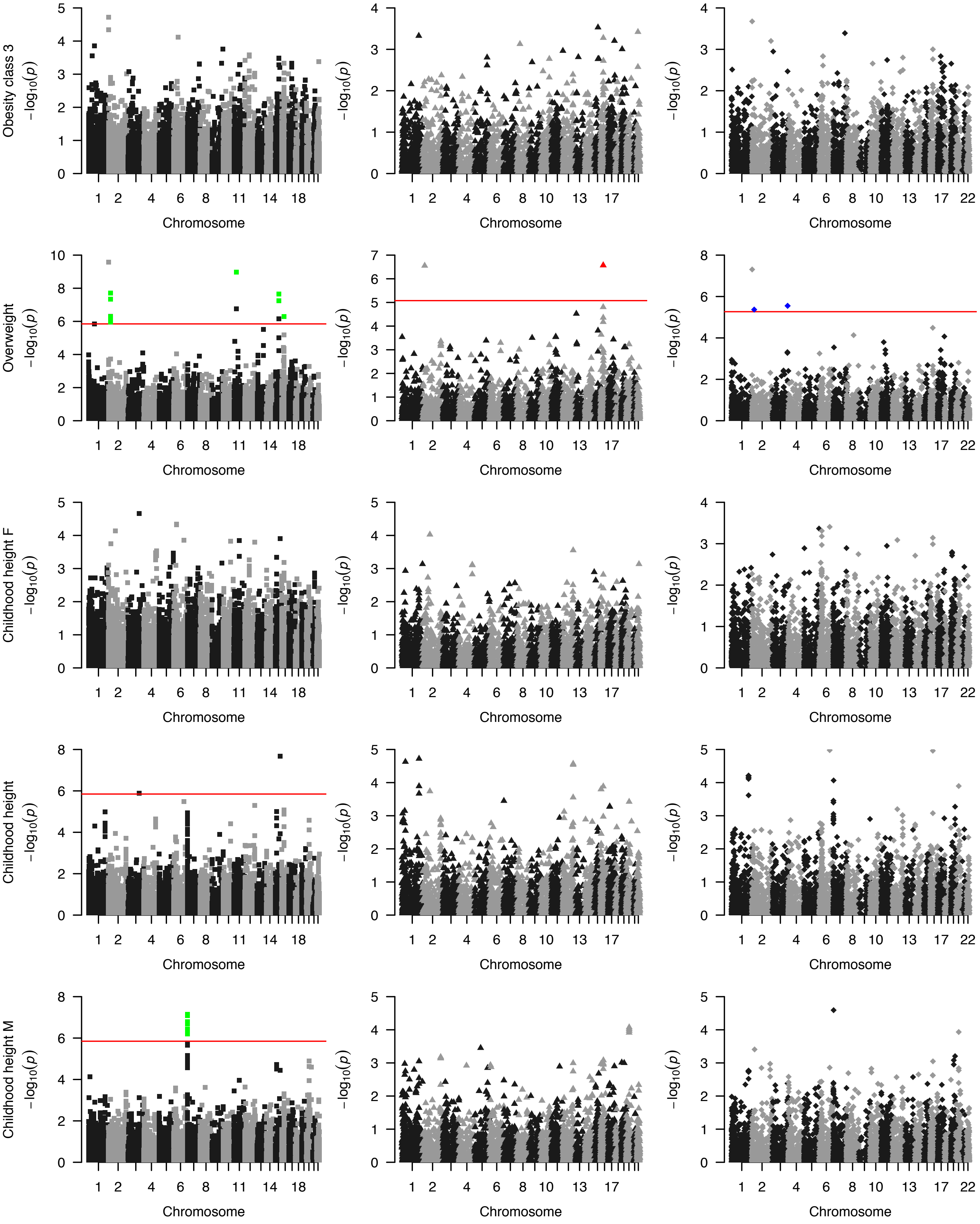
Obesity class 3
Overweight
Childhood height F
Childhood height
Childhood height M
−log10(p)
Chromosome
1 2 4 6 8 11 14 18
1 2 4 6 8 10 13 17
1 2 4 6 8 10 13 17 22

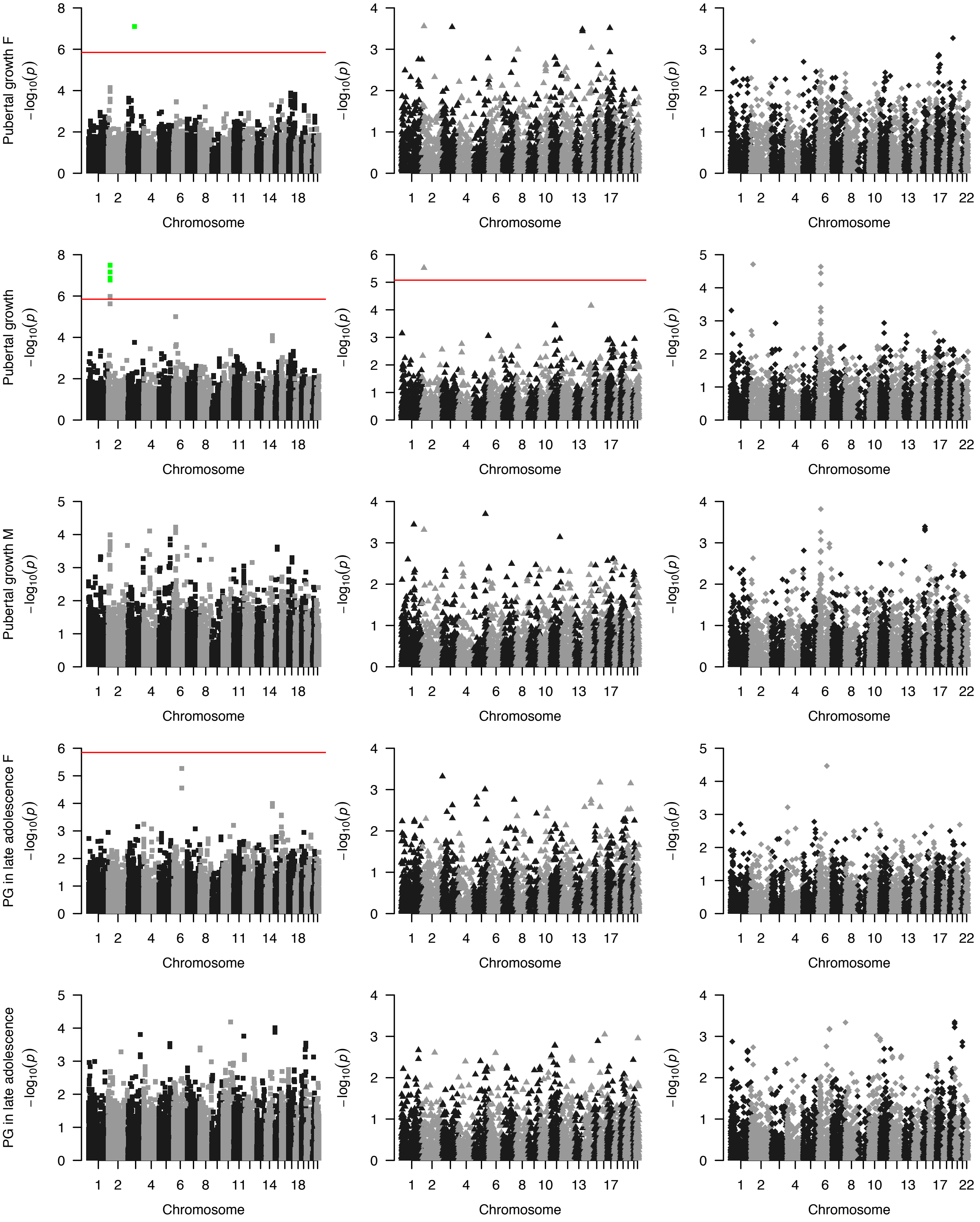

Pubertal growth F
Pubertal growth
Pubertal growth M
PG in late adolescence F
PG in late adolescence
$-\log_{10}(p)$
Chromosome

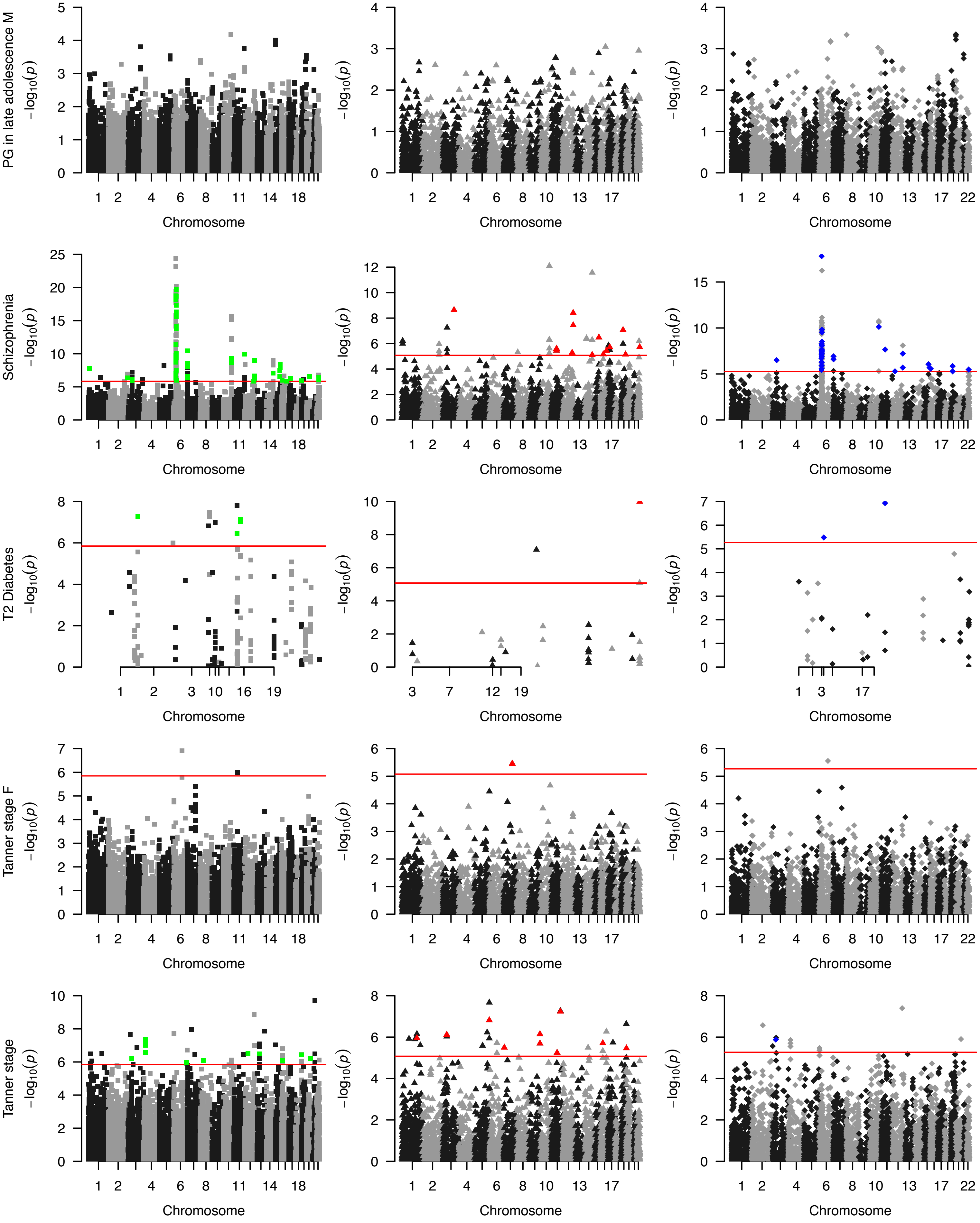

PG in late adolescence M
Schizophrenia
T2 Diabetes
Tanner stage F
Tanner stage
−log10(p)
Chromosome

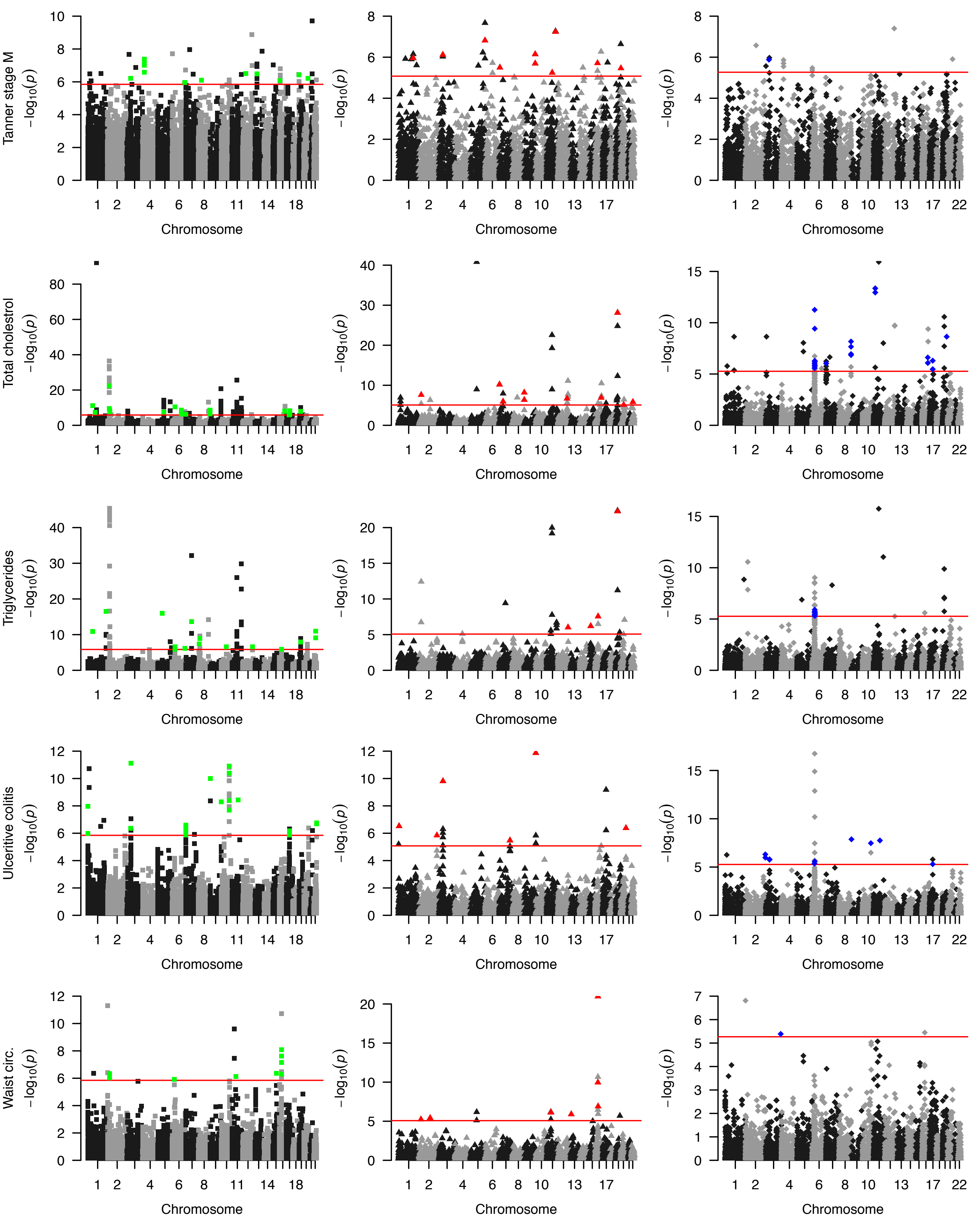

Tanner stage M
Total cholestrol
Triglycerides
Ulceritive colitis
Waist circ.
$-\log_{10}(p)$
Chromosome

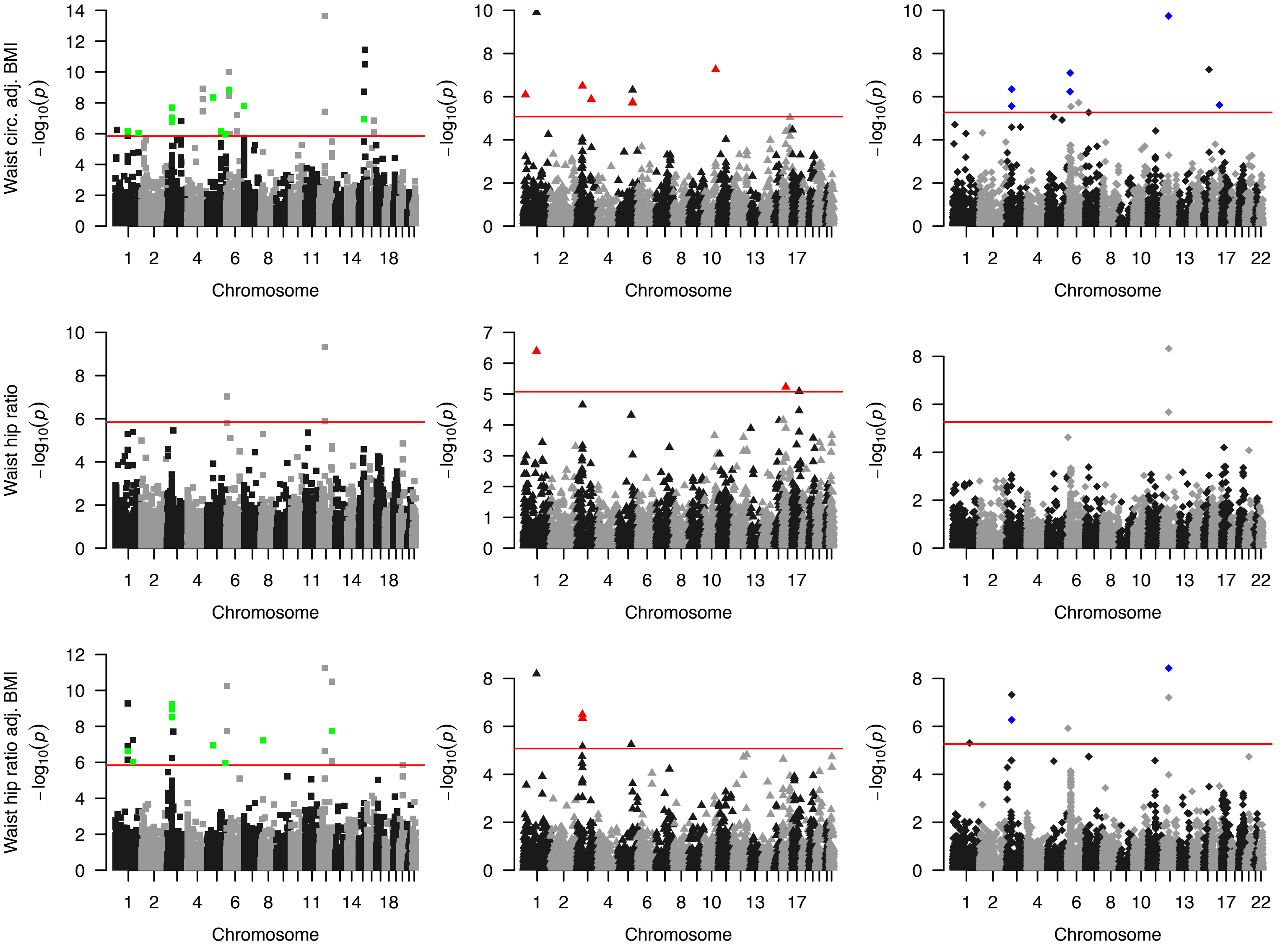

Waist circ. adj. BMI
Waist hip ratio
Waist hip ratio adj. BMI
$-\log_{10}(p)$
Chromosome

Figure S2: Manhattan plots of Summarised Mendelian Randomisation (SMR) tests for pleiotropic effects between complex traits and DNA methylation. Shown on the y-axis of each plot is the –log10 *P*-value from the SMR analysis. Panels on the left display results using DNA methylation quantitative trait loci (mQTL) generated from blood, panels in the middle display results using gene expression quantitative trait loci (eQTL) generated from blood and panels on the right display results using mQTL in fetal brain tissue. Each point represents an SMR test for a particular DNA methylation site or gene expression probe. The red horizontal line represents the genome-wide multiple testing significance threshold (blood mQTL: $P < 1.42 \times 10^{-6}$; blood eQTL: $P < 8.38 \times 10^{-6}$; fetal brain mQTL: $P < 2.14 \times 10^{-6}$); green, red and blue points highlight the significant SMR tests from blood mQTL, blood eQTL and fetal brain mQTL, respectively, which are not characterized by significant heterogeneity (i.e. $P > 0.05$), indicating pleiotropic relationships between that trait and either DNA methylation or gene expression.

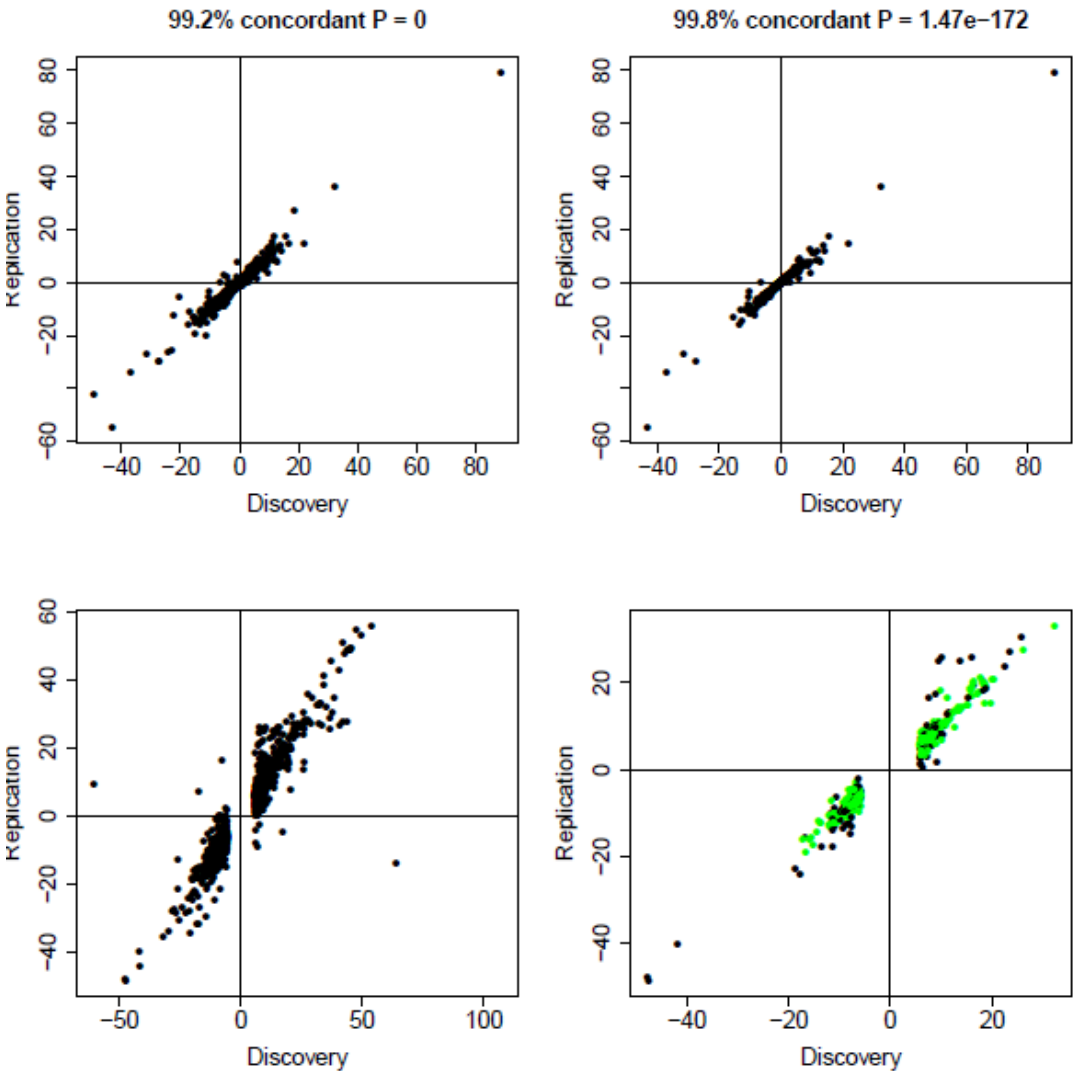

Figure S3: SMR results replicate in a second mQTL dataset. Shown are scatterplots of the association between DNA methylation sites and complex traits comparing results from our original 'discovery' cohort (X-axis) and our 'replication' cohort (Y-axis). The top row plots the regression coefficient (b_SMR) and the bottom row plots the signed log SMR P value for the discovery cohort and replication cohorts. Panels on the left contains all 1,723 associations identified in the discovery cohort (SMR P < 1.42 x 10-6) and tested in the replication cohort; panels on the right contains all 581 pleiotropic associations identified in the discovery cohort (SMR P < 1.42 x 10-6 and HEIDI P > 0.05) and tested in replication cohort. In the bottom right-hand panel, the green points indicate the sites with HEIDI P > 0.05 in the replication dataset. Details of the discovery and replication cohorts can be found in this manuscript[1] refered to as Phase 1 and Phase 2 respectively.

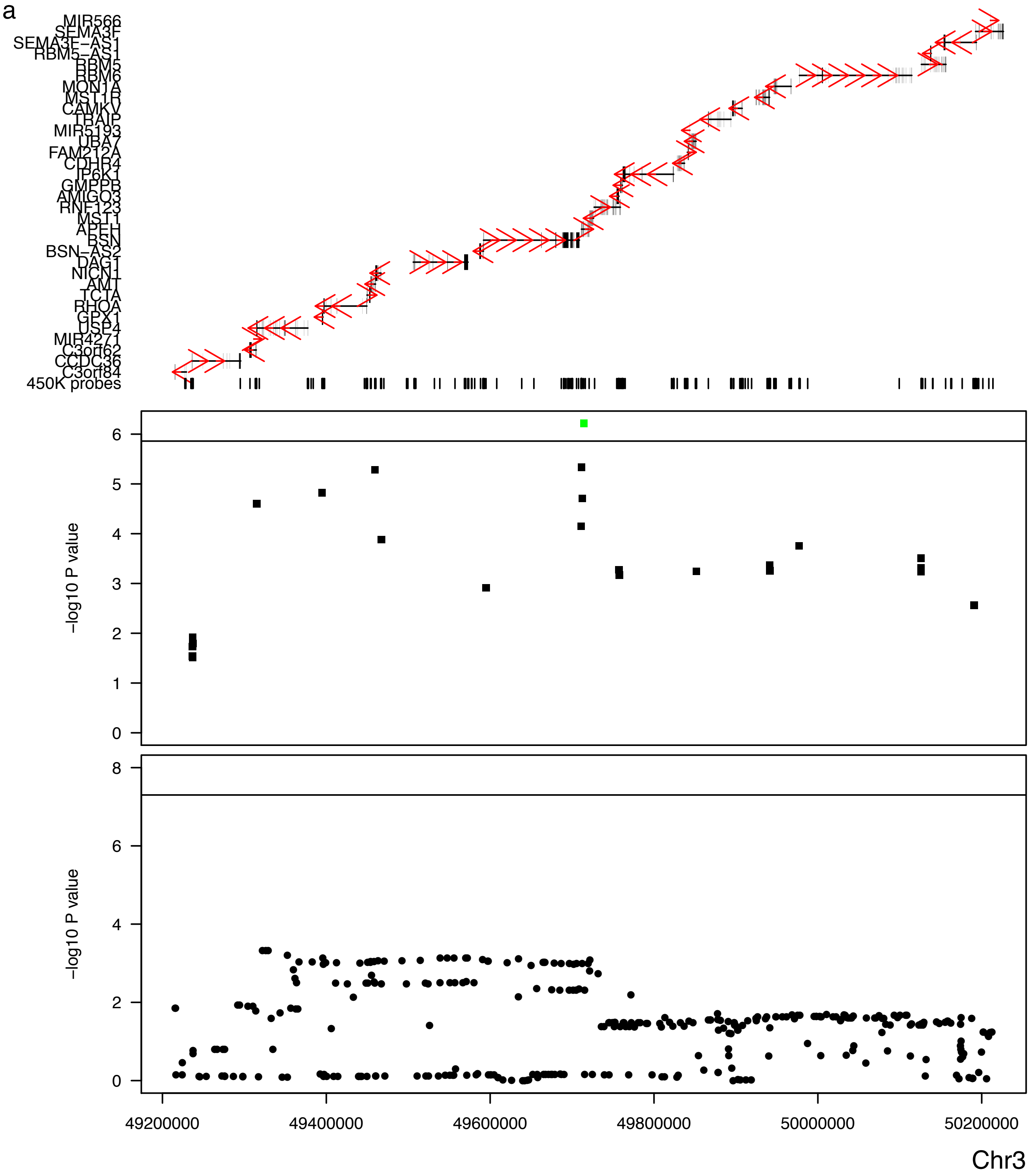
a
MIR566
SEMA3F
SEMA3F−AS1
RBM5−AS1
RBM5
RBM6
MON1A
MST1R
CAMKV
TRAIP
MIR5193
UBA7
FAM212A
CDHR4
IP6K1
GMPPB
AMIGO3
RNF123
MST1
APEH
BSN
BSN−AS2
DAG1
NICN1
AMT
TCTA
RHOA
GPX1
USP4
MIR4271
C3orf62
CCDC36
C3orf84
450K probes
−log10 P value
−log10 P value
49200000
49400000
49600000
49800000
50000000
50200000
Chr3

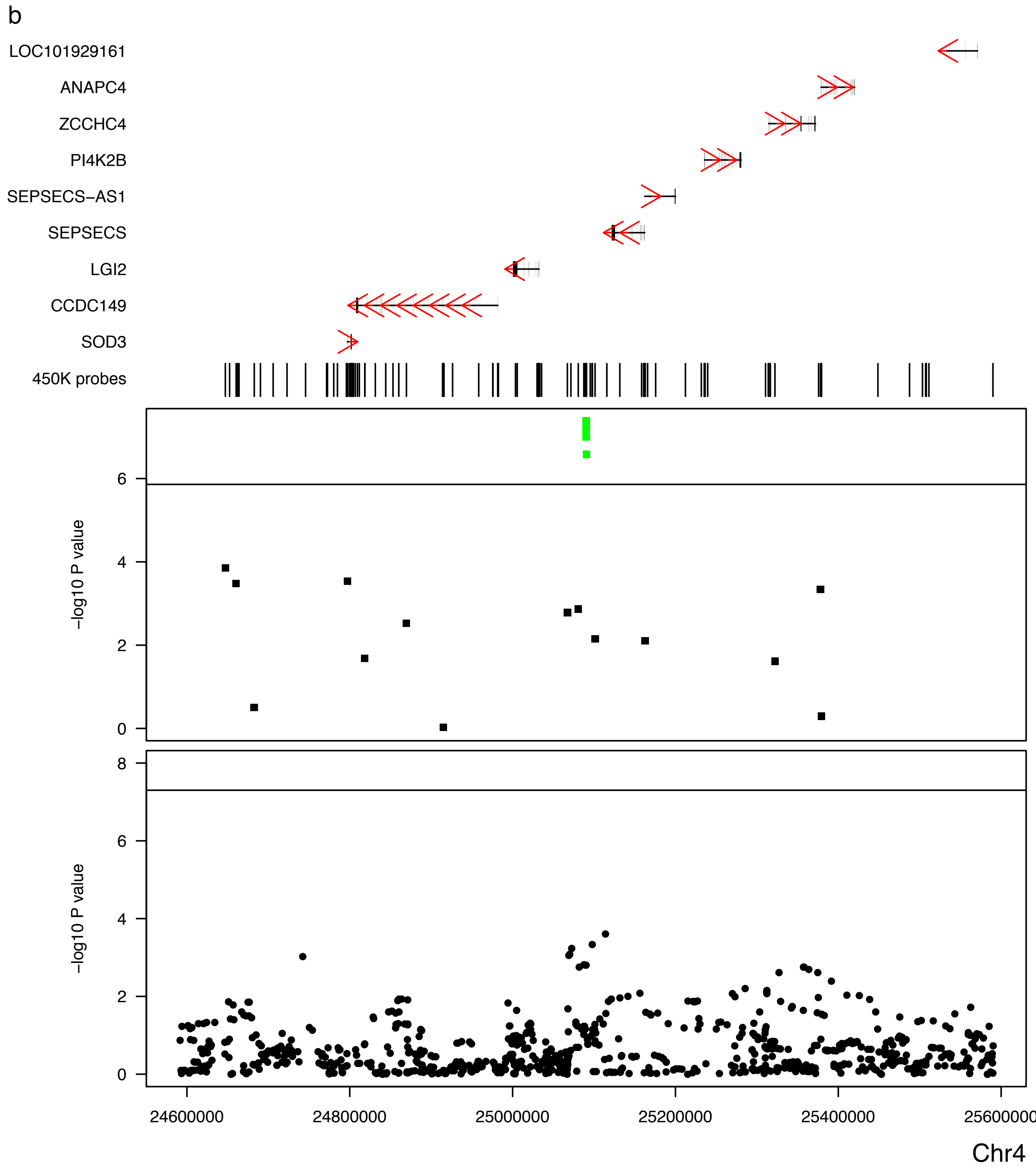

b
LOC101929161
ANAPC4
ZCCHC4
PI4K2B
SEPSECS−AS1
SEPSECS
LGI2
CCDC149
SOD3
450K probes
−log10 P value
0
2
4
6
−log10 P value
0
2
4
6
8
24600000
24800000
25000000
25200000
25400000
25600000
Chr4

c

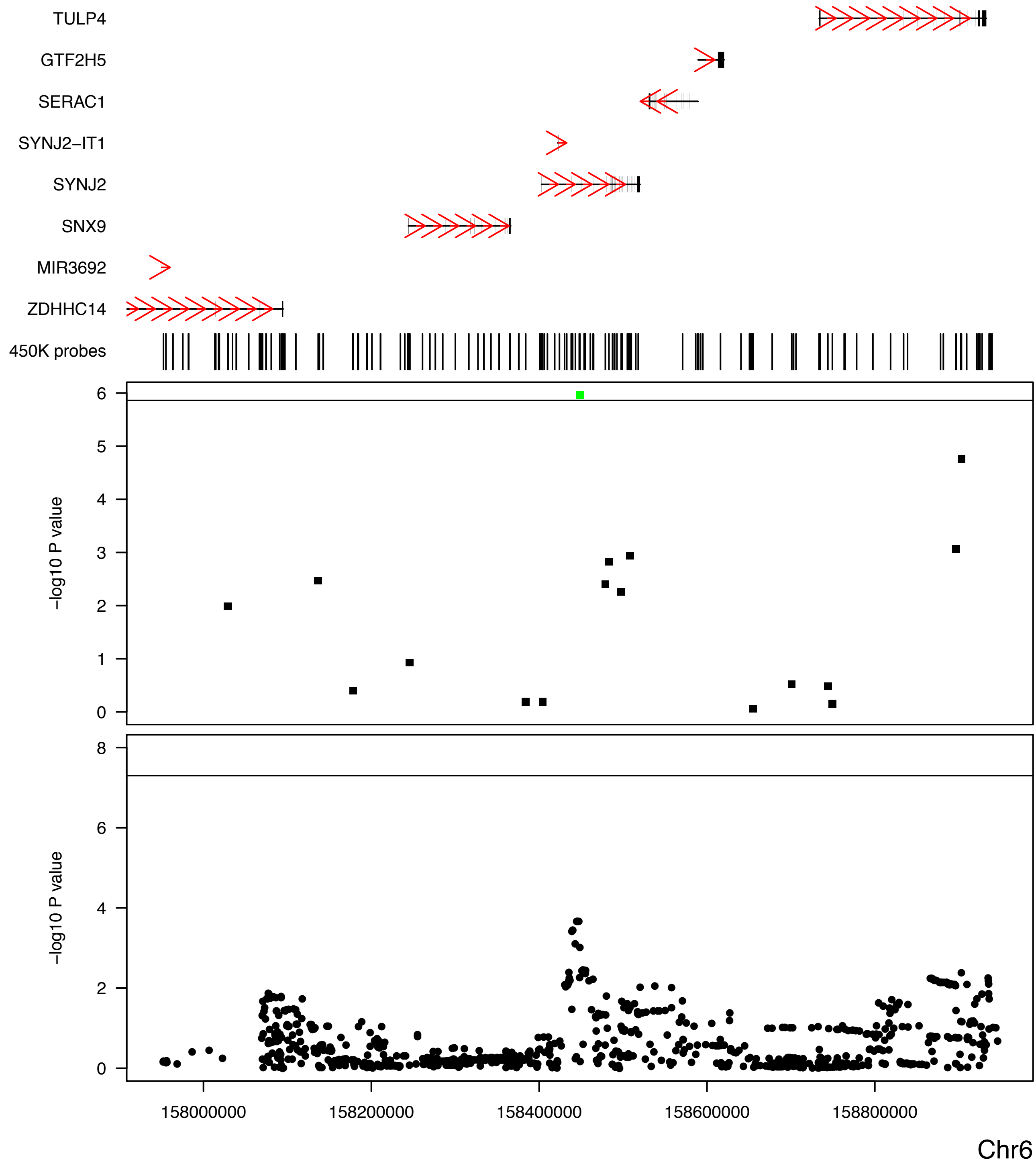

TULP4
GTF2H5
SERAC1
SYNJ2−IT1
SYNJ2
SNX9
MIR3692
ZDHHC14
450K probes
−log10 P value
0
1
2
3
4
5
6
−log10 P value
0
2
4
6
8
158000000
158200000
158400000
158600000
158800000
Chr6

d

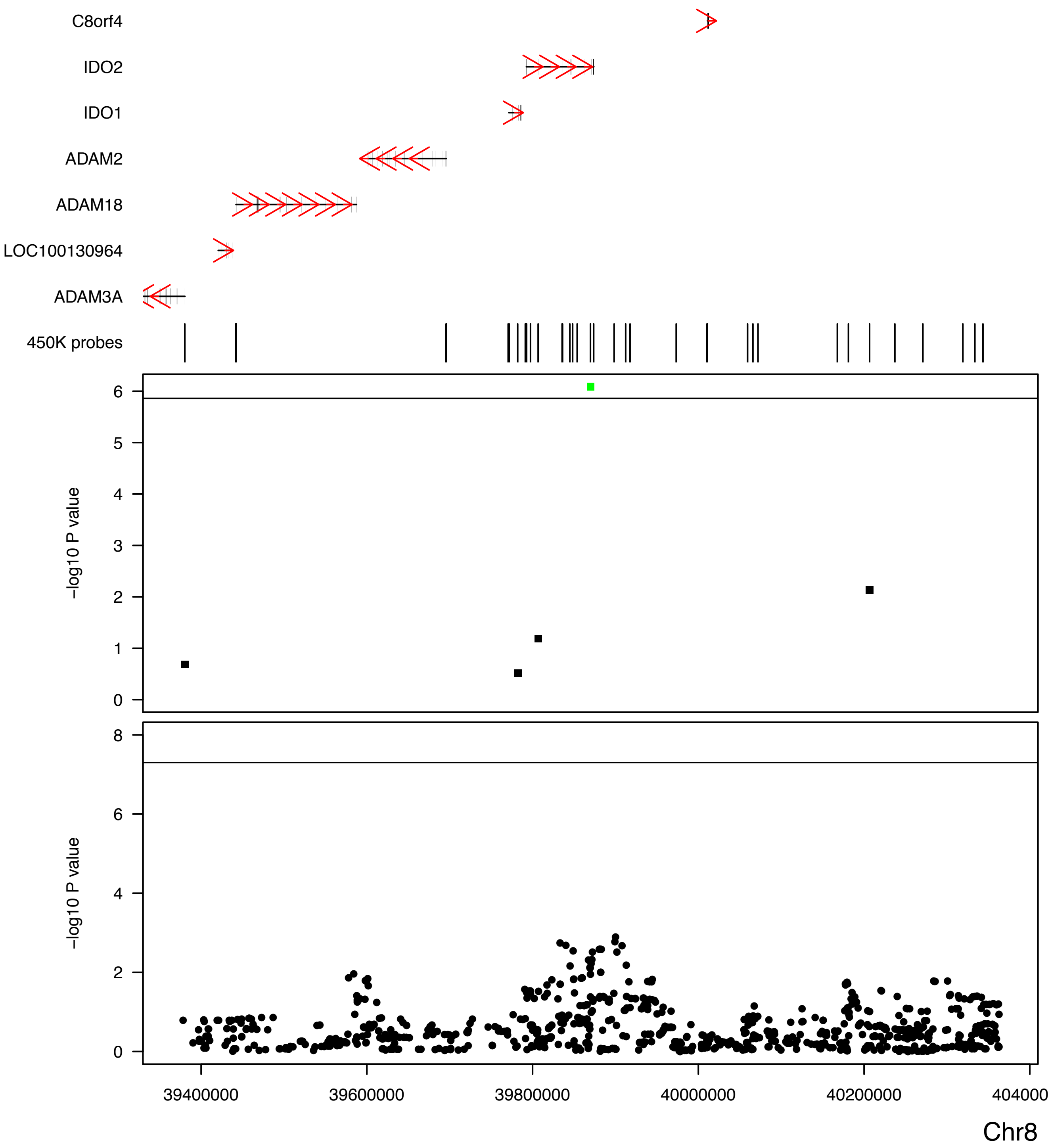
C8orf4
IDO2
IDO1
ADAM2
ADAM18
LOC100130964
ADAM3A
450K probes
−log10 P value
6
5
4
3
2
1
0
−log10 P value
8
6
4
2
0
39400000
39600000
39800000
40000000
40200000
404000
Chr8

e

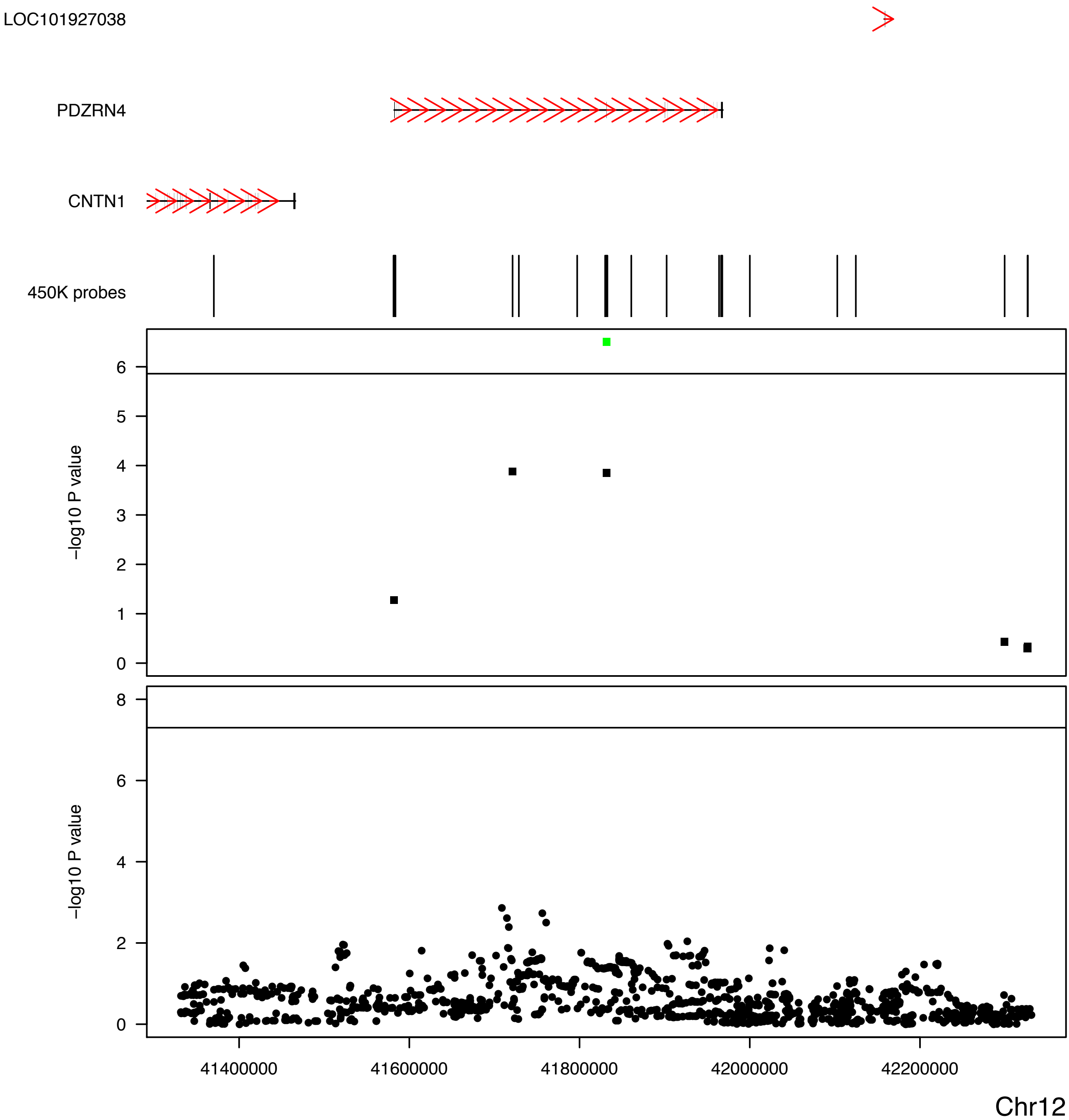
LOC101927038
PDZRN4
CNTN1
450K probes
−log10 P value
6
5
4
3
2
1
0
−log10 P value
8
6
4
2
0
41400000
41600000
41800000
42000000
42200000
Chr12

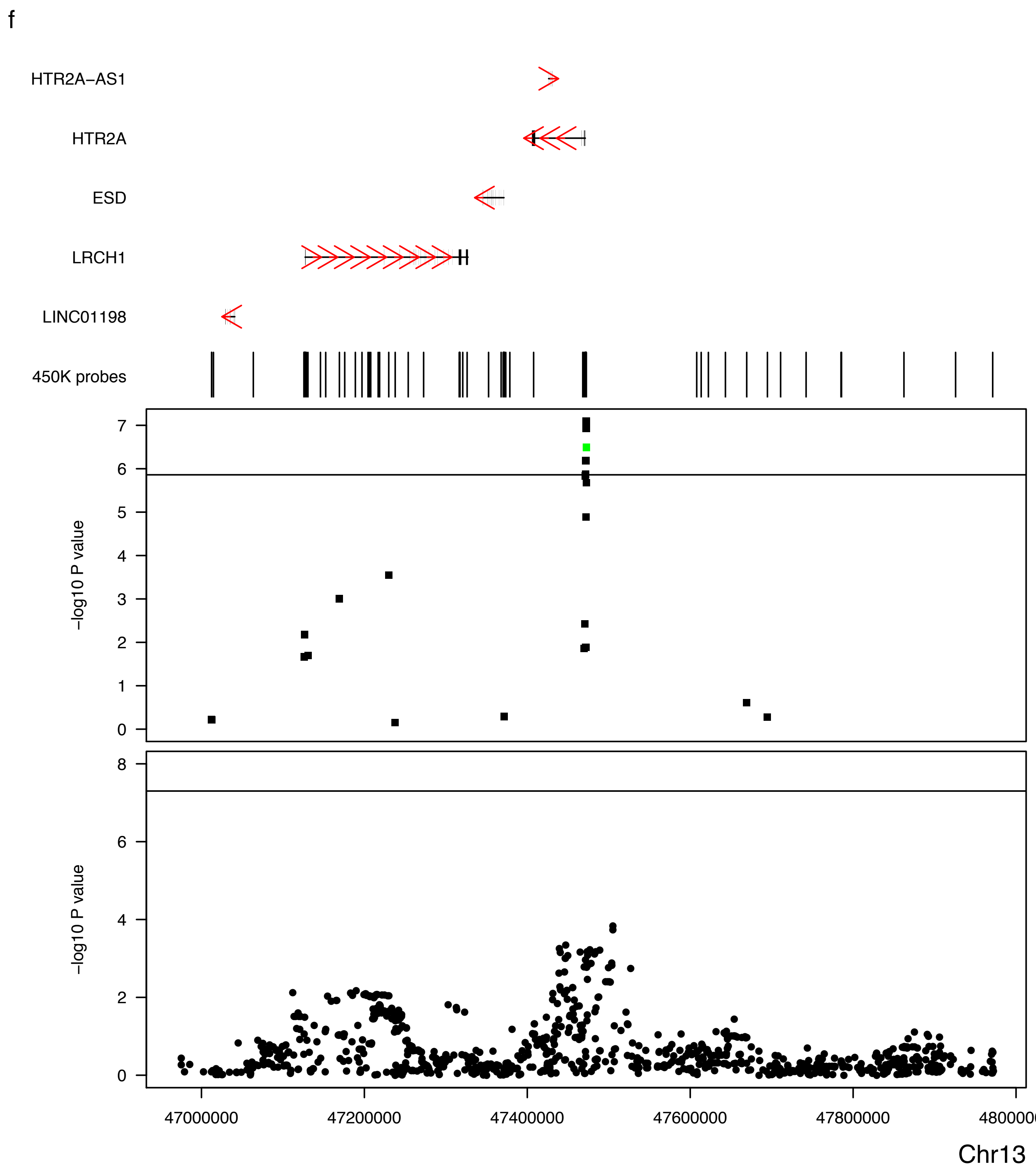
f
HTR2A−AS1
HTR2A
ESD
LRCH1
LINC01198
450K probes
−log10 P value
−log10 P value
47000000
47200000
47400000
47600000
47800000
Chr13

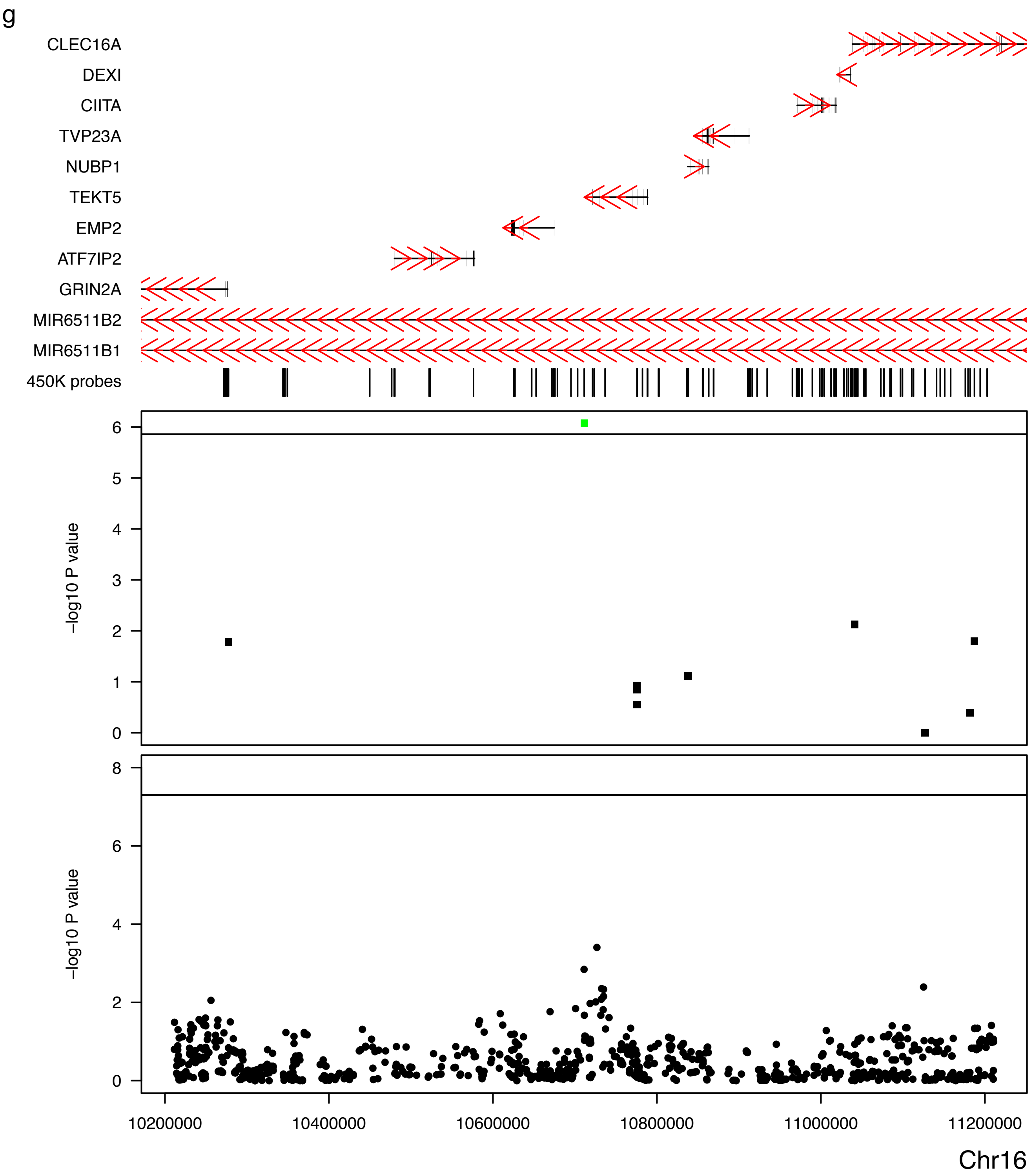

g
CLEC16A
DEXI
CIITA
TVP23A
NUBP1
TEKT5
EMP2
ATF7IP2
GRIN2A
MIR6511B2
MIR6511B1
450K probes
−log10 P value
−log10 P value
10200000
10400000
10600000
10800000
11000000
11200000
Chr16

h

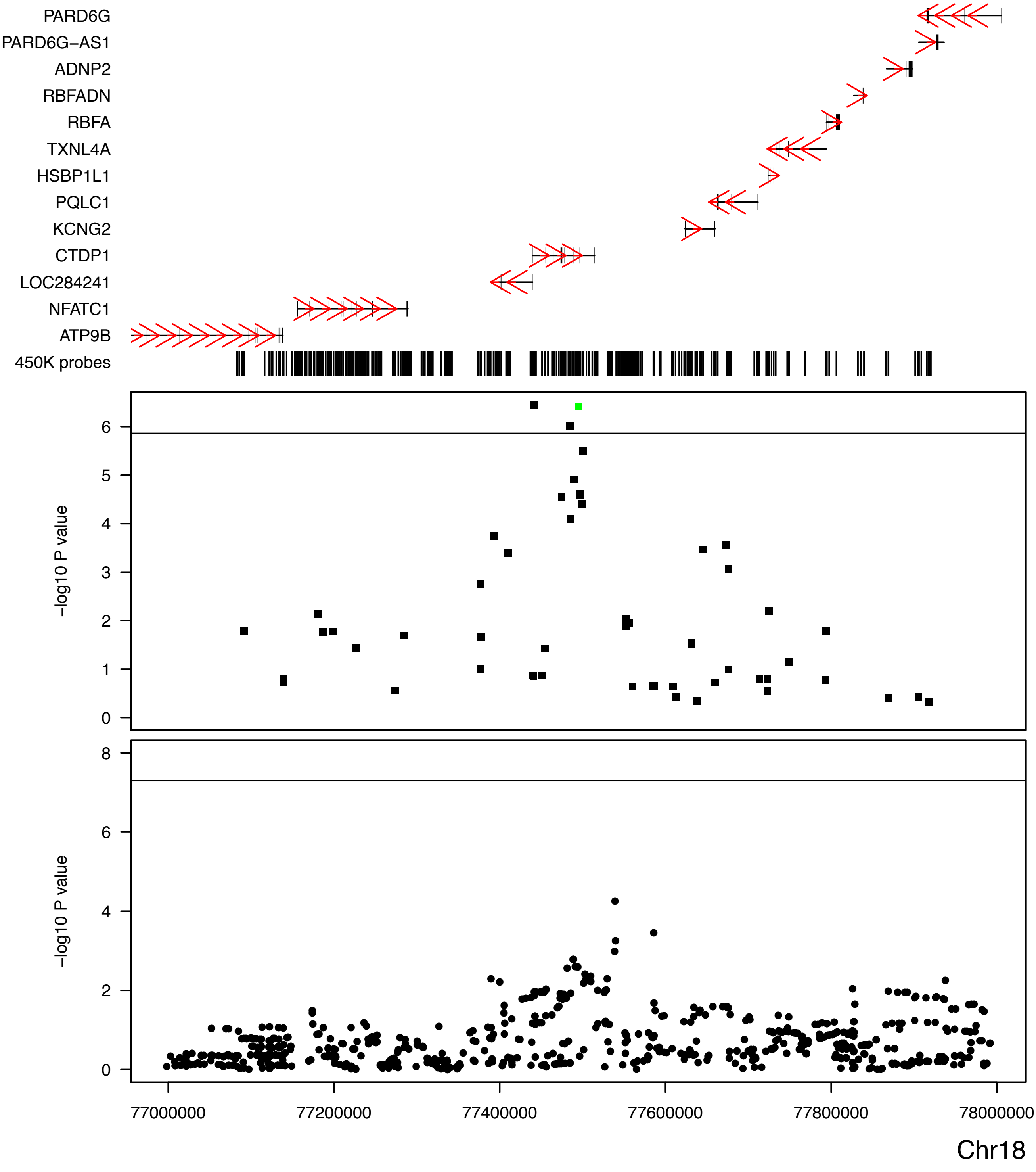
PARD6G
PARD6G-AS1
ADNP2
RBFADN
RBFA
TXNL4A
HSBP1L1
PQLC1
KCNG2
CTDP1
LOC284241
NFATC1
ATP9B
450K probes
-log10 P value
0
1
2
3
4
5
6
-log10 P value
0
2
4
6
8
77000000
77200000
77400000
77600000
77800000
78000000
Chr18

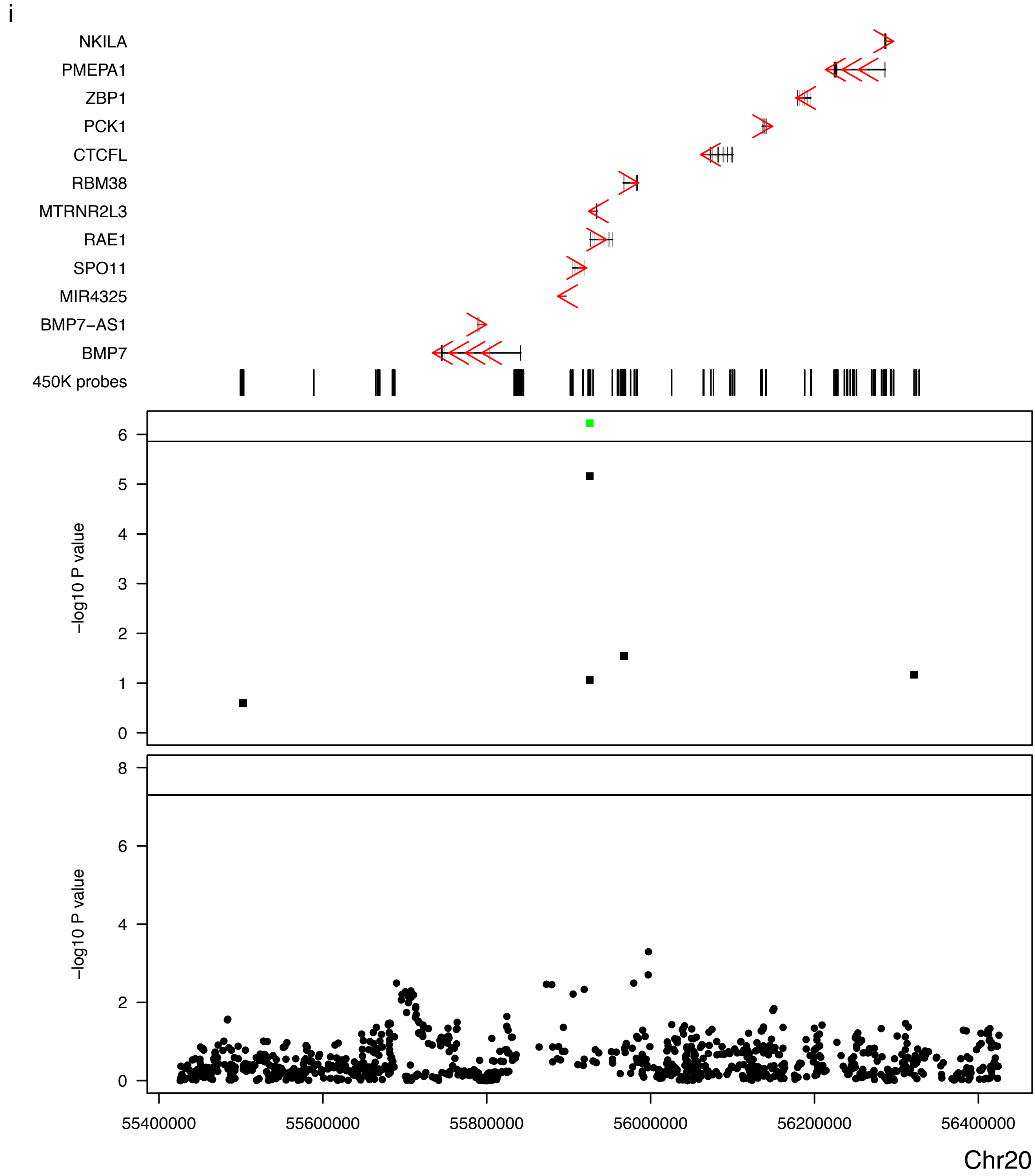

i
NKILA
PMEPA1
ZBP1
PCK1
CTCFL
RBM38
MTRNR2L3
RAE1
SPO11
MIR4325
BMP7−AS1
BMP7
450K probes
−log10 P value
0
1
2
3
4
5
6
−log10 P value
0
2
4
6
8
55400000
55600000
55800000
56000000
56200000
56400000
Chr20

Figure S4: Novel loci identified using SMR analysis for puberty (Tanner stage). Shown are examples where the SMR analysis identified a significant association between DNA methylation at **a**) cg06313718, **b**) cg06759629, cg01218619, cg19312667, cg02428792, cg26262055, and cg17688837, **c**) cg05987787, **d**) cg03595199, **e**) cg15417244, **f**) cg07075299, **g**) cg27409771, **h**) cg25357022, **i**) cg19836589 and Tanner stage. In each example, there is no association reported in the GWAS analysis within 0.5 Mb[2]. For each example, there is a gene track along the top and zoomed in Manhattan plots of the SMR analysis *P*-values (middle panel) and GWAS *P*-values (bottom panel). The black solid horizontal lines, indicate the multiple testing threshold for the SMR analysis ($P < 1.38x10^{-6}$) and GWAS ($P < 5x10^{-8}$); in the SMR Manhattan plots the green points indicate those characterized by a non-significant heterogeneity test ($P > 0.05$).

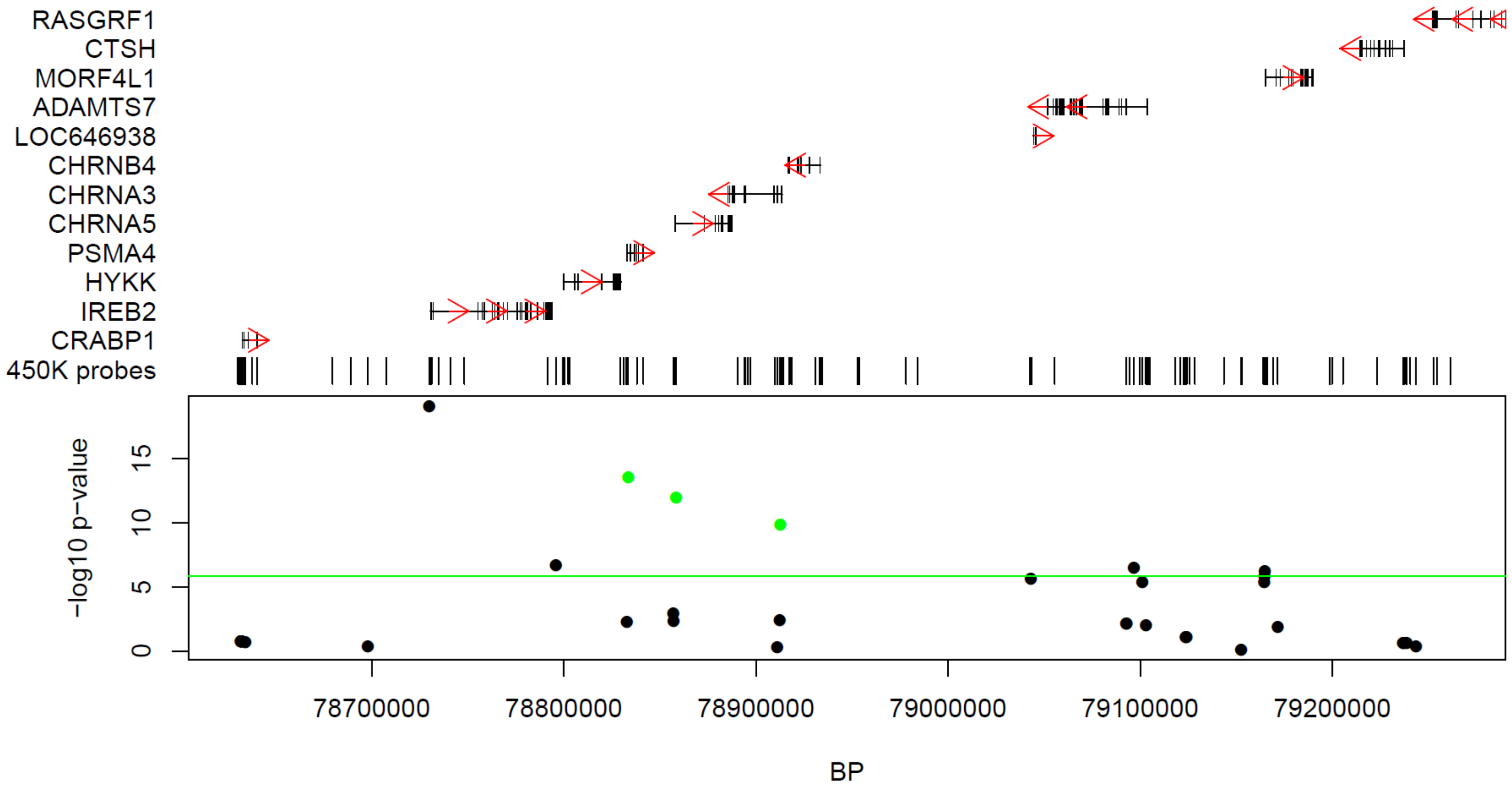

Figure S5: SMR analysis across a region on chromosome 15 identified by a GWAS analysis of cigerettes smoked per day. Shown on the y-axis is the SMR test –log10 *P*-value. Each point represents a SMR test for an individual DNA methylation site. The green horizontal line indicates the significance threshold ($P < 1.42 \times 10^{-6}$); green points highlight the significant SMR tests which did not show significant heterogeneity ($P > 0.05$); i.e. the genetic associations indicate a pleiotropic relationship between the complex trait and DNA methylation.

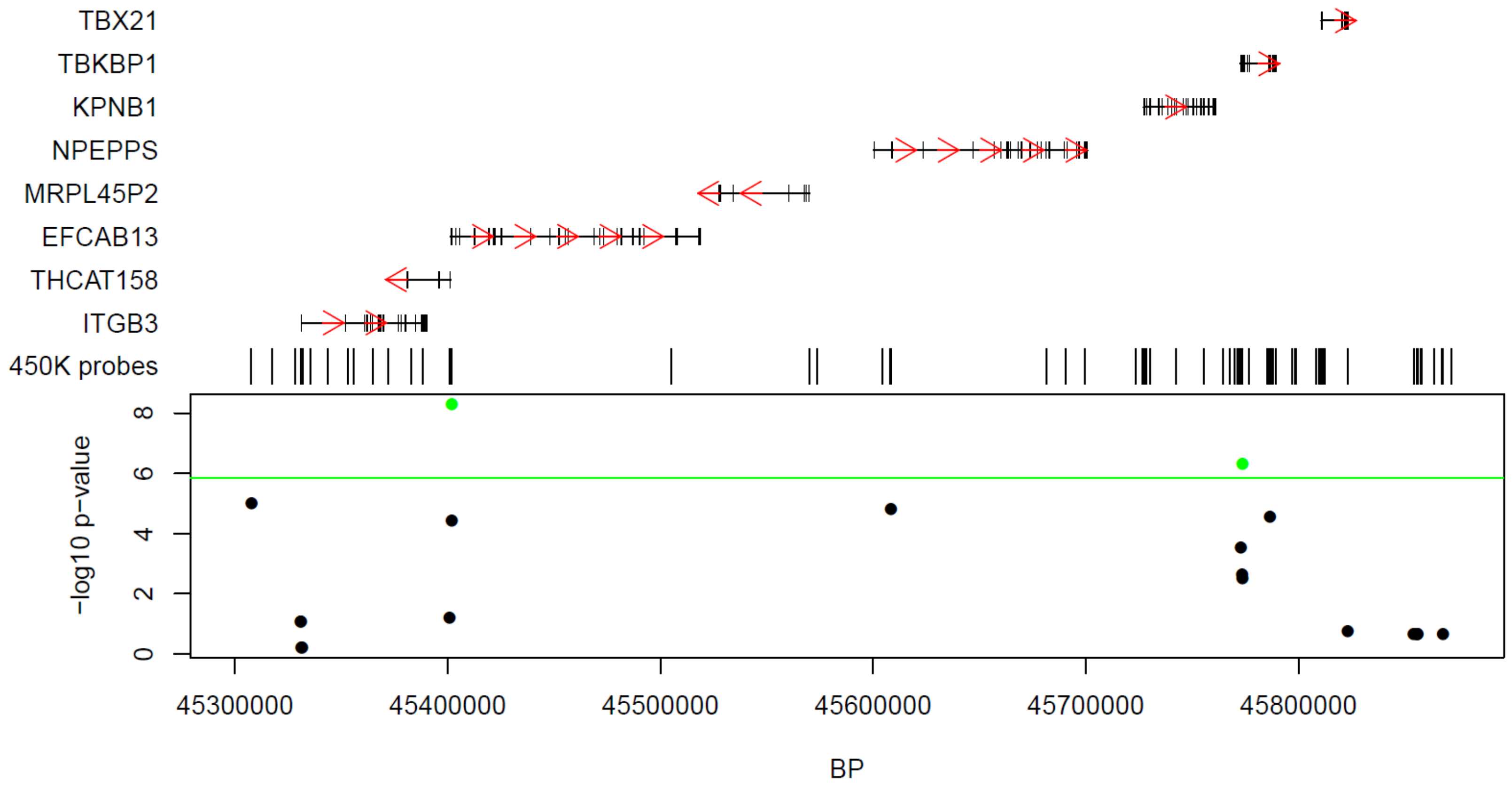

Figure S6: SMR analysis across a region on chromosome 17 identified by a GWAS analysis of total cholestrol. Shown on the y-axis is the SMR test –log10 *P*-value. Each point represents a SMR test for an individual DNA methylation site. The green horizontal line indicates the significance threshold ($P <$ 1.42 x 10^{-6}); green points highlight the significant SMR tests which did not show significant heterogeneity ($P > 0.05$); i.e. the genetic associations indicate a pleiotropic relationship between the complex trait and DNA methylation.

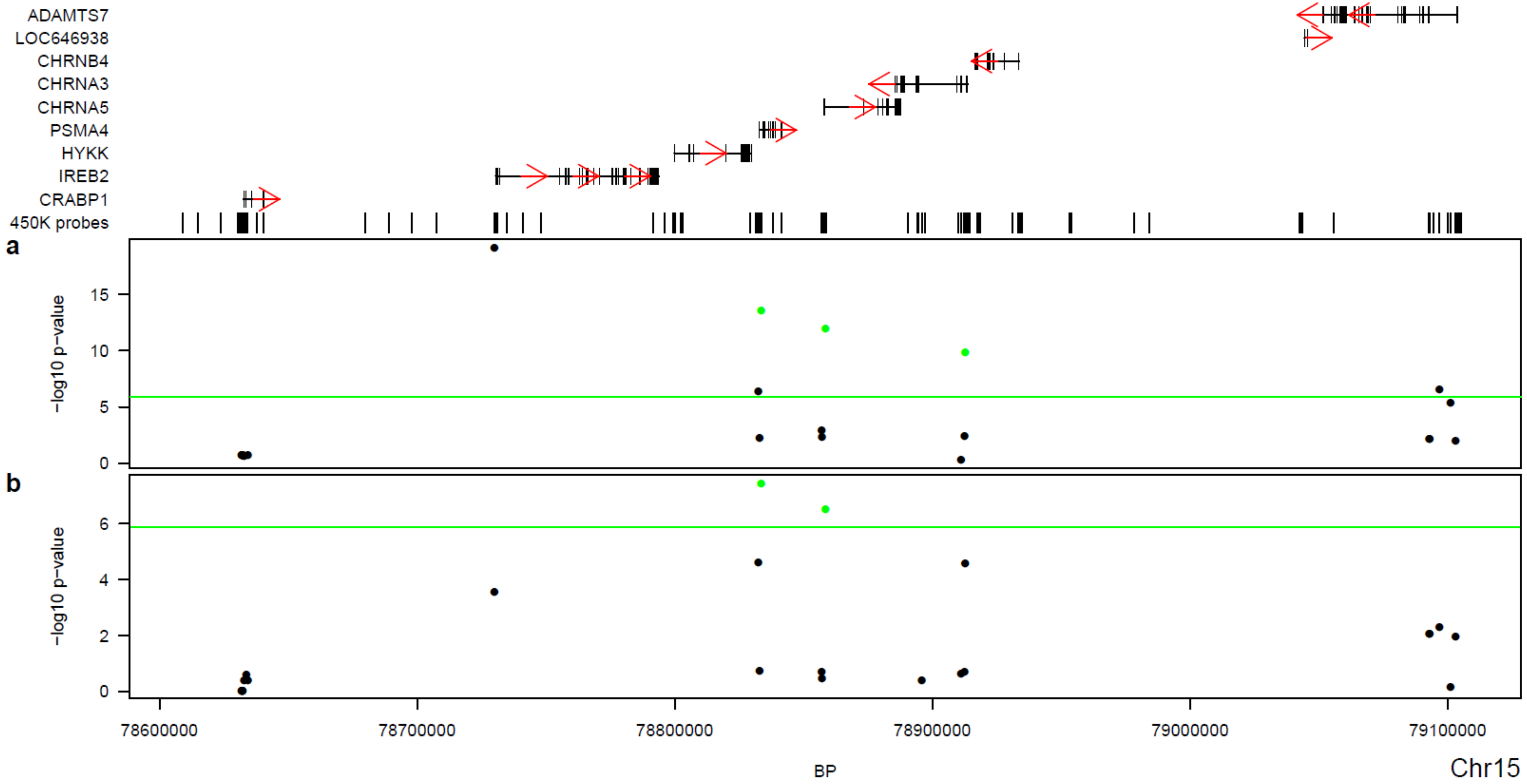

Figure S7: DNA methylation at CpG sites annotated to *CHRNA5* are associated with both schizophrenia and cigerettes smoked per day. Manhattan plots of SMR analysis across the *CHRNA5* locus where DNA methylation at cg24631222 and cg04140906 is associated with both **a**) schizophrenia[3] and **b**) cigarettes per day[4]. Shown on the y-axis is the SMR test –log10 *P*-value. Each point represents an SMR test for an individual DNA methylation site. The green horizontal line indicates the significance threshold ($P < 1.42 \times 10^{-6}$); green points highlight the significant SMR tests which were not characterized by significant heterogeneity ($P > 0.05$); i.e. the genetic associations indicate a pleiotropic relationship between the complex trait and DNA methylation.

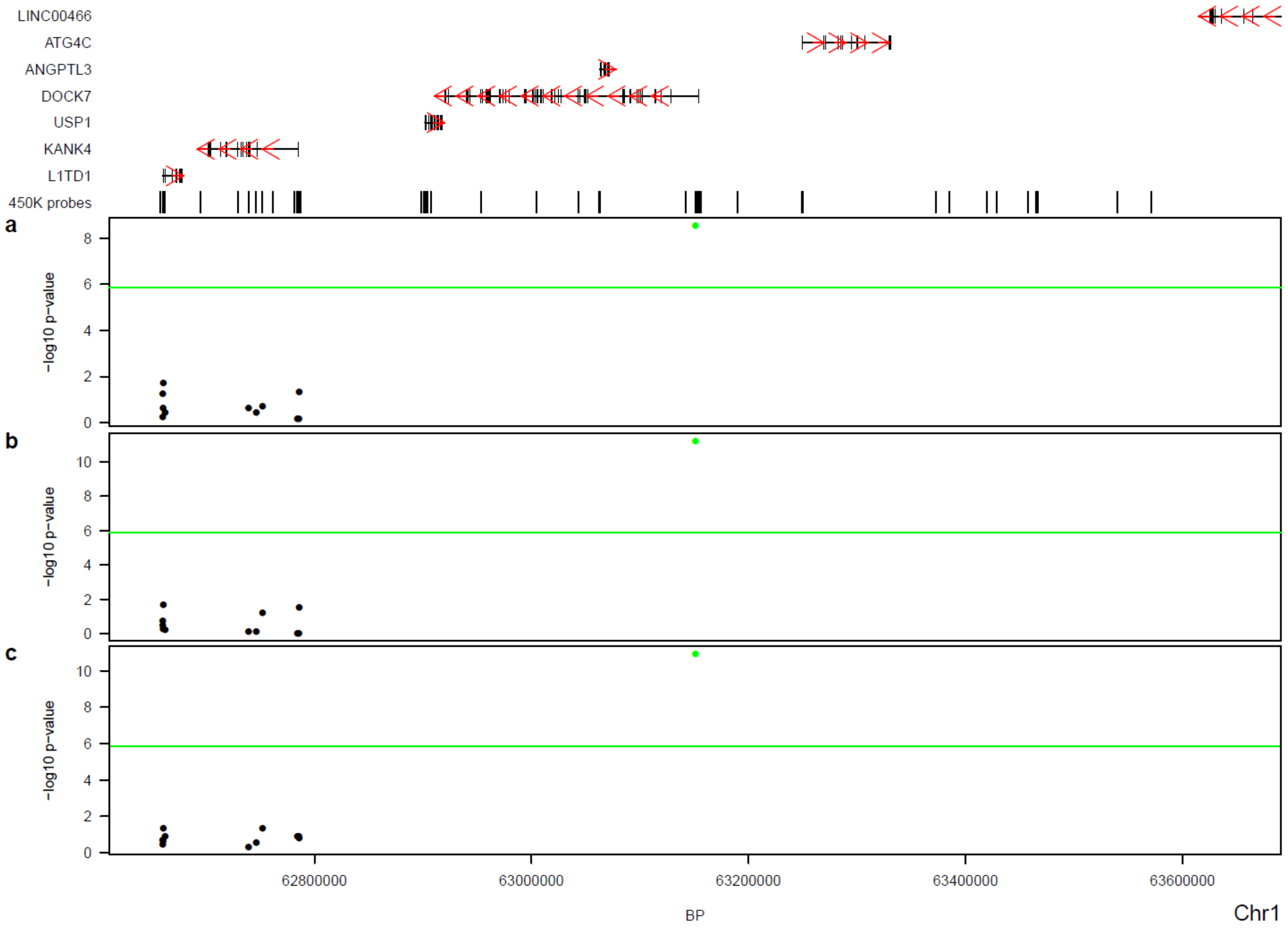

Figure S8: DNA methylation at *DOCK7* is associated with LDL, total cholesterol and triglycerides. Manhattan plots of SMR analysis across the *DOCK7* locus where DNA methylation at cg10583485 is associated with both **a**) LDL, **b**) total cholesterol and **c**) triglycerides[5]. Shown on the y-axis is the SMR test –log10 *P*-value. Each point represents an SMR test for an individual DNA methylation site. The green horizontal line indicates the significance threshold ($P < 1.42 \times 10^{-6}$); green points highlight the significant SMR tests which were not characterized by significant heterogeneity ($P > 0.05$); i.e. the genetic associations indicate a pleiotropic relationship between the complex trait and DNA methylation.

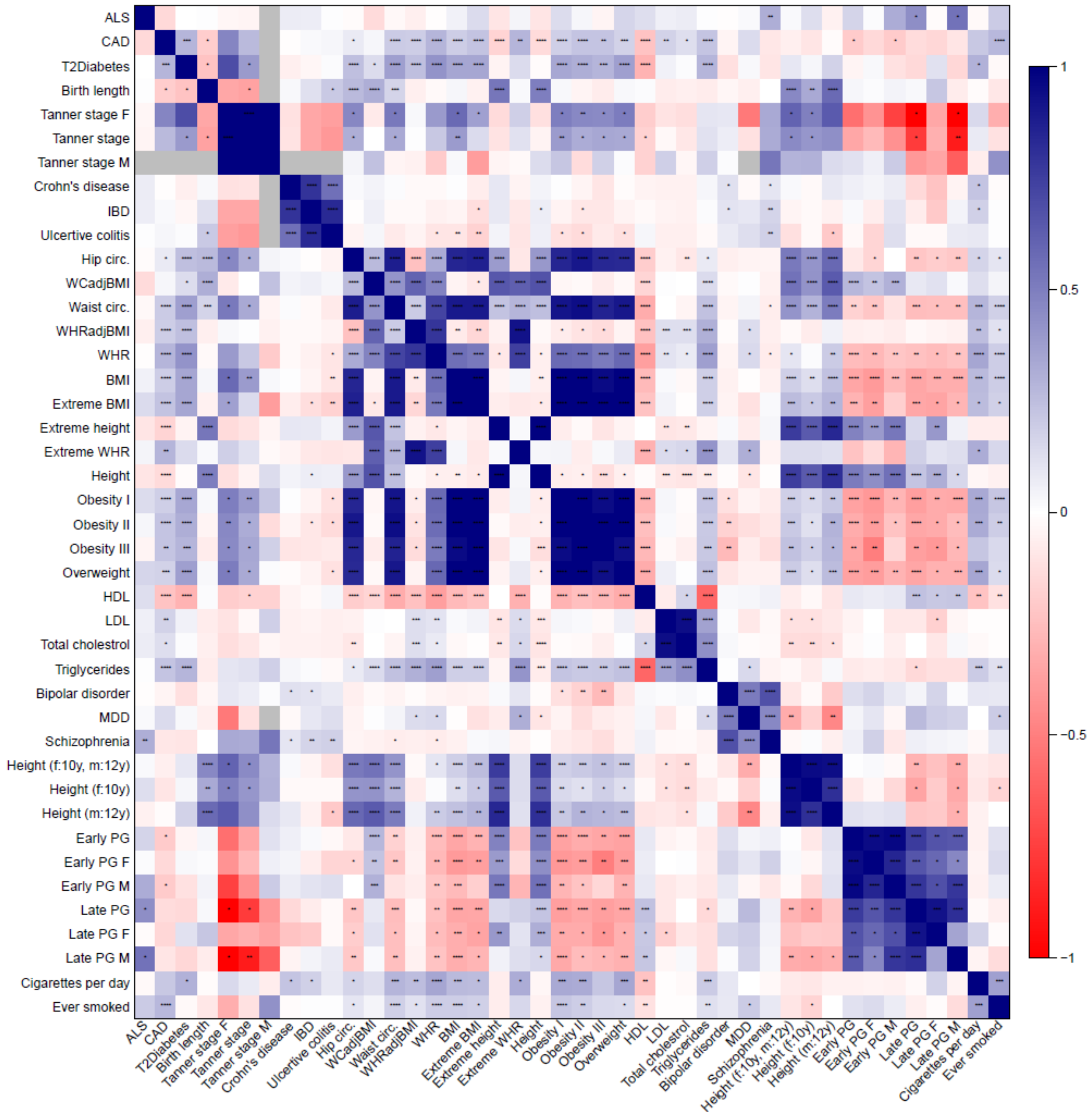

Figure S9: **A heat map of genetic correlations between complex traits considered for the SMR analysis.** The colour of each square represents the magnitude of the correlation between two traits, while asterisk indicate the significance of a non-zero correlation where * P < 0.05; ** P< 0.005; *** P < 0.0005; *** P < 0.00005. Genetic correlations were calculated using LDScore regression[6]. F-female; M-male; y – years; PG – pubertal growth; ALS – Amyloid lateral sclerosis; BMI – body mass index; CAD- coronary artery disease; WHR – waist hip ratio; IBD – inflammatory bowel disease; HDL/LDL – high/low density lipoprotein; MDD – major depressive disorder.

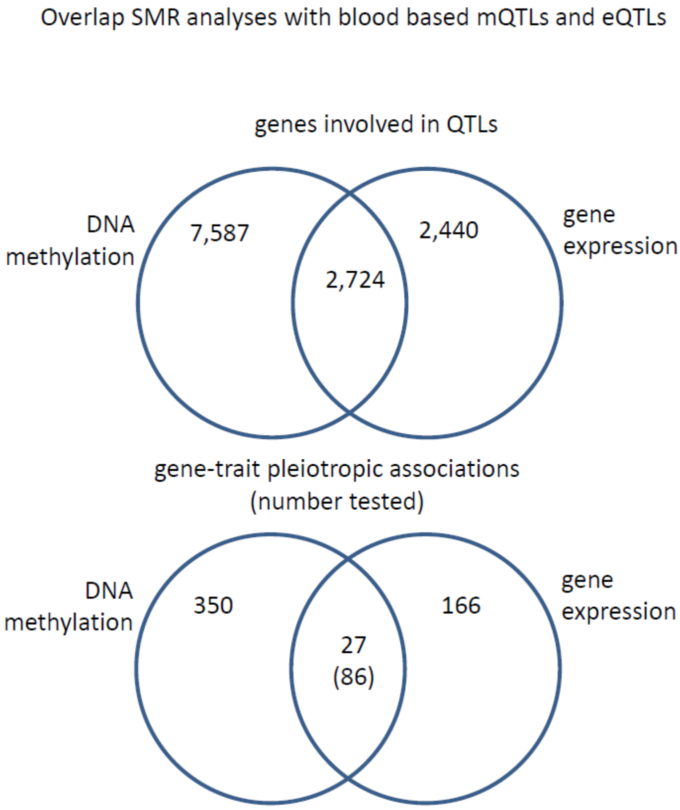

Figure S10: Flow diagram depicting the overlap of SMR analysis results obtained using blood mQTLs and blood eQTLs.

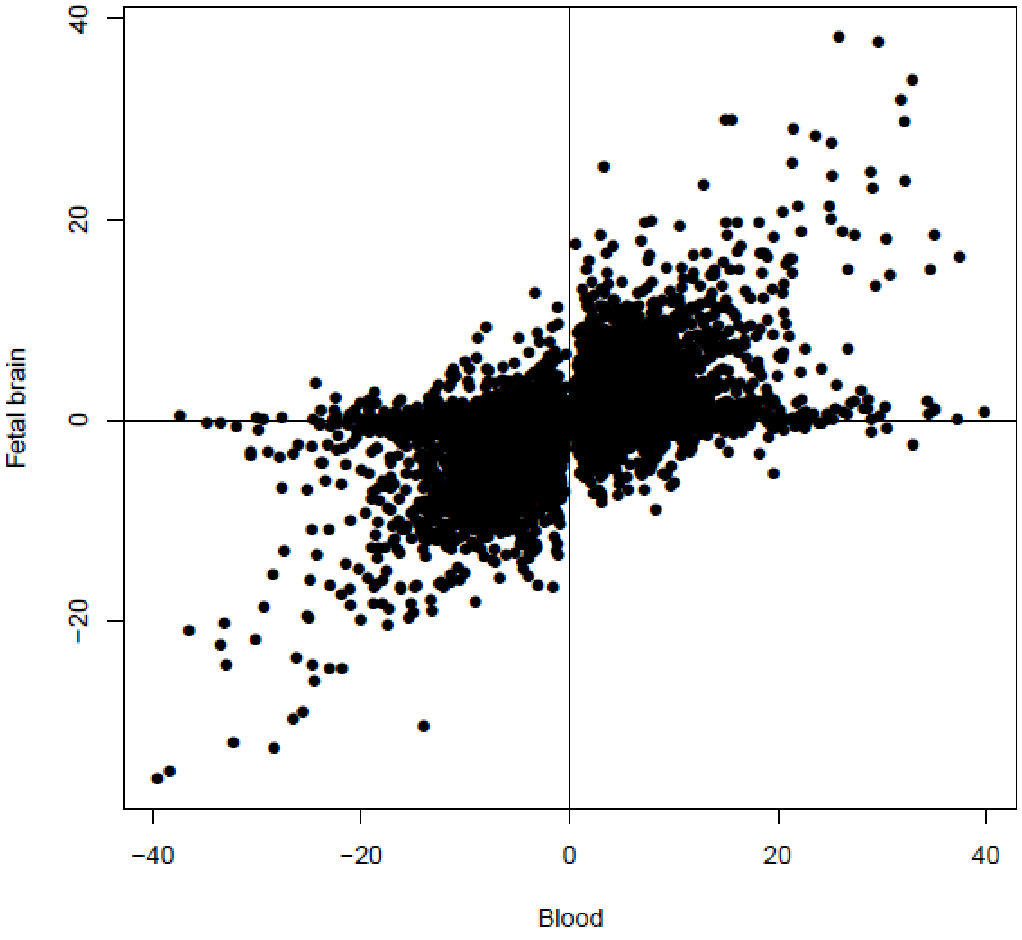

Figure S11: Scatterplot of effect sizes for mQTL identified in blood and tested fetal brain. All blood mQTL ($P < 1x\text{-}10^{-10}$) included in the SMR analysis were tested in fetal brain samples. Each point represents a SNP-DNA methylation site pairing, with the difference in DNA methylation per allele (%) shown for blood (x-axis) and fetal brain (y-axis).

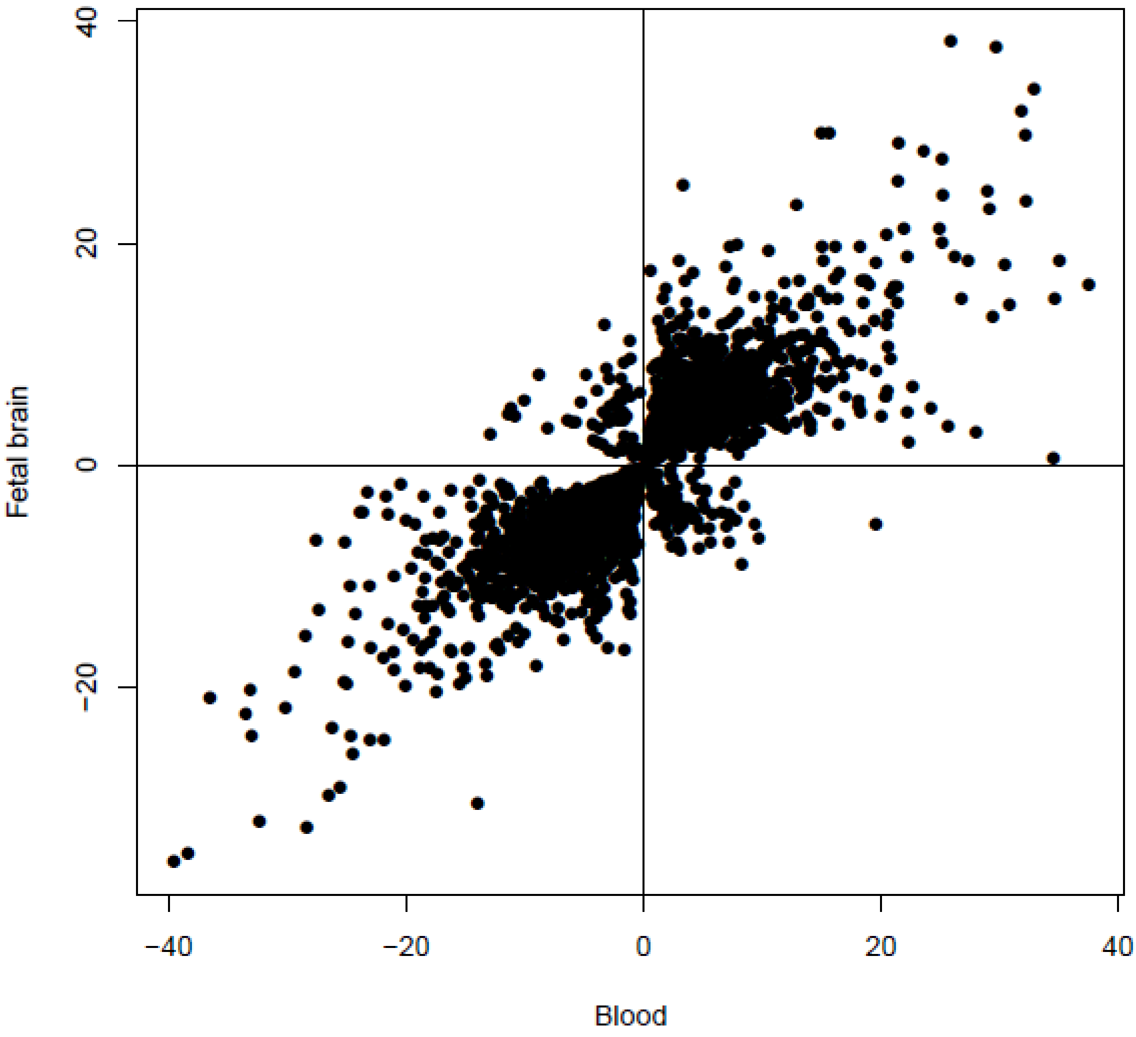

Figure S12: Scatterplot of effect sizes for mQTL identified and included in the SMR analysis for both blood and fetal brain. Each point represents a SNP-DNA methylation site pairing, with the difference in DNA methylation per allele (%) shown for blood (x-axis) and fetal brain (y-axis).

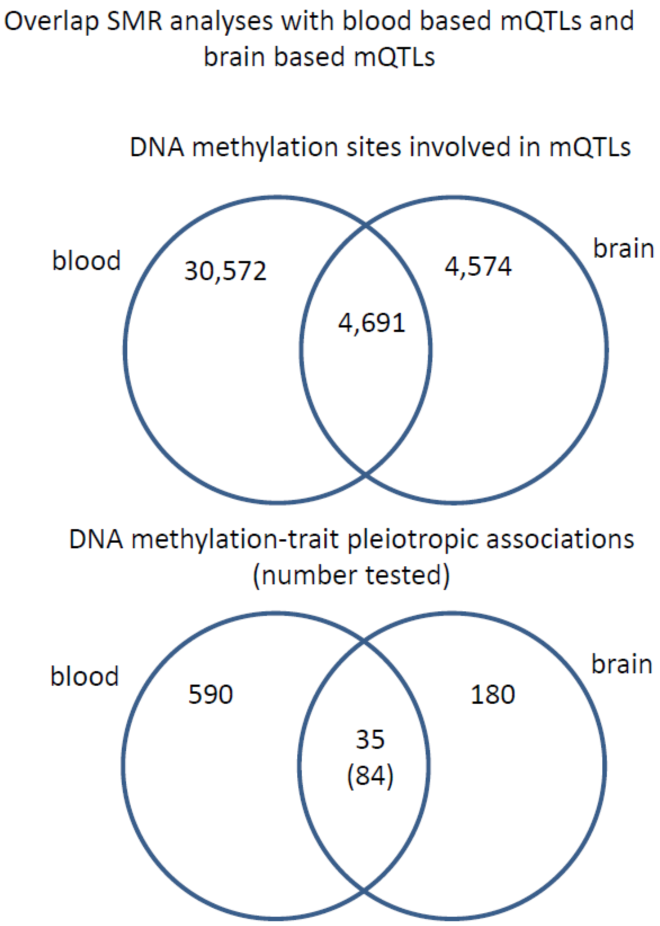

Figure S13: Flow diagram demonstrating the overlap of EWAS associations identified with SMR analysis using blood and fetal brain mQTLs.

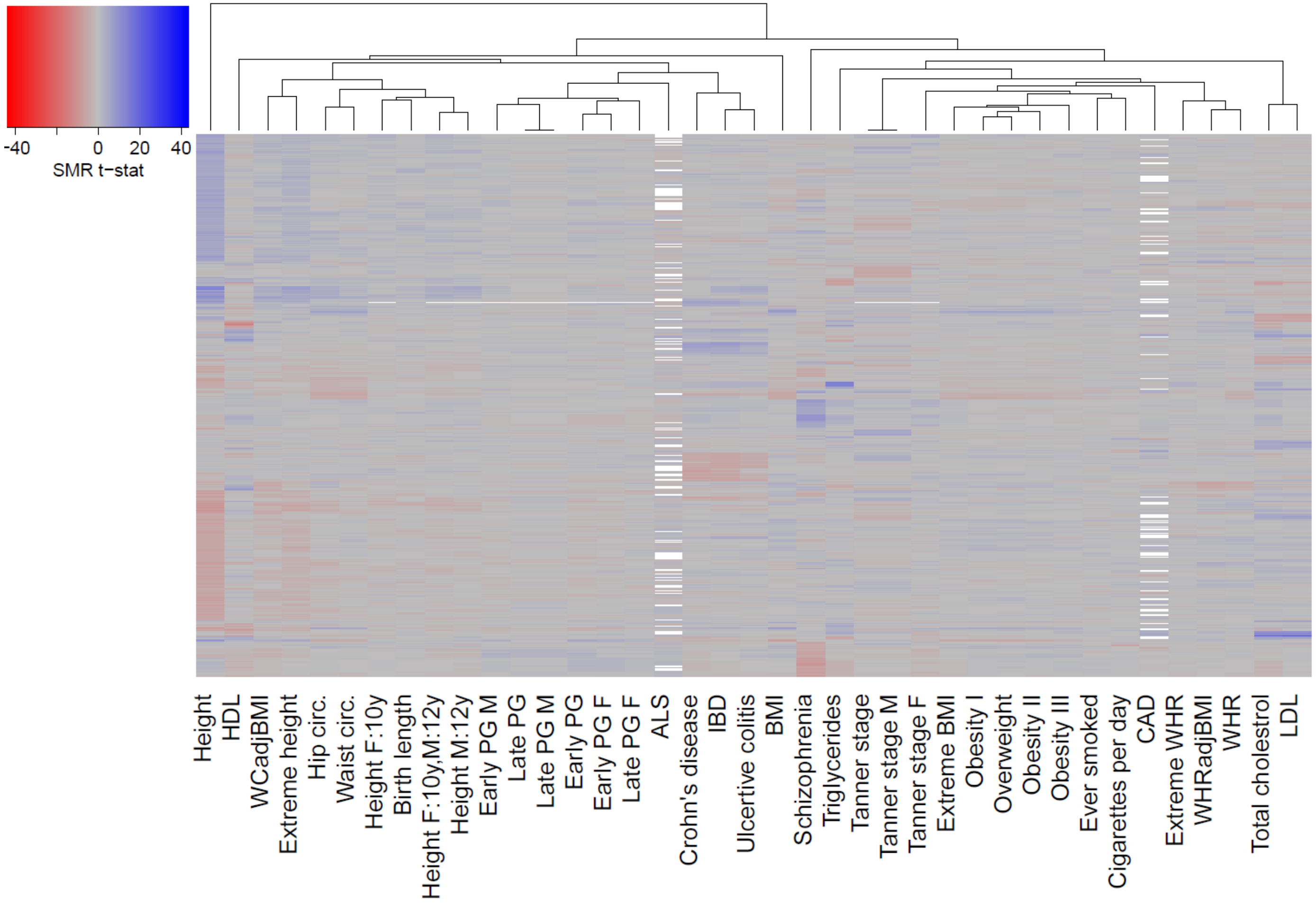

Figure S14: Heat-map of SMR probe associations across 38 GWAS traits. Shown is the t-statistic (b_SMR/se_SMR) of the GWAS trait (columns) for an individual DNA methylation site (row). Only traits (n = 38) tested against at least 20,000 DNA methylation sites were included in this analysis for DNA methylation sites (n = 1,044) associated with at least one phenotype. SMR- summarized mendelian randomization; WCadjBMI – waist circumference adjusted for body mass index; WHRadjBMI – waist hip ratio adjusted for body mass index; circ. – circumference; F-female; M-male; y – years; PG – pubertal growth; BMI – body mass index; CAD- coronary artery disease; WHR – waist hip ratio; IBD – inflammatory bowel disease; HDL/LDL – high/low density lipoprotein.

References

1. Hannon, E. *et al*. An integrated genetic-epigenetic analysis of schizophrenia: evidence for co-localization of genetic associations and differential DNA methylation. *Genome Biol* **17**, 176 (2016).
2. Cousminer, D.L. *et al*. Genome-wide association study of sexual maturation in males and females highlights a role for body mass and menarche loci in male puberty. *Hum Mol Genet* **23**, 4452-64 (2014).
3. Schizophrenia Working Group of the PGC *et al*. Biological insights from 108 schizophrenia-associated genetic loci. *Nature* **511**, 421-+ (2014).
4. Tobacco and Genetics Consortium. Genome-wide meta-analyses identify multiple loci associated with smoking behavior. *Nat Genet* **42**, 441-7 (2010).
5. Global Lipids Genetics Consortium *et al*. Discovery and refinement of loci associated with lipid levels. *Nat Genet* **45**, 1274-83 (2013).
6. Bulik-Sullivan, B. *et al*. An atlas of genetic correlations across human diseases and traits. *Nat Genet* **47**, 1236-41 (2015).
